# Supplementary material for: Weight cycling and the subsequent onset of type 2 diabetes mellitus: 10-year cohort studies in urban and rural Japan
Source: BMJ Open. 2017 Jun 8;7(5):e014684. doi: 10.1136/bmjopen-2016-014684 (PMC5729995; doi:10.1136/bmjopen-2016-014684)
Supplement: Supplementary file [file bmjopen-2016-014684supp001.pdf]

Supplementary. Baseline characteristics among groups of weight change patterns over 10 years

**Men in urban Japan (n = 10,094)**	Sustained loss	Loss-gain	Stable	Gain-loss	Sustained gain
Age, years	52.3 (12.2)	51.2 (12.0)	50.3 (11.3)	46.0 (11.4)	44.9 (11.3)
Weight, kg	70.8 (10.4)	69.8 (9.5)	68.3 (9.0)	67.3 (9.5)	67.8 (10.8)
Height, cm	170.1 (6.2)	170.3 (6.1)	170.2 (6.0)	170.7 (6.1)	171.2 (6.1)
Body mass index, kg/m2	24.4 (3.0)	24.0 (2.7)	23.5 (2.6)	23.1 (2.8)	23.1 (3.2)
HbA1c, %	5.4 (0.4)	5.4 (0.3)	5.4 (0.3)	5.3 (0.3)	5.3 (0.3)
HbA1c, mmol/mol	35.9 (3.9)	35.7 (3.7)	35.3 (3.7)	34.6 (3.7)	34.6 (3.4)
Fasting plasma glucose, mg/dL	102.3 (9.2)	101.5 (8.8)	101.0 (8.5)	99.0 (8.0)	99.8 (8.4)
Fasting plasma glucose, mmol/L	5.7 (0.5)	5.6 (0.5)	5.6 (0.5)	5.5 (0.4)	5.5 (0.5)
Weight change from 20 years of age, kg	+9.7 (9.1)	+9.0 (8.1)	+8.2 (7.9)	+7.0 (8.4)	+7.0 (20.5)
Current smoking, %	20.0	22.8	23.6	31.3	33.7
Usually drinking alcohol, %	61.7	62.3	63.8	57.3	55.2
Duration of walking per day, min	41.0 (38.3)	41.3 (41.7)	43.3 (41.5)	40.4 (35.2)	38.6 (36.0)
Physical activity of 6–7/week, %	11.2	12.0	11.9	10.4	11.6
**Women in urban Japan (n = 10,614)**	Sustained loss	Loss-gain	Stable	Gain-loss	Sustained gain
Age, years	52.4 (10.7)	51.1 (11.5)	48.9 (11.3)	45.7 (10.6)	43.5 (9.8)
Weight, kg	53.8 (8.0)	53.4 (7.8)	51.7 (7.0)	51.8 (7.1)	51.6 (7.4)
Height, cm	156.9 (5.7)	157.2 (5.9)	157.8 (5.6)	158.1 (5.5)	158.4 (5.3)
Body mass index, kg/m2	21.8 (3.0)	21.6 (3.0)	20.8 (2.7)	20.7 (2.7)	20.6 (2.8)
HbA1c, %	5.5 (0.4)	5.4 (0.4)	5.4 (0.3)	5.3 (0.3)	5.3 (0.3)
HbA1c, mmol/mol	36.1 (4.1)	35.4 (3.8)	35.0 (3.8)	34.4 (3.6)	34.0 (3.7)
Fasting plasma glucose, mg/dL	96.5 (8.9)	95.7 (8.1)	94.6 (8.0)	93.2 (7.3)	92.8 (7.3)

Fasting plasma glucose, mmol/L	5.4 (0.5)	5.3 (0.4)	5.3 (0.4)	5.2 (0.4)	5.2 (1.4)
Weight change from 20 years of age, kg	+4.1 (7.4)	+3.8 (7.3)	+2.4 (7.1)	+2.6 (7.1)	+2.2 (7.5)
Current smoking, %	6.6	6.7	5.7	9.9	11.8
Usually drinking alcohol, %	23.7	26.7	27.3	29.4	28.3
Duration of walking per day, min	42.9 (49.4)	41.2 (49.8)	42.3 (40.9)	41.5 (40.5)	43.8 (51.9)
Physical activity of 6–7/week, %	12.1	10.6	11.3	8.6	8.8
**Men in rural Japan (n = 4,818)**	Sustained loss	Loss-gain	Stable	Gain-loss	Sustained gain
Age, years	53.5 (10.3)	51.7 (9.8)	52.3 (10.1)	49.2 (10.1)	48.3 (10.5)
Weight, kg	67.2 (9.0)	66.2 (8.7)	66.0 (9.1)	64.7 (8.6)	64.3 (9.9)
Height, cm	167.5 (6.2)	168.0 (6.0)	167.8 (6.2)	168.5 (6.2)	168.8 (6.5)
Body mass index, kg/m2	23.9 (2.6)	23.4 (2.6)	23.4 (2.7)	22.8 (2.5)	22.5 (2.9)
HbA1c, %	5.3 (0.4)	5.3 (0.4)	5.3 (0.3)	5.2 (0.3)	5.2 (0.3)
HbA1c, mmol/mol	34.7 (3.9)	34.6 (3.9)	34.3 (3.7)	33.9 (3.8)	33.8 (3.5)
Fasting plasma glucose, mg/dL	97.5 (9.2)	96.4 (9.0)	96.9 (9.1)	95.1 (8.9)	95.1 (8.5)
Fasting plasma glucose, mmol/L	5.4 (0.5)	5.4 (0.5)	5.4 (0.5)	5.3 (0.5)	5.3 (0.5)
Current smoker, %	38.5	42.7	37.7	49.3	58.5
Current drinker, %	79.5	77.5	78.4	75.7	77.5
**Women in rural Japan (n = 4,852)**	Sustained loss	Loss-gain	Stable	Gain-loss	Sustained gain
Age, years	54.9 (9.0)	52.8 (8.6)	53.1 (9.2)	50.7 (9.2)	48.2 (9.9)
Weight, kg	54.0 (8.1)	53.6 (7.5)	52.9 (6.9)	52.6 (7.6)	52.6 (7.8)
Height, cm	154.1 (5.6)	154.8 (5.8)	154.6 (5.4)	155.3 (5.5)	155.7 (5.6)
Body mass index, kg/m2	22.7 (3.1)	22.4 (2.8)	22.1 (2.8)	21.8 (2.9)	21.7 (3.0)
HbA1c, %	5.3 (0.3)	5.3 (0.3)	5.3 (0.3)	5.3 (0.3)	5.2 (0.3)
HbA1c, mmol/mol	34.9 (3.7)	34.6 (3.4)	34.6 (3.6)	34.0 (3.4)	33.6 (3.3)

Fasting plasma glucose, mg/dL	94.1 (9.1)	93.0 (8.3)	93.7 (8.8)	93.0 (8.0)	91.9 (7.9)
Fasting plasma glucose, mmol/L	5.2 (0.5)	5.2 (0.5)	5.2 (0.5)	5.2 (0.4)	5.1 (0.4)
Current smoker, %	5.5	8.1	6.6	8.5	11.5
Current drinker, %	24.2	27.2	26.5	27.1	30.9

The data are presented as the mean (SD) or %.
