# Supplementary material for: Weight cycling and the subsequent onset of type 2 diabetes mellitus: 10-year cohort studies in urban and rural Japan
Source: BMJ Open. 2017 Jun 8;7(5):e014684. doi: 10.1136/bmjopen-2016-014684 (PMC5729995; doi:10.1136/bmjopen-2016-014684)
Supplement: Author's manuscript [file bmjopen-2016-014684.draft_revisions.pdf]

Weight change patterns and subsequent onset of type 2 diabetes mellitus in urban and rural Japan

Journal:	*BMJ Open*
Manuscript ID	bmjopen-2016-014684
Article Type:	Research
Date Submitted by the Author:	11-Oct-2016
Complete List of Authors:	Yokomichi, Hiroshi; University of Yamanashi, Department of Health Sciences<br>Ohde, Sachiko; St. Luke's International University, Center for Clinical Epidemiology<br>Takahashi, Osamu; St. Luke's International University, Center for Clinical Epidemiology<br>Mochizuki, Mie; University of Yamanashi, Department of Pediatrics<br>Takahashi, Atsunori; University of Yamanashi, Department of Health Sciences<br>Yoda, Yoshioki; Yamanashi Koseiren Health Care Center<br>Tsuji, Masahiro; Yamanashi Koseiren Health Care Center<br>Akiyama, Yuka; University of Yamanashi, Department of Health Sciences<br>Yamagata, Zentaro; The University of Yamanashi, Interdisciplinary Graduate School of Medicine and Engineering
**Primary Subject Heading**:	Diabetes and endocrinology
Secondary Subject Heading:	Health services research, Epidemiology, Nutrition and metabolism, Sports and exercise medicine
Keywords:	weight change, type 2 diabetes, body mass index, Asian, weight cycling

SCHOLARONE™
Manuscripts

Yokomichi H et al.

Weight change patterns and subsequent onset of type 2 diabetes mellitus in urban and rural Japan

Hiroshi Yokomichi[1*], Sachiko Ohde[2], Osamu Takahashi[2], Mie Mochizuki[3], Atsunori Takahashi[1], Yoshioki Yoda[4], Masahiro Tsuji[4], Yuka Akiyama[1] and Zentaro Yamagata[1]

[1]Department of Health Sciences, University of Yamanashi, Yamanashi, Japan

[2]Center for Clinical Epidemiology, St. Luke's International University, Tokyo, Japan

[3]Department of Pediatrics, University of Yamanashi, Yamanashi, Japan

[4]Yamanashi Koseiren Health Care Center, Yamanashi, Japan

[*]Corresponding author:

Hiroshi Yokomichi

1110 Shimokato, Chuo City, Yamanashi, 4093898, Japan

E-mail: hyokomichi@yamanashi.ac.jp

Tel: +81-(0)80-5524-7303

Keywords: weight change, type 2 diabetes, body mass index, Asian, weight cycling

Word count: 2,718

For peer review only - http://bmjopen.bmj.com/site/about/guidelines.xhtml

ABSTRACT

Objective: To investigate how weight change patterns impact the risk of diabetes, including weight change since 20 years of age.

Design: Cohort study.

Setting: Primary healthcare, urban and rural Japan.

Participants: 20,708 urban and 9,670 rural residents.

Primary outcome measure: Odds ratios (ORs) for diabetes in weight loss, loss–gain, stable, gain–loss and weight gain groups for 10 years. Weight gain and loss were defined as more than ±4% change from baseline weight.

Results: In the urban region, the ORs relative to the stable group for the loss–gain and gain–loss groups were 0.63 (95% CI: 0.45–0.89) and 0.51 (95%CI: 0.32–0.82) for men and 0.72 (95%CI: 0.39–1.34) and 1.05 (95%CI: 0.57–1.95) for women, respectively, whereas in the rural region, they were 1.58 (95%CI: 0.78–3.17) and 0.44 (95%CI: 0.15–1.29) in men and 0.41 (95%CI: 0.12–1.44) and 0.77 (95%CI: 0.28–2.14) in women, respectively. The ORs for an increase in weight of 5–10 kg since the age of 20 years were 1.54 (95%CI: 1.03–2.30) in men and 0.96 (95%CI: 0.55–1.65) in women.

Conclusions: Data from Japanese urban residents suggest that weight cycling reduces the risk of diabetes. These results differ from those in Western studies, perhaps owing to the motivations underlying the weight cycling.

Word count: 214 words

For peer review only

Strengths and limitations of this study

- In urban Japan, weight cyclers have a similar or lower risk of diabetes than people who maintain a constant body weight.
- In urban and rural Japan, sustained weight gain increases the risk of diabetes.
- There is a dose–response relationship in both sexes between weight gain since the age of 20 years and the risk of diabetes.
- Odds ratios may change according to the threshold of ±4% weight change in 10 years.
- Levels of insulin secretion and sensitivity were not measured.

For peer review only - http://bmjopen.bmj.com/site/about/guidelines.xhtml

INTRODUCTION

Weight gain is a well-known risk factor for incidental type 2 diabetes. Research in people of Western, Oriental and African descent has quantitatively established the risks of developing diabetes in relation to increases and decreases in weight.[1-3] Two major studies in the USA, the Framingham Heart Study and the National Health and Nutrition Examination Survey, have both reported that weight cyclers, people who repeatedly gain and lose weight over short periods, can be at an increased risk of diabetes.[4, 5] However, the risk of diabetes in Asian weight cyclers remains to be established.

Because of the preconception that links being slim to an aesthetic standard,[6, 7] many Asian women make efforts to lose weight.[8, 9] Another group likely to try to reduce weight are middle-aged East Asian businesspeople who have gained weight but have few opportunities for physical activity. Recent studies have demonstrated that East Asians are much more likely than Westerners to develop type 2 diabetes at a lower body mass index (BMI).[10] As a result, it may take only a small change in weight to alter the risk of diabetes of East Asians. The aim of this study was to establish whether weight cyclers residing in Japan were at an increased risk of diabetes. We used Japanese urban and rural data to examine this clinical question in populations with various lifestyles.

METHODS

Study participants and measurements

In this cohort study, we enrolled participants from an urban area, Tokyo Metropolis, and a rural area, Yamanashi Prefecture. In Tokyo, we enrolled employees of private companies who underwent medical check-ups between January 2005 and December 2014 at St. Luke's

For peer review only - http://bmjopen.bmj.com/site/about/guidelines.xhtml

International Hospital. These annual medical check-ups were based on a legal obligation imposed by the Industrial Safety and Health Act in Japan.[11] In Yamanashi Prefecture, we enrolled employees and residents who paid for a private comprehensive medical check-up service between April 1999 and March 2009 at the Yamanashi Koseiren Health Care Center. A subset of participants in Yamanashi used a subsidy from their employing companies or administrative agencies to receive the private medical check-up. Thus, data sets covering a 10-year period in two institutions were used in the research. Inclusion criteria included no diagnosis of diabetes and HbA1c less than 6.5% (48 mmol/mol) during a baseline period for the first 3 years of the 10-year period. Those included were also required to undergo a medical check-up at least twice during the outcome measurement period of the later seven years of the study. The onset of diabetes was identified through questionnaires for the diagnosis of diabetes, the commencement of diabetic therapies or glycated haemoglobin (HbA1c) ≥ 6.5% (48 mmol/mol).[12, 13] In the institution in Tokyo, trained nurses interviewed the participants from the age of 20 years to establish their changes in weight. BMI was calculated as the participant's weight in kilograms divided by the square of their height in metres.

Weight change categories

The participants were categorised into five groups according to their histories of weight change during the 3–10 years since the baseline period. The stable group comprised the participants whose weight had not changed from baseline by more than ±4%. The sustained gain group were the participants who gained more than 4% of their baseline weight and did not subsequently lose this extra weight. Similarly, the sustained loss group were the participants who lost more than 4% of their baseline weight and did not subsequently regain

For peer review only - http://bmjopen.bmj.com/site/about/guidelines.xhtml

this weight. The gain–loss group included the participants who gained more than 4% of their baseline weight but brought their weight back below +4%. The loss–gain group were the participants who lost more than 4% of their baseline weight but brought their weight back above −4%.

The ±4% change of weight used for this categorisation was determined as approximately a one-unit change for a person with a BMI of 22 kg/m^2. This was based on the 2014 reference mean BMIs for Japanese men and women of 23.6 kg/m^2 and 21.7 kg/m^2, respectively.[14] It also took into consideration the relatively short time period of ten years for observation. The gain–loss and loss–gain group participants were the weight cyclers of interest.

Statistical analysis

The baseline characteristics recorded for the participants were age, weight, height, BMI, HbA1c and fasting plasma glucose. The incidence of diabetes was measured after the participants were categorised, and any data measured after a diagnosis of diabetes were ignored to conserve temporality of exposure to outcome for an epidemiological causation.[15] We used univariate and multivariate logistic regressions to compare the risk of diabetes between the categorised groups. In the urban data of Tokyo Metropolis, covariates used for adjustment at baseline were age, weight change from 20 years of age, BMI, smoking habits, alcohol consumption and physical activity. In the rural data of Yamanashi Prefecture, the available covariates for adjustment at baseline were age, BMI, smoking habit and alcohol consumption. The analyses were stratified by sex. All statistical analyses were performed using SAS statistical software (version 9.3, SAS Institute, NC, USA). The descriptive statistics are reported as means and standard deviations (SD). All reported p values were 2-sided; P values of <0.05 were considered statistically significant.

For peer review only

RESULTS

Participants

For the multivariate analyses of primary interest, 10,094 men and 10,614 women were enrolled from Tokyo Metropolis (table 1). The means (SDs) of the baseline characteristics for the men in the urban region were as follows: age 49.6 (11.9) years, weight 68.8 (9.7) kg, BMI 23.7 (2.8) kg/m^2 and HbA1c 5.4% (0.3%) [35.3 (3.7) mmol/mol]. For the women in the urban region, these values were as follows: age 48.3 (11.3) years, weight 52.3 (7.4) kg, BMI 21.0 (2.9) kg/m^2 and HbA1c 5.3% (0.4%) [35.0 (3.8) mmol/mol].

Table 1 also shows the baseline characteristics of 4,818 men and 4,852 women enrolled from Yamanashi Prefecture. The baseline characteristics for the men in the rural region were as follows: age 51.2 (10.3) years, weight 65.7 (9.1) kg, BMI 23.2 (2.7) kg/m^2 and HbA1c 5.3% (0.3%) [34.3 (3.8) mmol/L]. For the women in the rural area, the values were as follows: age 52.1 (9.4) years, weight 53.1 (7.5) kg, BMI 22.1 (2.9) kg/m^2 and HbA1c 5.3% (0.3%) [34.4 (3.5) mmol/L].

Table 1. Baseline characteristics of the participants from urban (Tokyo Metropolis) and rural (Yamanashi Prefecture) regions of Japan.

Characteristics, mean (standard deviation)	Men	Women
**Urban region**		
No.	10,094	10,614
Age, years	49.6 (11.9)	48.3 (11.3)
Weight, kg	68.8 (9.7)	52.3 (7.4)
Height, cm	170.4 (6.1)	157.7 (5.6)
Body mass index, kg/m^2	23.7 (2.8)	21.0 (2.9)
HbA1c, %	5.4 (0.3)	5.3 (0.4)

For peer review only

(HbA1c, mmol/mol)	35.3 (3.7)	35.0 (3.8)
Fasting plasma glucose, mg/dL	100.8 (8.6)	94.5 (8.0)
(Fasting plasma glucose, mmol/L)	5.6 (0.5)	5.2 (0.4)
**Rural region**		
No.	4,818	4,852
Age, years	51.2 (10.3)	52.1 (9.4)
Weight, kg	65.7 (9.1)	53.1 (7.5)
Height, cm	168.1 (6.2)	154.8 (5.6)
Body mass index, kg/m^2	23.2 (2.7)	22.1 (2.9)
HbA1c, %	5.3 (0.3)	5.3 (0.3)
(HbA1c, mmol/mol)	34.3 (3.8)	34.4 (3.5)
Fasting plasma glucose, mg/dL	96.3 (9.0)	93.2 (8.5)
(Fasting plasma glucose, mmol/L)	5.3 (0.5)	5.2 (0.5)

Risk of diabetes in urban and rural Japan

Tables 2 and 3 present the incidence of diabetes and the odds ratios (ORs) for each explanatory variable in the urban and rural regions, respectively. For the men in the urban region, the adjusted ORs (95% confidence intervals [95%CIs]) compared to the stable group were 3.07 (2.15–4.39) in the sustained gain group, 0.51 (0.32–0.82) in the gain–loss group, 0.63 (0.45–0.89) in the loss–gain group and 1.11 (0.77–1.59) in the sustained loss group. For the women in the urban region, the adjusted ORs relative to the stable group were 7.00 (4.11–11.94) in the sustained gain group, 1.05 (0.57–1.95) in the gain–loss group, 0.72 (0.39–1.34) in the loss–gain group and 1.48 (0.80–2.74) in the sustained loss group. For the men in the rural region, the adjusted ORs were 3.15 (1.70–5.83) in the sustained gain group, 0.44 (0.15–1.29) in the gain–loss group, 1.58 (0.78–3.17) in the loss–gain group and 0.36 (0.12–1.05) in the sustained loss group. For the women in the rural region, the adjusted ORs were 1.43

For peer review only

(0.59–3.48) in the sustained gain group, 0.77 (0.28–2.14) in the gain–loss group, 0.41 (0.12–1.44) in the loss–gain group and 0.32 (0.09–1.10) in the sustained loss group.

Table 2. Incidence of diabetes mellitus (DM) over 10 years in an urban region of Japan and odds ratios related to patterns of weight change and other variables

Exposure variables		Acquired DM / No. of subjects (incidence, %)	Odds ratio (95% CI)	Adjusted odds ratio (95% CI)
**Men (No. For multivariate analysis =10,094)**				
Baseline age	Per 10 years	—	1.44 (1.33–1.55)	1.44 (1.29–1.61)
Weight change from 20 years of age, kg	< −5	7/1438 (0.5)	2.18 (1.14–4.18)	1.68 (0.75–3.80)
	−5 to +5	73/10646 (0.7)	Ref	Ref
	+5 to +10	31/3200 (1.0)	1.98 (1.45–2.72)	1.54 (1.03–2.30)
	> +10	78/2166 (3.6)	3.37 (2.54–4.48)	2.08 (1.40–3.10)
Baseline BMI, kg/m^2	< 18.5	6/387 (1.6)	1.35 (0.57–3.16)	0.88 (0.29–2.70)
	18.5–22	49/4236 (1.2)	Ref	Ref
	22–25	168/7220 (2.3)	2.04 (1.48–2.89)	1.73 (1.14–2.63)
	> 25	190/4699 (4.0)	3.60 (2.62–4.94)	2.52 (1.60–3.95)
Weight change pattern over 10 years	Sustained loss	66/1903 (3.5)	1.39 (1.03–1.87)	1.11 (0.77–1.59)
	Loss–gain	87/4644 (1.9)	0.74 (0.56–0.97)	0.63 (0.45–0.89)
	Stable	142/5621 (2.5)	Ref	Ref
	Gain–loss	38/3063 (1.2)	0.49 (0.34–0.70)	0.51 (0.32–0.82)
	Sustained gain	80/1311 (6.1)	2.51 (1.89–3.32)	3.07 (2.15–4.39)
Smoking	None	123/6385 (1.9)	Ref	Ref
	Ex-smoker	150/5940 (2.5)	1.32 (1.04–1.68)	0.97 (0.71–1.31)
	Current smoker	140/4217 (3.3)	1.75 (1.37–2.23)	1.73 (1.25–2.34)
Alcohol drinking	None	69/2250 (3.1)	Ref	Ref
	Sometimes	48/1667 (2.9)	0.94 (0.65–1.36)	0.96 (0.66–1.40)
	Usually	156/6177 (2.5)	0.82 (0.61–1.09)	0.78 (0.58–1.05)
Duration of walking per day	Per 30 min	—	0.996 (0.92–1.08)	1.01 (0.92–1.11)
Physical activity	0–1/week	85/3282 (2.6)	Ref	Ref

For peer review only

	1–2/week	108/4105 (2.6)	1.02 (0.76–1.36)	1.02 (0.76–1.36)
	3–5/week	43/1541 (2.8)	1.08 (0.74–1.57)	0.98 (0.67–1.45)
	6–7/week	37/1166 (3.2)	1.23 (0.83–1.82)	1.06 (0.69–1.62)
**Women (No. for multivariate analysis = 10,614)**				
Baseline age	Per 10 years	—	1.66 (1.48–1.87)	1.78 (1.50–2.12)
Weight change from 20 years of age, kg	< −5	11/439 (2.5)	0.71 (0.33–1.54)	0.48 (0.18–1.25)
	−5 to +5	61/5243 (1.2)	Ref	Ref
	+5 to +10	109/4779 (2.3)	1.42 (0.93–2.16)	0.96 (0.55–1.65)
	> +10	232/6081 (3.8)	5.41 (3.92–7.47)	2.10 (1.20–3.67)
Baseline BMI, kg/m^2	< 18.5	20/2916 (0.7)	1.24 (0.74–2.08)	1.47 (0.76–2.86)
	18.5–22	52/9388 (0.6)	Ref	Ref
	22–25	60/3676 (1.6)	2.98 (2.05–4.33)	2.01 (1.21–3.34)
	> 25	57/1470 (3.9)	7.24 (4.95–10.59)	2.91 (1.53–5.52)
Weight change pattern over 10 years	Sustained loss	24/1661 (1.4)	2.11 (1.25–3.54)	1.48 (0.80–2.74)
	Loss–gain	32/4022 (0.7)	1.15 (0.72–1.86)	0.72 (0.39–1.34)
	Stable	36/5212 (1.3)	Ref	Ref
	Gain–loss	30/4630 (0.6)	0.94 (0.58–1.53)	1.05 (0.57–1.95)
	Sustained gain	67/1925 (3.5)	5.19 (3.45–7.80)	7.00 (4.11–11.94)
Smoking	None	156/14,194 (1.1)	Ref	Ref
	Ex-smoker	17/1789 (1.0)	0.86 (0.52–1.1.43)	0.85 (0.45–1.61)
	Current smoker	16/1467 (1.1)	0.99 (0.59–1.67)	1.20 (0.63–2.32)
Alcohol drinking	None	78/5669 (1.4)	Ref	Ref
	Sometimes	20/2035 (1.0)	0.71 (0.43–1.17)	0.91 (0.55–1.52)
	Usually	27/2910 (0.9)	0.67 (0.43–1.04)	0.95 (0.60–1.51)
Duration of walking per day	Per 30 min	—	1.01 (0.93–1.11)	1.01 (0.90–1.13)
Physical activity	0–1/week	37/3894 (1.0)	Ref	Ref
	1–2/week	42/3760 (1.1)	1.18 (0.76–1.84)	1.11 (0.70–1.75)
	3–5/week	27/1874 (1.4)	1.52 (0.93–2.52)	1.14 (0.67–1.94)
	6–7/week	19/1086 (1.7)	1.86 (1.06–3.24)	1.43 (0.79–2.60)

DM, diabetes mellitus; CI, confidence interval; Ref, reference group; BMI, body mass index

For peer review only

For peer review only - http://bmjopen.bmj.com/site/about/guidelines.xhtml

Yokomichi H et al.

159 **Table 3. Incidence of diabetes mellitus (DM) over 10 years in a rural region of Japan**

160 **and odds ratios related to patterns of weight change and other variables**

Exposure variables		Acquired DM / No. of subjects (incidence, %)	Odds ratio (95% CI)	Adjusted odds ratio (95% CI)
**Men (No. for multivariate analysis = 4818)**				
Baseline age	Per 10 years	—	1.36 (1.08–1.72)	1.60 (1.24–2.05)
Baseline BMI, kg/m^2	< 18.5	0/167 (0)	—	—
	18.5–22	10/1425 (0.7)	Ref	Ref
	22–25	21/2079 (1.0)	1.44 (0.68–3.08)	1.69 (0.79–3.63)
	> 25	35/1148 (3.1)	4.45 (2.19–9.03)	5.81 (2.82–11.97)
Weight change pattern over 10 years	Sustained loss	4/725 (0.6)	0.43 (0.15–1.25)	0.36 (0.12–1.05)
	Loss–gain	13/681 (1.9)	1.50 (0.75–3.00)	1.58 (0.78–3.17)
	Stable	22/1719 (1.3)	Ref	Ref
	Gain–loss	4/916 (0.4)	0.34 (0.12–0.99)	0.44 (0.15–1.29)
	Sustained gain	23/778 (3.0)	2.35 (1.30–4.24)	3.15 (1.70–5.83)
Smoking	None	23/1695 (1.4)	Ref	Ref
	Ex-smoker	8/998 (0.8)	0.59 (0.26–1.32)	0.72 (0.32–1.64)
	Current smoker	35/2126 (1.7)	1.22 (0.72–2.07)	1.50 (0.85–2.62)
Drinking	None	11/1071 (1.0)	Ref	Ref
	Drinker	55/3748 (1.5)	1.44 (0.75–2.75)	1.52 (0.79–2.94)
**Women (No. for multivariate analysis = 4852)**				
Baseline age	Per 10 years	—	1.43 (0.99–2.06)	1.23 (0.83–1.84)
Baseline BMI, kg/m^2	< 18.5	0/411 (0)	—	—
	18.5–22	7/2135 (0.3)	Ref	Ref
	22–25	14/1563 (0.9)	2.75 (1.11–6.82)	2.69 (1.07–6.75)
	> 25	13/744 (1.8)	5.41 (2.15–13.60)	5.29 (2.07–13.51)
Weight change pattern over 10 years	Sustained loss	3/863 (0.3)	0.37 (0.11–1.29)	0.32 (0.09–1.10)
	Loss–gain	3/757 (0.4)	0.42 (0.12–1.47)	0.41 (0.12–1.44)
	Stable	15/1615 (0.9)	Ref	Ref
	Gain–loss	5/827 (0.6)	0.65 (0.24–1.79)	0.77 (0.28–2.14)
	Sustained gain	8/791 (1.0)	1.09 (0.46–2.58)	1.43 (0.59–3.48)
Smoking	None	2/646 (0.3)	Ref	Ref

For peer review only

For peer review only - http://bmjopen.bmj.com/site/about/guidelines.xhtml

	Ex-smoker	29/3822 (0.8)	2.45 (0.58–10.31)	2.19 (0.52–9.26)
	Current smoker	3/375 (0.8)	2.60 (0.43–15.60)	3.33 (0.55–20.28)
Drinking	None	31/3542 (0.9)	Ref	Ref
	Drinker	3/1311 (0.2)	0.26 (0.08–0.85)	0.29 (0.09–0.95)

DM, diabetes mellitus; CI, confidence interval; Ref, reference group; BMI, body mass index

DISCUSSION

The data for the urban region suggest that the risk of diabetes in male and female weight cyclers was similar to or lower than that in the participants who maintained a stable weight. In particular, the data for Japanese men residing in an urban region indicate that, compared with the men who maintained a stable weight, there was a significantly reduced risk of diabetes with weight cycling. The data for Japanese women residing in a rural region also suggest a reduced risk of diabetes with weight cycling compared with maintaining a stable weight.

These findings are not consistent with those from previous studies of Western populations, which showed that weight cycling increased the risk of diabetes. In the Framingham Heart Study, approximately 1 kg/m^2 of weight cycling in American men and women aged between 40 and 50 years carried a hazard ratio of 1.1 (95%CI: 0.8–1.5) for the risk of diabetes after adjustment for sex and BMI at 25 years of age.[4] In an American study of middle-aged women (the National Health and Nutrition Examination Survey), weight cycling of 4.5–9.1 kg and 9.1–22.2 kg with an intentional loss of weight three or more times in four years carried ORs for the risk of diabetes of 1.11 (95%CI: 0.89–1.37) and 1.39 (95%CI: 0.90–2.13), respectively.[5] A study from a cohort of medical students at the Johns Hopkins University School of Medicine reported that the highest quartile of BMI variability at ages between 25

For peer review only

and 45 years had an OR of 2.1 (95%CI: 1.0–4.6) for the risk of diabetes after 50 years, compared with the other three lower quartiles.[16]

Inconsistency between the Western studies and this study that weight cycling was not associated with an increase in the risk of diabetes but may even reduce the risk, may be due to ethnic differences in diet,[17] the capacity to gain weight[18] and self-consciousness about body weight.[19] Further research is needed to explore why the relationship between weight cycling and risk of diabetes is reversed between Western and East Asian populations. The reason may be attributable to different motivations to lose weight in the context of different diet cultures and body self-consciousness.[20] The East Asians who try to lose weight are particularly those who are relatively concerned about the poor health outcomes of being overweight. Westerners in the study cohorts described who tried to lose weight may, in an extreme expression, have been those who lost and regained a great deal of weight and potentially ran the risk of poor health outcomes.

From the data from the urban region, a weight increase of more than +5 kg above the participant's weight at the age of 20 years increased the risk of developing diabetes with a dose–response relationship in both men and women (table 2). Furthermore, in both sexes, an increase in weight of more than +10 kg above that when aged 20 years more than doubles the risk of diabetes, with statistical significance, compared to the risk for those who maintained their weight within ±5 kg of their weight when aged 20 years. These results agree with the study in a US cohort, which reported a relative risk of 3.2 (95%CI: 1.4–7.4) for the highest quartile of BMI increase from 25 to 45 years of age in comparison with the other three lower quartiles.[16] A dose–response relationship involving weight change from that aged 20 years occurred in the Japanese women residing in the urban region, with ORs of 0.48 (95%CI: 0.18–1.25) for a change less than 5 kg, 0.96 (95%CI: 0.55–1.65) for an increase of between 5

For peer review only - http://bmjopen.bmj.com/site/about/guidelines.xhtml

and 10 kg and 2.10 (95%CI: 1.20–3.67) for an increase greater than 10 kg. In contrast, the Japanese men who had lost 5 kg or more of weight between the age of 20 years and early middle age had an OR of 1.68 (95%CI: 0.75–3.80). This paradoxically increased OR was probably due to the small number of participants (seven) who developed diabetes among a weight loss group of 1438 people.

This study had several limitations. The first of these was the threshold of ±4% weight change in 10 years. This threshold referred to a study from United Kingdom on the association between weight change and the risk of diabetes.[21] The threshold of ±4% weight change was calculated as approximately 1 BMI unit in Japanese people with a mean BMI of 23 kg/m^2. However, the threshold for categorisation of weight cycling should vary according to mean BMIs in different ethnicities, in which people have different insulin sensitivities.[10, 22] Second, we did not evaluate insulin sensitivity. Measuring fasting plasma glucose and insulin concentration to calculate HOMA-IR,[23] an index of insulin resistance, would have allowed us to assess the association between weight cycling and the physiological hazard of diabetes. Third, the weight changes recorded in the rural region may be misclassified due to missing data for the years when participants did not undergo the health examination. However, such misclassification would bias the ORs to the null hypothesis, and we believe such a bias would not change the conclusions for the rural region. Finally, a subset of the diagnoses in this study was not made by physicians but by using an epidemiological criterion.[12] However, most observational studies in nature rely on epidemiological criteria for detecting diabetes, and the use of a consistent diagnostic criterion can allow researchers to compare the risk of diabetes onset between groups of reference and interest.

The present study also has several strengths. First, to the best of our knowledge, this is the first study to explore the relationship between weight cycling and the risk of diabetes in

For peer review only - http://bmjopen.bmj.com/site/about/guidelines.xhtml

Asians. Because the relationship was opposite to that in Americans, further research in East Asians is necessary to confirm the relationship. Next, this study was conducted in two differently characterised populations (urban and rural residents). The ORs of the risk of diabetes were 1.05 (95%CI: 0.57–1.95) in the weight gain–loss pattern of the urban resident women and 1.58 (95%CI: 0.78–3.17) in weight loss–gain pattern of the rural resident men. However, all other weight cycling patterns in both sexes in urban and rural regions were negatively correlated with the risk of diabetes. Third, the numbers of participants in both sexes were approximately 10,000 in the urban region and approximately 5,000 in the rural region. Because the present study included a large number of participants from both urban and rural Japan, Japanese and East Asian weight cyclers can refer to its results.

This study can contribute to helping public health practitioners and on-site clinical professionals prevent diabetes in the general population. A sustained weight gain greater than 4% over ten years in middle age and more than 5 kg of additional weight gained since the age of 20 years both carried increased risks of diabetes for both sexes. Because middle-aged people can easily undergo such a small weight gain over the short or long term, non-diabetic people within the normal BMI range should be cautious about the risks resulting from even such a slight weight gain through their lifetime. An interventional study indicated that weight loss (−1.8 kg/m^2 of BMI in a diet intervention group and −3.3 kg/m^2 of BMI in a diet-and-exercise intervention group) improved insulin sensitivity in Japanese patients with obesity and type 2 diabetes.[24] Improved insulin sensitivity was also observed in Americans who maintained their weight with treadmill exercise and no alteration of diet.[25] In addition, studies have demonstrated that building muscle through exercising without changing weight improves insulin sensitivity.[26]

For peer review only - http://bmjopen.bmj.com/site/about/guidelines.xhtml

In the present study, two profiles of weight cycling pattern resulted in an increase in the risk of diabetes, whereas in the other six profiles the risk decreased. Because better insulin sensitivity directly leads to a decreased risk of diabetes, the results may be due to the differences in how the participants lost or gained weight (i.e. whether they lost or gained fat or muscle mass). Recent studies have indicated that sarcopenia, the loss of skeletal muscle alone or with increased fat mass in ageing, is a leading cause of death in old age.[27] The present study results, in the context of previous studies, suggest that over both the short term and long term, people can reduce their risk of diabetes by losing fat and maintaining muscle mass.

CONCLUSIONS

In urban and rural Japanese populations, weight cycling appears to reduce the risk of diabetes in most patterns of weight change. The results may be attributable to the motivations of the weight cyclers. The risk of diabetes increases linearly with weight gain from the age of 20 years in Japanese urban men and women. A study that includes the measurement of insulin sensitivity is necessary to confirm the present results and to improve understanding of the risks for East Asian weight cyclers.

For peer review only - http://bmjopen.bmj.com/site/about/guidelines.xhtml

Acknowledgments We thank all participants in this study and staff at the St. Luke's International Hospital and the Yamanashi Koseiren Health Care Center for their support, which made this study possible.

Competing interests None declared.

Funding This work was supported by the Ministry of Education, Culture, Sports, Science and Technology of Japan (MEXT) (KAKENHI grant number: JP15K08730 and JP15K15221).

Contributors ZY, YY, MT and OT: setting up the study and data collection. HY, ZY, MM and AT: designing the study. HY and AT: data analysis. HY: writing and revising the draft. ZY, SO, MM, YA and HY: development of the discussion section. All authors read and approved the final manuscript.

Ethics approval The ethics committee of the School of Medicine, University of Yamanashi (approval number: H27-1417).

Data sharing statement No additional data are available.

For peer review only

REFERENCES

1. Nanri A, Mizoue T, Takahashi Y, et al. Association of weight change in different periods of adulthood with risk of type 2 diabetes in Japanese men and women: the Japan Public Health Center-Based Prospective Study. *J Epidemiol Commun H* 2011;65:1104–10.

2. Wannamethee SG, Shaper AG. Weight change and duration of overweight and obesity in the incidence of type 2 diabetes. *Diabetes Care* 1999;22:1266–72.

3. Ford ES, Williamson DF, Liu SM. Weight change and diabetes incidence: findings from a national cohort of US adults. *Am J Epidemiol* 1997;146:214–22.

4. Waring ME, Eaton CB, Lasater TM, et al. Incident diabetes in relation to weight patterns during middle age. *Am J Epidemiol* 2010;171:550–6.

5. Field AE, Manson JE, Laird N, et al. Weight cycling and the risk of developing type 2 diabetes among adult women in the United States. *Obes Res* 2004;12:267–74.

6. Mukai T, Crago M, Shisslak CM. Eating attitudes and weight preoccupation among female high school students in Japan. *J Child Psychol Psychiatry* 1994;35:677–88.

7. Swami V, Frederick DA, Aavik T, et al. The attractive female body weight and female body dissatisfaction in 26 countries across 10 world regions: results of the International Body Project I. *Personal Soc Psychol Bull* 2010;36:309–25.

8. Chisuwa N, O'Dea JA. Body image and eating disorders amongst Japanese adolescents. a review of the literature. *Appetite* 2010;54:5–15.

9. Xu X, Mellor D, Kiehne M, et al. Body dissatisfaction, engagement in body change behaviors and sociocultural influences on body image among Chinese adolescents. *Body Image* 2010;7:156–64.

10. Kodama K, Tojjar D, Yamada S, et al. Ethnic differences in the relationship between insulin sensitivity and insulin response: a systematic review and meta-analysis. *Diabetes Care* 2013;36:1789–96.

For peer review only

11. Ministry of Health, Labour and Welfare of Japan. Industrial Safety and Health Act (English version). 1972. http://www.japaneselawtranslation.go.jp/law/detail/?printID=&id=1926&re=&vm=02 (acceseed 11 Oct 2016).

12. American Diabetes Association. Diagnosis and classification of diabetes mellitus. *Diabetes Care* 2010;33:S62–S69.

13. The International Expert Committee. International Expert Committee report on the role of the A1C assay in the diagnosis of diabetes. *Diabetes Care* 2009;32:1327–34.

14. NCD Risk Factor Collaboration. Worldwide trends in diabetes since 1980: a pooled analysis of 751 population-based studies with 4· 4 million participants. *Lancet* 2016;387:1513–30.

15. Rothman KJ, Greenland S, Lash TL. Modern Epidemiology: 3rd ed. Lippincott Williams & Wilkins: New York; 2008: 26–31.

16. Brancati FL, Wang N, Mead LA, et al. Body weight patterns from 20 to 49 years of age and subsequent risk for diabetes mellitus: the Johns Hopkins Precursors Study. *Arch Int Med* 1999;159:957–63.

17. Slocum R. Race in the study of food. *Prog Hum Geogr* 2011;35:303–27.

18. The DECODE–DECODA Study Group. Age, body mass index and type 2 diabetes—associations modified by ethnicity. *Diabetologia* 2003;46:1063–70.

19. Mintz LB, Kashubeck S. Body image and disordered eating among Asian American and Caucasian college students an examination of race and gender differences. *Psychol Women Q* 1999;23:781–96.

20. Frederick DA, Forbes GB, Grigorian KE, et al. The UCLA Body Project I: gender and ethnic differences in self-objectification and body satisfaction among 2,206 undergraduates. *Sex Roles* 2007;57:317–27.

For peer review only - http://bmjopen.bmj.com/site/about/guidelines.xhtml

21. Wannamethee SG, Shaper AG, Walker M. Overweight and obesity and weight change in middle aged men: impact on cardiovascular disease and diabetes. *J Epidemiol Commun H* 2005;59:134–9.

22. Ntuk UE, Gill JMR, Mackay DF, et al. Ethnic-specific obesity cutoffs for diabetes risk: cross-sectional study of 490,288 UK Biobank participants. *Diabetes Care* 2014;37:2500–7.

23. Matthews D, Hosker J, Rudenski A, et al. Homeostasis model assessment: insulin resistance and β-cell function from fasting plasma glucose and insulin concentrations in man. *Diabetologia* 1985;28:412–9.

24. Yamanouchi K, Shinozaki T, Chikada K, et al. Daily walking combined with diet therapy is a useful means for obese NIDDM patients not only to reduce body weight but also to improve insulin sensitivity. *Diabetes Care* 1995;18:775–8.

25. Duncan GE, Perri MG, Theriaque DW, et al. Exercise training, without weight loss, increases insulin sensitivity and postheparin plasma lipase activity in previously sedentary adults. *Diabetes Care* 2003;26:557–62.

26. Nassis GP, Papantakou K, Skenderi K, et al. Aerobic exercise training improves insulin sensitivity without changes in body weight, body fat, adiponectin, and inflammatory markers in overweight and obese girls. *Metabolism* 2005;54:1472–9.

27. Fielding RA, Vellas B, Evans WJ, et al. Sarcopenia: an undiagnosed condition in older adults. Current consensus definition: prevalence, etiology, and consequences. International Working Group on Sarcopenia. *J Am Med Dir Assoc* 2011;12:249–56.

For peer review only

STROBE Statement—checklist of items that should be included in reports of observational studies

	Item No.	Recommendation	Page No.	Relevant text from manuscript
**Title and abstract**	1	(*a*) Indicate the study's design with a commonly used term in the title or the abstract	1	Line 1–2
		(*b*) Provide in the abstract an informative and balanced summary of what was done and what was found	2	Line 23–40
**Introduction**				
Background/rationale	2	Explain the scientific background and rationale for the investigation being reported	4	Line 53–66
Objectives	3	State specific objectives, including any prespecified hypotheses	4	Line 66–67
**Methods**				
Study design	4	Present key elements of study design early in the paper	4	Line 72–72
Setting	5	Describe the setting, locations, and relevant dates, including periods of recruitment, exposure, follow-up, and data collection	4	Line 72–73
Participants	6	(*a*) *Cohort study*—Give the eligibility criteria, and the sources and methods of selection of participants. Describe methods of follow-up<br>*Case-control study*—Give the eligibility criteria, and the sources and methods of case ascertainment and control selection. Give the rationale for the choice of cases and controls<br>*Cross-sectional study*—Give the eligibility criteria, and the sources and methods of selection of participants	5	Line 81–83
		(*b*) *Cohort study*—For matched studies, give matching criteria and number of exposed and unexposed<br>*Case-control study*—For matched studies, give matching criteria and the number of controls per case		NA
Variables	7	Clearly define all outcomes, exposures, predictors, potential confounders, and effect modifiers. Give diagnostic criteria, if applicable	5–6	Line 85–87, 93–102
Data sources/ measurement	8*	For each variable of interest, give sources of data and details of methods of assessment (measurement). Describe comparability of assessment methods if there is more than one group	5–6	Line 93–102
Bias	9	Describe any efforts to address potential sources of bias	5	Line 83–90
Study size	10	Explain how the study size was arrived at	7	Line 126, 132

Continued on next page

For peer review only - http://bmjopen.bmj.com/site/about/guidelines.xhtml

Quantitative variables	11	Explain how quantitative variables were handled in the analyses. If applicable, describe which groupings were chosen and why	6	Line 103–107
Statistical methods	12	(*a*) Describe all statistical methods, including those used to control for confounding	6	Line 114–119
		(*b*) Describe any methods used to examine subgroups and interactions	6	Line 119
		(*c*) Explain how missing data were addressed	5	Line 83–85
		(*d*) *Cohort study*—If applicable, explain how loss to follow-up was addressed<br>*Case-control study*—If applicable, explain how matching of cases and controls was addressed<br>*Cross-sectional study*—If applicable, describe analytical methods taking account of sampling strategy		NA
		(*e*) Describe any sensitivity analyses		NA
**Results**				
Participants	13*	(a) Report numbers of individuals at each stage of study—eg numbers potentially eligible, examined for eligibility, confirmed eligible, included in the study, completing follow-up, and analysed	7	Line 126, 132
		(b) Give reasons for non-participation at each stage	5	Line 83–85
		(c) Consider use of a flow diagram		NA
Descriptive data	14*	(a) Give characteristics of study participants (eg demographic, clinical, social) and information on exposures and potential confounders	7–8	Table 1
		(b) Indicate number of participants with missing data for each variable of interest		NA
		(c) *Cohort study*—Summarise follow-up time (eg, average and total amount)		NA
Outcome data	15*	*Cohort study*—Report numbers of outcome events or summary measures over time	9–12	Table 2 and 3
		Case-control study—Report numbers in each exposure category, or summary measures of exposure		NA
		Cross-sectional study—Report numbers of outcome events or summary measures		NA
Main results	16	(*a*) Give unadjusted estimates and, if applicable, confounder-adjusted estimates and their precision (eg, 95% confidence interval). Make clear which confounders were adjusted for and why they were included	9–12	Table 2 and 3
		(*b*) Report category boundaries when continuous variables were categorized	9–12 (BMI)	Table 2 and 3
		(*c*) If relevant, consider translating estimates of relative risk into absolute risk for a meaningful time period		NA

For peer review only - http://bmjopen.bmj.com/site/about/guidelines.xhtml

Continued on next page Other analyses	17	Report other analyses done—eg analyses of subgroups and interactions, and sensitivity analyses		Table 2 and 3
**Discussion**				
Key results	18	Summarise key results with reference to study objectives	12	Line 164–170
Limitations	19	Discuss limitations of the study, taking into account sources of potential bias or imprecision. Discuss both direction and magnitude of any potential bias	14	Line 210–226
Interpretation	20	Give a cautious overall interpretation of results considering objectives, limitations, multiplicity of analyses, results from similar studies, and other relevant evidence	12–14	Line 171–209
Generalisability	21	Discuss the generalisability (external validity) of the study results	15	Line 230–231
**Other information**				
Funding	22	Give the source of funding and the role of the funders for the present study and, if applicable, for the original study on which the present article is based	17	Line 273–274

*Give information separately for cases and controls in case-control studies and, if applicable, for exposed and unexposed groups in cohort and cross-sectional studies.

Note: An Explanation and Elaboration article discusses each checklist item and gives methodological background and published examples of transparent reporting. The STROBE checklist is best used in conjunction with this article (freely available on the Web sites of PLoS Medicine at http://www.plosmedicine.org/, Annals of Internal Medicine at http://www.annals.org/, and Epidemiology at http://www.epidem.com/). Information on the STROBE Initiative is available at www.strobe-statement.org.

For peer review only

For peer review only - http://bmjopen.bmj.com/site/about/guidelines.xhtml

BMJ Open

Weight cycling and the subsequent onset of type 2 diabetes mellitus in urban and rural Japan

Journal:	*BMJ Open*
Manuscript ID	bmjopen-2016-014684.R1
Article Type:	Research
Date Submitted by the Author:	17-Jan-2017
Complete List of Authors:	Yokomichi, Hiroshi; University of Yamanashi, Department of Health Sciences<br>Ohde, Sachiko; St. Luke's International University, Center for Clinical Epidemiology<br>Takahashi, Osamu; St. Luke's International University, Center for Clinical Epidemiology<br>Mochizuki, Mie; University of Yamanashi, Department of Pediatrics<br>Takahashi, Atsunori; University of Yamanashi, Department of Health Sciences<br>Yoda, Yoshioki; Yamanashi Koseiren Health Care Center<br>Tsuji, Masahiro; Yamanashi Koseiren Health Care Center<br>Akiyama, Yuka; University of Yamanashi, Department of Health Sciences<br>Yamagata, Zentaro; The University of Yamanashi, Interdisciplinary Graduate School of Medicine and Engineering
**Primary Subject Heading**:	Diabetes and endocrinology
Secondary Subject Heading:	Health services research, Epidemiology, Nutrition and metabolism, Sports and exercise medicine
Keywords:	weight cycling, weight change, type 2 diabetes, body mass index, Asian

SCHOLARONE™
Manuscripts

Weight cycling and the subsequent onset of type 2 diabetes mellitus in urban and rural Japan

Hiroshi Yokomichi[1*], Sachiko Ohde[2], Osamu Takahashi[2], Mie Mochizuki[3], Atsunori Takahashi[1], Yoshioki Yoda[4], Masahiro Tsuji[4], Yuka Akiyama[1] and Zentaro Yamagata[1]

[1]Department of Health Sciences, University of Yamanashi, Yamanashi, Japan

[2]Center for Clinical Epidemiology, St. Luke's International University, Tokyo, Japan

[3]Department of Pediatrics, University of Yamanashi, Yamanashi, Japan

[4]Yamanashi Koseiren Health Care Center, Yamanashi, Japan

[*]Corresponding author:

Hiroshi Yokomichi

1110 Shimokato, Chuo City, Yamanashi, 4093898, Japan

E-mail: hyokomichi@yamanashi.ac.jp

Tel: +81-(0)80-5524-7303

Keywords: weight cycling; weight change; type 2 diabetes; body mass index; Asian

Word count: 3,243

For peer review only - http://bmjopen.bmj.com/site/about/guidelines.xhtml

ABSTRACT

Objective: To investigate how patterns in weight changes impact the risk of diabetes, including those that occur after 20 years.

Design: Cohort study.

Setting: Primary healthcare in urban and rural Japan.

Participants: 20,708 urban and 9,670 rural residents.

Primary outcome measures: Odds ratios (ORs) for diabetes in weight loss, loss-gain, stable, gain-loss and weight gain for 10 years. Weight gain and loss were defined as a greater than ±4% change from baseline weight.

Results: In the urban region, the ORs relative to the stable group for the loss-gain and gain-loss groups were 0.63 (95% CI: 0.45–0.89) and 0.51 (95% CI: 0.32–0.82) for men and 0.72 (95% CI: 0.39–1.34) and 1.05 (95% CI: 0.57–1.95) for women, respectively. In the rural region, they were 1.58 (95% CI: 0.78–3.17) and 0.44 (95% CI: 0.15–1.29) in men and 0.41 (95% CI: 0.12–1.44) and 0.77 (95% CI: 0.28–2.14) in women, respectively. The ORs for an increase in weight between 5–10 kg from the age of 20 years were 1.54 (95% CI: 1.03–2.30) in men and 0.96 (95% CI: 0.55–1.65) in women.

Conclusions: In Japan, weight cycling is associated with a significant reduction in the risk of diabetes for men from an urban region and non-significantly for women from a rural region. However, the associations are unclear for women from the urban region and in men of the rural region. These results differ from those in Western studies, likely due to the motivations underlying weight cycling.

For peer review only - http://bmjopen.bmj.com/site/about/guidelines.xhtml

Word count: 251 words (limit: 300 words)

For peer review only

For peer review only - http://bmjopen.bmj.com/site/about/guidelines.xhtml

Strengths and limitations of this study

- In urban Japan, weight cyclers have a similar or lower risk of diabetes than people who maintain a constant body weight.
- In urban and rural Japan, sustained weight gain increases the risk of diabetes.
- There is a dose–response relationship for both sexes between weight gain since the age of 20 years and the risk of diabetes.
- Odds ratios may change according to the threshold of ±4% weight change in 10 years.
- The levels of insulin secretion and sensitivity were not measured.

For peer review only

INTRODUCTION

Weight gain is a well-known risk factor for incidental type 2 diabetes. Research involving people of Western, Oriental and African descent has quantitatively established the risks of developing diabetes in relation to weight gain.[1-3] Researchers have also raised the question of whether repeatedly gaining and losing weight (weight cycling) is an independent risk factor for developing diabetes due to gaining weight. Studies on this topic are inconsistent in Westerners of Europe and the United States, and researchers currently appear to recognise that weight cycling may not independently increase but strongly induce the risk of diabetes.[4-8] In contrast, the risk of diabetes in Asian weight cyclers has not been researched.

Due to the preconception linking being slim to an aesthetic standard,[9 10] many Asian women make an effort to lose weight.[11 12] Another group likely to try to reduce weight are middle-aged East Asian businesspeople who have gained weight but have few opportunities for physical activity. Recent studies have demonstrated that East Asians are much more likely than Westerners to develop type 2 diabetes at a lower body mass index (BMI).[13] As a result, it may take only a small change in weight to alter the risk of diabetes for East Asians. In addition, the literature indicates that in Japan, diet, physical activity, prevalence of overweight individuals and aesthetic consciousness of metropolitan residents are different from those of rural residents.[14 15] The aim of this study was to establish whether weight cyclers residing in Japan were at an increased risk of diabetes. We used Japanese urban and rural data to examine this clinical question in populations with varying lifestyles.

METHODS

Study participants and measurements

For peer review only - http://bmjopen.bmj.com/site/about/guidelines.xhtml

In this cohort study, we enrolled participants from an urban area, Tokyo Metropolis and a rural area, Yamanashi Prefecture. In Tokyo, we enrolled employees of private companies who underwent medical check-ups between January 2005 and December 2014 at St. Luke's International Hospital. These annual medical check-ups were based on a legal obligation imposed by the Industrial Safety and Health Act in Japan.[16] In the Yamanashi Prefecture, we enrolled employees and residents who paid for a private comprehensive medical check-up service between April 1999 and March 2009 at the Yamanashi Koseiren Health Care Center. A subset of participants in Yamanashi used a subsidy from their employers or administrative agencies to receive the private medical check-up. Thus, the participants in the urban area received almost annual medical check-ups over 10 years, and those in the rural area received occasional voluntary check-ups. Participants were included in the analysis if they had no diagnosis of diabetes and an HbA1c less than 6.5% (48 mmol/mol) during a baseline period for the first three years of the 10-year period. If they received medical check-ups two or three times during the first three years, the data from the first visit were adopted for the baseline. Those included were also required to undergo a medical check-up at least twice during the latter seven years of the study. Hence, participants needed to receive at least three medical check-ups for the categorisation of weight change patterns (exposure). The onset of diabetes was identified through questionnaires for the diagnosis of diabetes, the commencement of diabetic therapies or glycated haemoglobin (HbA1c) ≥ 6.5% (48 mmol/mol).[17,18] At the institution in Tokyo, trained nurses interviewed the participants from the age of 20 years to establish their changes in weight. BMI was calculated as the participant's weight in kilograms divided by the square of their height in metres.

Weight change categories

For peer review only - http://bmjopen.bmj.com/site/about/guidelines.xhtml

The participants were categorised into five groups according to their patterns of weight change during the 3–10 years since the baseline period. The stable group was comprised of the participants whose weight had not changed from the baseline by more than ±4%. The sustained gain group consisted of the participants who gained more than 4% of their baseline weight and did not subsequently lose this extra weight. Similarly, the sustained loss group was comprised of participants who lost more than 4% of their baseline weight and did not subsequently regain this weight. The gain-loss group included participants who gained more than 4% of their baseline weight but brought their weight back below +4%. The loss-gain group included participants who lost more than 4% of their baseline weight but brought their weight back above −4%. From the last time point of this categorisation, the outcome of incident diabetes was measured. Therefore, the duration of observing whether the participants developed diabetes was between one and six years among the categories. The incidence of diabetes was measured after the participants were categorised, and any data measured after a diagnosis of diabetes were ignored to conserve the temporality of exposure to outcome for an epidemiological causation.[19] The incidence of diabetes was measured after the participants were categorised, and any data measured after a diagnosis of diabetes were ignored to conserve the temporality of exposure to outcome for an epidemiological causation.

The ±4% change in weight used for this categorisation was determined as approximately a one-unit change for a person with a BMI of 22 kg/m^2. This was based on the 2014 reference mean BMIs for Japanese men and women of 23.6 kg/m^2 and 21.7 kg/m^2, respectively.[20] It also took into consideration the relatively short time period of ten years for the observation. The gain-loss and loss-gain group of participants were the weight cyclers of interest.

Statistical analysis

For peer review only - http://bmjopen.bmj.com/site/about/guidelines.xhtml

The baseline characteristics recorded for the participants included age, weight, height, BMI, HbA1c and fasting plasma glucose. We used univariate and multivariate logistic regressions to compare the risk of diabetes between the categorised groups. In the urban data of Tokyo Metropolis, the covariates used for the adjustment at baseline were age, weight change from 20 years of age, BMI, smoking habits, alcohol consumption and physical activity. In the rural data of the Yamanashi Prefecture, the available covariates for the adjustment at baseline were age, BMI, smoking habit and alcohol consumption. The analyses were stratified by sex. In addition, another focus of this study was the impact of weight cycling on incidental diabetes in middle-aged individuals. Hence, for a sensitivity analysis, we restricted the analyses to a middle-aged population of 45 to 64 years. All statistical analyses were performed using SAS statistical software (version 9.3, SAS Institute, NC, USA). The descriptive statistics are reported as the means and standard deviations (SD). All reported p values were two-sided; *P* values of <0.05 were considered to be statistically significant.

RESULTS

Participants

For the multivariate analyses of primary interest, 10,094 men and 10,614 women were enrolled from Tokyo Metropolis (Table 1). The means (SDs) of the baseline characteristics for the men in the urban region were as follows: age 49.6 (11.9) years, weight 68.8 (9.7) kg, BMI 23.7 (2.8) kg/m^2 and HbA1c 5.4% (0.3%) (35.3 [3.7] mmol/mol). For the women in the urban region, these values were as follows: age 48.3 (11.3) years, weight 52.3 (7.4) kg, BMI 21.0 (2.9) kg/m^2 and HbA1c 5.3% (0.4%) (35.0 [3.8] mmol/mol).

Table 1 also shows the baseline characteristics of 4,818 men and 4,852 women enrolled from the Yamanashi Prefecture. The baseline characteristics for the men in the rural region

For peer review only - http://bmjopen.bmj.com/site/about/guidelines.xhtml

were as follows: age 51.2 (10.3) years, weight 65.7 (9.1) kg, BMI 23.2 (2.7) kg/m^2 and HbA1c 5.3% (0.3%) (34.3 [3.8] mmol/L). For the women in the rural area, the values were as follows: age 52.1 (9.4) years, weight 53.1 (7.5) kg, BMI 22.1 (2.9) kg/m^2 and HbA1c 5.3% (0.3%) (34.4 [3.5] mmol/L).

Table 1. Baseline characteristics of the participants from urban (Tokyo Metropolis) and rural (Yamanashi Prefecture) regions of Japan.

Characteristics, mean (standard deviation)	Men	Women
**Urban region**		
No.	10,094	10,614
Age, years	49.6 (11.9)	48.3 (11.3)
Weight, kg	68.8 (9.7)	52.3 (7.4)
Height, cm	170.4 (6.1)	157.7 (5.6)
Body mass index, kg/m^2	23.7 (2.8)	21.0 (2.9)
HbA1c, %	5.4 (0.3)	5.3 (0.4)
(HbA1c, mmol/mol)	35.3 (3.7)	35.0 (3.8)
Fasting plasma glucose, mg/dL	100.8 (8.6)	94.5 (8.0)
(Fasting plasma glucose, mmol/L)	5.6 (0.5)	5.2 (0.4)
**Rural region**		
No.	4,818	4,852
Age, years	51.2 (10.3)	52.1 (9.4)
Weight, kg	65.7 (9.1)	53.1 (7.5)
Height, cm	168.1 (6.2)	154.8 (5.6)
Body mass index, kg/m^2	23.2 (2.7)	22.1 (2.9)
HbA1c, %	5.3 (0.3)	5.3 (0.3)
(HbA1c, mmol/mol)	34.3 (3.8)	34.4 (3.5)
Fasting plasma glucose, mg/dL	96.3 (9.0)	93.2 (8.5)
(Fasting plasma glucose, mmol/L)	5.3 (0.5)	5.2 (0.5)

For peer review only

For peer review only - http://bmjopen.bmj.com/site/about/guidelines.xhtml

Risk of diabetes in urban and rural Japan

Tables 2 and 3 present the incidence of diabetes and the odds ratios (ORs) for each explanatory variable in the urban and rural regions, respectively. For the men in the urban region, the adjusted ORs (95% confidence intervals [95%CIs]) compared to the stable group were 3.07 (2.15–4.39) in the sustained gain group, 0.51 (0.32–0.82) in the gain-loss group, 0.63 (0.45–0.89) in the loss-gain group and 1.11 (0.77–1.59) in the sustained loss group. For the women in the urban region, the adjusted ORs relative to the stable group were 7.00 (4.11–11.94) in the sustained gain group, 1.05 (0.57–1.95) in the gain-loss group, 0.72 (0.39–1.34) in the loss-gain group and 1.48 (0.80–2.74) in the sustained loss group. For the men in the rural region, the adjusted ORs were 3.15 (1.70–5.83) in the sustained gain group, 0.44 (0.15–1.29) in the gain-loss group, 1.58 (0.78–3.17) in the loss-gain group and 0.36 (0.12–1.05) in the sustained loss group. For the women in the rural region, the adjusted ORs were 1.43 (0.59–3.48) in the sustained gain group, 0.77 (0.28–2.14) in the gain-loss group, 0.41 (0.12–1.44) in the loss-gain group and 0.32 (0.09–1.10) in the sustained loss group.

Table 2. The incidence and odds ratios (95% CIs) of diabetes related to patterns of weight change over 10 years in residents of urban Japan

Exposure variables		Acquired DM / No. of subjects (incidence, %)	Crude	Multivariate
**Men (No. For multivariate analysis =10,094)**				
Baseline age	Per 10 years	—	1.44 (1.33–1.55)	1.44 (1.29–1.61)
Weight change from 20 years of age, kg	< −5	7/1438 (0.5)	2.18 (1.14–4.18)	1.68 (0.75–3.80)
	−5 to +5	73/10646 (0.7)	Ref	Ref
	+5 to +10	31/3200 (1.0)	1.98 (1.45–2.72)	1.54 (1.03–2.30)
	> +10	78/2166 (3.6)	3.37 (2.54–4.48)	2.08 (1.40–3.10)
Baseline BMI,	< 18.5	6/387 (1.6)	1.35 (0.57–3.16)	0.88 (0.29–2.70)

For peer review only - http://bmjopen.bmj.com/site/about/guidelines.xhtml

kg/m^2	18.5–22	49/4236 (1.2)	Ref	Ref
	22–25	168/7220 (2.3)	2.04 (1.48–2.89)	1.73 (1.14–2.63)
	> 25	190/4699 (4.0)	3.60 (2.62–4.94)	2.52 (1.60–3.95)
Weight change pattern over 10 years	Sustained loss	66/1903 (3.5)	1.39 (1.03–1.87)	1.11 (0.77–1.59)
	Loss–gain	87/4644 (1.9)	0.74 (0.56–0.97)	0.63 (0.45–0.89)
	Stable	142/5621 (2.5)	Ref	Ref
	Gain–loss	38/3063 (1.2)	0.49 (0.34–0.70)	0.51 (0.32–0.82)
	Sustained gain	80/1311 (6.1)	2.51 (1.89–3.32)	3.07 (2.15–4.39)
Smoking	None	123/6385 (1.9)	Ref	Ref
	Ex-smoker	150/5940 (2.5)	1.32 (1.04–1.68)	0.97 (0.71–1.31)
	Current smoker	140/4217 (3.3)	1.75 (1.37–2.23)	1.73 (1.25–2.34)
Alcohol drinking	None	69/2250 (3.1)	Ref	Ref
	Sometimes	48/1667 (2.9)	0.94 (0.65–1.36)	0.96 (0.66–1.40)
	Usually	156/6177 (2.5)	0.82 (0.61–1.09)	0.78 (0.58–1.05)
Duration of walking per day	Per 30 min	—	0.996 (0.92–1.08)	1.01 (0.92–1.11)
Physical activity	0–1/week	85/3282 (2.6)	Ref	Ref
	1–2/week	108/4105 (2.6)	1.02 (0.76–1.36)	1.02 (0.76–1.36)
	3–5/week	43/1541 (2.8)	1.08 (0.74–1.57)	0.98 (0.67–1.45)
	6–7/week	37/1166 (3.2)	1.23 (0.83–1.82)	1.06 (0.69–1.62)
**Women (No. for multivariate analysis = 10,614)**				
Baseline age	Per 10 years	—	1.66 (1.48–1.87)	1.78 (1.50–2.12)
Weight change from 20 years of age, kg	< −5	11/439 (2.5)	0.71 (0.33–1.54)	0.48 (0.18–1.25)
	−5 to +5	61/5243 (1.2)	Ref	Ref
	+5 to +10	109/4779 (2.3)	1.42 (0.93–2.16)	0.96 (0.55–1.65)
	> +10	232/6081 (3.8)	5.41 (3.92–7.47)	2.10 (1.20–3.67)
Baseline BMI, kg/m^2	< 18.5	20/2916 (0.7)	1.24 (0.74–2.08)	1.47 (0.76–2.86)
	18.5–22	52/9388 (0.6)	Ref	Ref
	22–25	60/3676 (1.6)	2.98 (2.05–4.33)	2.01 (1.21–3.34)
	> 25	57/1470 (3.9)	7.24 (4.95–10.59)	2.91 (1.53–5.52)
Weight change pattern over 10 years	Sustained loss	24/1661 (1.4)	2.11 (1.25–3.54)	1.48 (0.80–2.74)
	Loss–gain	32/4022 (0.7)	1.15 (0.72–1.86)	0.72 (0.39–1.34)
	Stable	36/5212 (1.3)	Ref	Ref

For peer review only

For peer review only - http://bmjopen.bmj.com/site/about/guidelines.xhtml

Yokomichi H et al.

	Gain–loss	30/4630 (0.6)	0.94 (0.58–1.53)	1.05 (0.57–1.95)
	Sustained gain	67/1925 (3.5)	5.19 (3.45–7.80)	7.00 (4.11–11.94)
Smoking	None	156/14,194 (1.1)	Ref	Ref
	Ex-smoker	17/1789 (1.0)	0.86 (0.52–1.1.43)	0.85 (0.45–1.61)
	Current smoker	16/1467 (1.1)	0.99 (0.59–1.67)	1.20 (0.63–2.32)
Alcohol drinking	None	78/5669 (1.4)	Ref	Ref
	Sometimes	20/2035 (1.0)	0.71 (0.43–1.17)	0.91 (0.55–1.52)
	Usually	27/2910 (0.9)	0.67 (0.43–1.04)	0.95 (0.60–1.51)
Duration of walking per day	Per 30 min	—	1.01 (0.93–1.11)	1.01 (0.90–1.13)
Physical activity	0–1/week	37/3894 (1.0)	Ref	Ref
	1–2/week	42/3760 (1.1)	1.18 (0.76–1.84)	1.11 (0.70–1.75)
	3–5/week	27/1874 (1.4)	1.52 (0.93–2.52)	1.14 (0.67–1.94)
	6–7/week	19/1086 (1.7)	1.86 (1.06–3.24)	1.43 (0.79–2.60)

DM, diabetes mellitus; CI, confidence interval; Ref, reference group; BMI, body mass index

Table 3. The incidence and odds ratios (95% CIs) of diabetes related to patterns of weight change over 10 years in residents of rural Japan

Exposure variables		Acquired DM / No. of subjects (incidence, %)	Crude	Multivariate
**Men (No. for multivariate analysis = 4818)**				
Baseline age	Per 10 years	—	1.36 (1.08–1.72)	1.60 (1.24–2.05)
Baseline BMI, kg/m^2	< 18.5	0/167 (0)	—	—
	18.5–22	10/1425 (0.7)	Ref	Ref
	22–25	21/2079 (1.0)	1.44 (0.68–3.08)	1.69 (0.79–3.63)
	> 25	35/1148 (3.1)	4.45 (2.19–9.03)	5.81 (2.82–11.97)
Weight change pattern over 10 years	Sustained loss	4/725 (0.6)	0.43 (0.15–1.25)	0.36 (0.12–1.05)
	Loss–gain	13/681 (1.9)	1.50 (0.75–3.00)	1.58 (0.78–3.17)
	Stable	22/1719 (1.3)	Ref	Ref
	Gain–loss	4/916 (0.4)	0.34 (0.12–0.99)	0.44 (0.15–1.29)
	Sustained gain	23/778 (3.0)	2.35 (1.30–4.24)	3.15 (1.70–5.83)

For peer review only

Smoking	None	23/1695 (1.4)	Ref	Ref
	Ex-smoker	8/998 (0.8)	0.59 (0.26–1.32)	0.72 (0.32–1.64)
	Current smoker	35/2126 (1.7)	1.22 (0.72–2.07)	1.50 (0.85–2.62)
Drinking	None	11/1071 (1.0)	Ref	Ref
	Drinker	55/3748 (1.5)	1.44 (0.75–2.75)	1.52 (0.79–2.94)
**Women (No. for multivariate analysis = 4852)**				
Baseline age	Per 10 years	—	1.43 (0.99–2.06)	1.23 (0.83–1.84)
Baseline BMI, kg/m^2	< 18.5	0/411 (0)	—	—
	18.5–22	7/2135 (0.3)	Ref	Ref
	22–25	14/1563 (0.9)	2.75 (1.11–6.82)	2.69 (1.07–6.75)
	> 25	13/744 (1.8)	5.41 (2.15–13.60)	5.29 (2.07–13.51)
Weight change pattern over 10 years	Sustained loss	3/863 (0.3)	0.37 (0.11–1.29)	0.32 (0.09–1.10)
	Loss–gain	3/757 (0.4)	0.42 (0.12–1.47)	0.41 (0.12–1.44)
	Stable	15/1615 (0.9)	Ref	Ref
	Gain–loss	5/827 (0.6)	0.65 (0.24–1.79)	0.77 (0.28–2.14)
	Sustained gain	8/791 (1.0)	1.09 (0.46–2.58)	1.43 (0.59–3.48)
Smoking	None	2/646 (0.3)	Ref	Ref
	Ex-smoker	29/3822 (0.8)	2.45 (0.58–10.31)	2.19 (0.52–9.26)
	Current smoker	3/375 (0.8)	2.60 (0.43–15.60)	3.33 (0.55–20.28)
Drinking	None	31/3542 (0.9)	Ref	Ref
	Drinker	3/1311 (0.2)	0.26 (0.08–0.85)	0.29 (0.09–0.95)

DM, diabetes mellitus; CI, confidence interval; Ref, reference group; BMI, body mass index

Table 4 shows the risk of diabetes risk due to weight cycling in the middle-aged population. The ORs (95% CIs) for incidental diabetes were 0.57 (0.32–1.01) in the gain-loss group and 0.74 (0.49–1.11) in the loss-gain group among men living in the urban area. The corresponding ORs (95% CIs) were 0.80 (0.36–1.77) and 0.76 (0.37–1.57), respectively among women living in the urban area; 0.58 (0.19–1.77) and 1.76 (0.80–3.87), respectively

For peer review only

For peer review only - http://bmjopen.bmj.com/site/about/guidelines.xhtml

among men living in rural area. The ORs were 0.82 (0.26–2.62) and 0.54 (0.15–1.94), respectively among the women living in the rural area.

Table 4. The incidence and odds ratios of diabetes related to patterns of weight change over 10 years in middle-aged residents (45–64 years) in Japan

Exposure variables		Acquired DM / No. of subjects (incidence, %)	Odds ratio (95% CI)	Adjusted odds ratio (95% CI)
**Urban middle-aged men (No. for multivariate analysis =4,882)**				
Weight change pattern over 10 years	Sustained loss	35/981 (3.6)	1.04 (0.65–1.67)	0.97 (0.60–1.55)
	Loss-gain	62/2203 (2.8)	0.79 (0.53–1.18)	0.74 (0.49–1.11)
	Stable	100/2749 (3.6)	Ref	Ref
	Gain-loss	26/1098 (2.4)	0.56 (0.32–0.999)	0.57 (0.32–1.01)
	Sustained gain	50/489 (10.2)	3.09 (2.00–4.76)	3.13 (2.00–4.89)
**Urban middle-aged women (No. for multivariate analysis = 5,053)**				
Weight change pattern over 10 years	Sustained loss	15/994 (1.5)	1.30 (0.62–2.73)	1.13 (0.53–2.41)
	Loss-gain	20/1992 (1.0)	0.87 (0.43–1.77)	0.76 (0.37–1.57)
	Stable	24/2285 (1.1)	Ref	Ref
	Gain-loss	17/1642 (1.0)	0.74 (0.34–1.62)	0.80 (0.36–1.77)
	Sustained gain	40/603 (6.6)	5.62 (3.05–10.37)	6.97 (3.67–13.25)
**Rural middle-aged men (No. for multivariate analysis =2,937)**				
Weight change pattern over 10 years	Sustained loss	3/447 (0.7)	0.48 (0.14–1.66)	0.42 (0.12–1.47)
	Loss-gain	11/449 (2.5)	1.78 (0.81–3.91)	1.76 (0.80–3.87)
	Stable	15/1078 (1.4)	Ref	Ref
	Gain-loss	4/546 (0.7)	0.52 (0.17–1.58)	0.58 (0.19–1.77)
	Sustained gain	12/417 (2.9)	2.10 (0.98–4.53)	2.48 (1.12–5.49)
**Rural middle-aged women (No. for multivariate analysis = 3,347)**				
Weight change pattern over 10 years	Sustained loss	1/638 (0.2)	0.16 (0.02–1.25)	0.14 (0.02–1.01)
	Loss-gain	3/579 (0.5)	0.54 (0.15–1.92)	0.54 (0.15–1.94)
	Stable	11/1140 (1.0)	Ref	Ref
	Gain-loss	4/558 (0.7)	0.74 (0.24–2.34)	0.82 (0.26–2.62)
	Sustained gain	5/432 (1.2)	1.20 (0.42–3.48)	1.30 (0.44–3.84)

For peer review only

For peer review only - http://bmjopen.bmj.com/site/about/guidelines.xhtml

Multivariate logistic regression calculated odds ratios with an adjustment for baseline age and BMI, weight change from 20 years of age, smoking and drinking habits, duration of walking per day and physical activity per week in the urban data. The baseline age and BMI and smoking and drinking habits in the rural data.

For peer review only

For peer review only - http://bmjopen.bmj.com/site/about/guidelines.xhtml

DISCUSSION

The data for the urban region suggest that the risk of diabetes in male and female weight cyclers was similar to or lower than that of the participants who maintained a stable weight. In particular, the data for Japanese men residing in an urban region indicate that, compared with the men who maintained a stable weight, there was a significantly reduced risk of diabetes associated with weight cycling. The data for Japanese women residing in a rural region also suggest a reduced risk of diabetes with weight cycling compared with maintaining a stable weight. These results are reinforced by a sensitivity analysis with a restriction on middle-aged individuals, representing almost same ORs as those in the entire population except for the OR in the gain-loss group among women residing in the urban region.

These observations are not consistent with those from previous studies of Western populations, which showed that weight cycling significantly or non-significantly increased the risk of diabetes for point estimates. In the Framingham Heart Study, approximately 1 kg/m^2 of weight cycling in middle-aged Americans carried a hazard ratio of 1.1 (95%CI: 0.8–1.5) for the risk of diabetes after adjusting for sex and BMI at 25 years of age.[4] In the American middle-aged women of the National Health and Nutrition Examination Survey, weight cycling of 4.5–9.1 kg and 9.1–22.2 kg with an intentional weight loss three or more times in four years carried ORs for the risk of diabetes of 1.11 (95%CI: 0.89–1.37) and 1.39 (95%CI: 0.90–2.13), respectively.[5] A study from a cohort of medical students at the Johns Hopkins University School of Medicine reported that the highest quartile of BMI variability for ages between 25 and 45 years had an OR of 2.1 (95%CI: 1.0–4.6) for the risk of diabetes after 50 years,

For peer review only - http://bmjopen.bmj.com/site/about/guidelines.xhtml

compared with the other three lower quartiles.[21] In a large German cohort, weight cycling of ≥1.5 kg/year was significantly associated with an adjusted hazard ratio of 1.34.[6]

The inconsistency between the Western studies and this study that weight cycling was not associated with an increase in the risk of diabetes but may even reduce the risk, may be due to ethnic differences in diet,[22] the capacity to gain weight[23] and self-consciousness about body weight.[24] Further research is required to explore why the relationship between weight cycling and risk of diabetes is inversed between Western and East Asian populations. The reason may be attributable to different motivations to lose weight in the context of different diet cultures and body self-consciousness.[25] The East Asians who try to lose weight may be particularly those who are relatively concerned about the poor health outcomes of being overweight. Westerners described in the study cohorts who tried to lose weight may, in an extreme expression, have been those who lost and regained a great deal of weight and potentially ran the risk of poor health outcomes.

From the data from the urban region, a weight increase of more than +5 kg above the participant's weight at the age of 20 years increased the risk of developing diabetes with a dose–response relationship in both men and women (Table 2). Furthermore, in both sexes, an increase in weight of more than +10 kg above that when aged 20 years more than doubles the risk of diabetes, with statistical significance, compared to the risk for those who maintained their weight within ±5 kg of their weight when aged 20 years. These results agree with the study involving a US cohort, which reported a relative risk of 3.2 (95%CI: 1.4–7.4) for the highest quartile of an increase in BMI from 25 to 45

For peer review only - http://bmjopen.bmj.com/site/about/guidelines.xhtml

years of age in comparison with the other three lower quartiles.[21] A dose–response relationship of weight change from that aged 20 years to the risk of diabetes occurred in the Japanese women residing in the urban region, with ORs of 0.48 (95%CI: 0.18–1.25) for a change less than 5 kg, 0.96 (95%CI: 0.55–1.65) for an increase of between 5 and 10 kg and 2.10 (95%CI: 1.20–3.67) for an increase greater than 10 kg. In contrast, the Japanese men who had lost 5 kg or more of body weight between the age of 20 years and early middle age had an OR of 1.68 (95%CI: 0.75–3.80). This paradoxically increased OR was most likely due to the small number of participants (seven) who developed diabetes among a weight loss group of 1438 people.

This study had several limitations. The first of these was the threshold of ±4% weight change in 10 years. This threshold referred to a study from United Kingdom on the association between weight change and the risk of diabetes.[26] The threshold of a ±4% weight change was calculated as approximately 1 BMI unit in Japanese people with a mean BMI of 23 kg/m^2. However, the threshold for categorisation of weight cycling should vary according to mean BMIs in different ethnicities, in which people have different insulin sensitivities.[13 27] Second, we did not evaluate insulin sensitivity. Measuring fasting plasma glucose and insulin concentration to calculate HOMA-IR,[28] an index of insulin resistance, would have allowed us to assess the association between weight cycling and the physiological hazard of diabetes. Third, the weight changes recorded in the rural region may be misclassified due to missing data for the years when participants did not undergo the health examination. However, such misclassification would bias the ORs to the null hypothesis, and we believe that such a bias would not change the conclusions for the rural region. Fourth, we could not examine whether the

For peer review only - http://bmjopen.bmj.com/site/about/guidelines.xhtml

weight cycling was intentional; however, we consider that a subset of unintentional weight loss could be attributed to metabolic diseases, and patients with such diseases could not usually regain the weight within a short duration. Fifth, the urban data for the weight at 20 years of age were derived from the participants' memory. Thus, recall bias could have existed in the results. Sixth, a subset of the diagnoses in this study were not made by physicians but via an epidemiological criteria.[17] However, most observational studies by nature rely on epidemiological criteria for detecting diabetes, and the use of a consistent diagnostic criterion can allow researchers to compare the risk of diabetes onset between the reference group and that of interest. Last, this study lacks the information pertaining to lifestyle, including diet, marriage status, job type and owning a car, that may have partly explain the association between weight cycling and incident diabetes.

The present study also has several strengths. First, to the best of our knowledge, this is the first study to explore the relationship between weight cycling and the risk of diabetes in Asians. Since the relationship in this study was opposite to that of Americans, further research in East Asians is necessary to confirm this relationship. Next, this study was conducted in two differently characterised populations (urban and rural residents). The ORs of the risk of diabetes were 1.05 (95%CI: 0.57–1.95) in the weight gain-loss pattern of the urban resident women and 1.58 (95%CI: 0.78–3.17) in weight loss-gain pattern of the rural resident men. However, all other weight cycling patterns for both sexes in the urban and rural regions were negatively correlated with the risk of diabetes. Third, the number of participants in both sexes were approximately 10,000 in the urban region and approximately 5,000 in the rural region. Since the present study included a

For peer review only - http://bmjopen.bmj.com/site/about/guidelines.xhtml

large number of participants from both urban and rural Japan, Japanese and East Asian weight cyclers can refer to its results.

This study can contribute to aiding public health practitioners and on-site clinical professionals prevent diabetes in the general population. A sustained weight gain greater than 4% over ten years in middle age and more than 5 kg of additional weight gained since the age of 20 years both carried an increased risk of diabetes for both sexes. Since middle-aged people can easily undergo such a small weight gain over the short or long term, non-diabetic people within the normal BMI range should be cautious about the risks resulting from even such a slight weight gain through their lifetime. An interventional study indicated that weight loss (−1.8 kg/m^2 of BMI in a diet intervention group and −3.3 kg/m^2 of BMI in a diet-and-exercise intervention group) improved insulin sensitivity in Japanese patients with obesity and type 2 diabetes.[29] Improved insulin sensitivity was also observed in Americans who maintained their weight with treadmill-based exercise and no alteration in their diet.[30] In addition, studies have demonstrated that building muscle through exercising without changing weight improves insulin sensitivity.[31]

In the present study, two profiles of weight cycling pattern resulted in an increase in the risk of diabetes, whereas the risk decreased in the other six profiles. Since better insulin sensitivity directly leads to a decreased risk of diabetes, the results may be due to the differences in how the participants lost or gained weight (i.e. whether they lost or gained fat or muscle mass). Recent studies have indicated that sarcopenia, the loss of skeletal muscle alone or with increased fat mass in ageing, is a leading cause of death in old age.[32] The results of the present study, in the context of previous studies, suggest

For peer review only - http://bmjopen.bmj.com/site/about/guidelines.xhtml

that over both the short- and long-term, people can reduce their risk of diabetes by losing fat and maintaining muscle mass.

CONCLUSIONS

In men in the urban region and women of rural region in Japan, weight cycling has been associated with a reduction in the risk of diabetes, with statistical significance and non-significance, respectively; however, a clear association was not observed in the women of the urban region and men of the rural region. The results were different than those in Western countries and may be attributed to the motivations of the weight cyclers. In addition, the risk of diabetes increases linearly with weight gain from the age of 20 years in Japanese urban men and women. A study that includes the measurement of insulin sensitivity is necessary to confirm the present results and to improve the understanding of the risks for East Asian weight cyclers.

(3,243 words; limit of 4,000 words)

For peer review only

For peer review only - http://bmjopen.bmj.com/site/about/guidelines.xhtml

Acknowledgments We thank all participants in this study and staff at the St. Luke's International Hospital and the Yamanashi Koseiren Health Care Center for their support, which made this study possible.

Competing interests None declared.

Funding This work was supported by the Ministry of Education, Culture, Sports, Science and Technology of Japan (MEXT) (KAKENHI grant number: JP15K08730 and JP15K15221).

Contributors ZY, YY, MT and OT: setting up the study and data collection. HY, ZY, MM and AT: designing the study. HY and AT: data analysis. HY: writing and revising the draft. ZY, SO, MM, YA and HY: development of the discussion section. All authors read and approved the final manuscript.

Ethics approval The ethics committee of the School of Medicine, University of Yamanashi (approval number: H27-1417).

Data sharing statement No additional data are available.

For peer review only

For peer review only - http://bmjopen.bmj.com/site/about/guidelines.xhtml

REFERENCES

1. Nanri A, Mizoue T, Takahashi Y, et al. Association of weight change in different periods of adulthood with risk of type 2 diabetes in Japanese men and women: the Japan Public Health Center-Based Prospective Study. *J Epidemiol Commun H* 2011;**65**:1104–10.

2. Wannamethee SG, Shaper AG. Weight change and duration of overweight and obesity in the incidence of type 2 diabetes. *Diabetes Care* 1999;**22**:1266–72.

3. Ford ES, Williamson DF, Liu SM. Weight change and diabetes incidence: findings from a national cohort of US adults. *Am J Epidemiol* 1997;**146**:214–22.

4. Waring ME, Eaton CB, Lasater TM, et al. Incident diabetes in relation to weight patterns during middle age. *Am J Epidemiol* 2010;**171**:550–6.

5. Field AE, Manson JE, Laird N, et al. Weight cycling and the risk of developing type 2 diabetes among adult women in the United States. *Obes Res* 2004;**12**:267–74.

6. Neamat-Allah J, Barrdahl M, Hüsing A, et al. Weight cycling and the risk of type 2 diabetes in the EPIC-Germany cohort. *Diabetologia* 2015;**58**:2718–25.

7. French S, Folsom A, Jeffery R, Zheng W, Mink P, Baxter J. Weight variability and incident disease in older women: the Iowa Women's Health Study. *Int J Obes Relat Metab Disord* 1997;**21**:217–23.

8. Morris RD, Rimm AA. Long-term weight fluctuation and non-insulin-dependent diabetes mellitus in white women. *Ann Epidemiol* 1992;**2**:657–64.

9. Mukai T, Crago M, Shisslak CM. Eating attitudes and weight preoccupation among female high school students in Japan. *J Child Psychol Psychiatry* 1994;**35**:677–88.

For peer review only

For peer review only - http://bmjopen.bmj.com/site/about/guidelines.xhtml

10. Swami V, Frederick DA, Aavik T, et al. The attractive female body weight and female body dissatisfaction in 26 countries across 10 world regions: results of the International Body Project I. *Personal Soc Psychol Bull* 2010;**36**:309–25.

11. Chisuwa N, O'Dea JA. Body image and eating disorders amongst Japanese adolescents. a review of the literature. *Appetite* 2010;**54**:5–15.

12. Xu X, Mellor D, Kiehne M, et al. Body dissatisfaction, engagement in body change behaviours and sociocultural influences on body image among Chinese adolescents. *Body Image* 2010;**7**:156–64.

13. Kodama K, Tojjar D, Yamada S, et al. Ethnic differences in the relationship between insulin sensitivity and insulin response: a systematic review and meta-analysis. *Diabetes Care* 2013;**36**:1789–96.

14. Matsushita Y, Yoshiike N, Kaneda F, Yoshita K, Takimoto H. Trends in childhood obesity in Japan over the last 25 years from the national nutrition survey. *Obes Res* 2004;**12**:205–14.

15. Yoshiike N, Seino F, Tajima S, et al. Twenty□year changes in the prevalence of overweight in Japanese adults: The National Nutrition Survey 1976–95. *Obes Rev* 2002;**3**:183–90.

16. Ministry of Health, Labour and Welfare of Japan. Industrial Safety and Health Act (English version). 1972. http://www.japaneselawtranslation.go.jp/law/detail/?printID=&id=1926&re=&vm=02 (acceseed 12 January 2017).

17. American Diabetes Association. Diagnosis and classification of diabetes mellitus. *Diabetes Care* 2010;**33**:S62–S69.

For peer review only

For peer review only - http://bmjopen.bmj.com/site/about/guidelines.xhtml

18. The International Expert Committee. International Expert Committee report on the role of the A1C assay in the diagnosis of diabetes. *Diabetes Care* 2009;**32**:1327–34.

19. Kaufman J, Poole C. Looking back on "causal thinking in the health sciences". *Annu Rev Publ Health* 2000;**21**:101-19.

20. NCD Risk Factor Collaboration. Worldwide trends in diabetes since 1980: a pooled analysis of 751 population-based studies with 4· 4 million participants. *Lancet* 2016;**387**:1513–30.

21. Brancati FL, Wang N, Mead LA, et al. Body weight patterns from 20 to 49 years of age and subsequent risk for diabetes mellitus: the Johns Hopkins Precursors Study. *Arch Int Med* 1999;**159**:957–63.

22. Slocum R. Race in the study of food. *Prog Hum Geogr* 2011;**35**:303–27.

23. The DECODE–DECODA Study Group. Age, body mass index and type 2 diabetes—associations modified by ethnicity. *Diabetologia* 2003;**46**:1063–70.

24. Mintz LB, Kashubeck S. Body image and disordered eating among Asian American and Caucasian college students an examination of race and gender differences. *Psychol Women Q* 1999;**23**:781–96.

25. Frederick DA, Forbes GB, Grigorian KE, et al. The UCLA Body Project I: gender and ethnic differences in self-objectification and body satisfaction among 2,206 undergraduates. *Sex Roles* 2007;**57**:317–27.

26. Wannamethee SG, Shaper AG, Walker M. Overweight and obesity and weight change in middle aged men: impact on cardiovascular disease and diabetes. *J Epidemiol Commun H* 2005;**59**:134–9.

For peer review only

For peer review only - http://bmjopen.bmj.com/site/about/guidelines.xhtml

27. Ntuk UE, Gill JMR, Mackay DF, et al. Ethnic-specific obesity cutoffs for diabetes risk: cross-sectional study of 490,288 UK Biobank participants. *Diabetes Care* 2014;**37**:2500–7.

28. Matthews D, Hosker J, Rudenski A, et al. Homeostasis model assessment: insulin resistance and β-cell function from fasting plasma glucose and insulin concentrations in man. *Diabetologia* 1985;**28**:412–9.

29. Yamanouchi K, Shinozaki T, Chikada K, et al. Daily walking combined with diet therapy is a useful means for obese NIDDM patients not only to reduce body weight but also to improve insulin sensitivity. *Diabetes Care* 1995;**18**:775–8.

30. Duncan GE, Perri MG, Theriaque DW, et al. Exercise training, without weight loss, increases insulin sensitivity and postheparin plasma lipase activity in previously sedentary adults. *Diabetes Care* 2003;**26**:557–62.

31. Nassis GP, Papantakou K, Skenderi K, et al. Aerobic exercise training improves insulin sensitivity without changes in body weight, body fat, adiponectin, and inflammatory markers in overweight and obese girls. *Metabolism* 2005;**54**:1472–9.

32. Fielding RA, Vellas B, Evans WJ, et al. Sarcopenia: an undiagnosed condition in older adults. Current consensus definition: prevalence, etiology, and consequences. International Working Group on Sarcopenia. *J Am Med Dir Assoc* 2011;**12**:249–56.

For peer review only

Figure 1. Scheme of how participants were categorised into the five weight change patterns

For peer review only

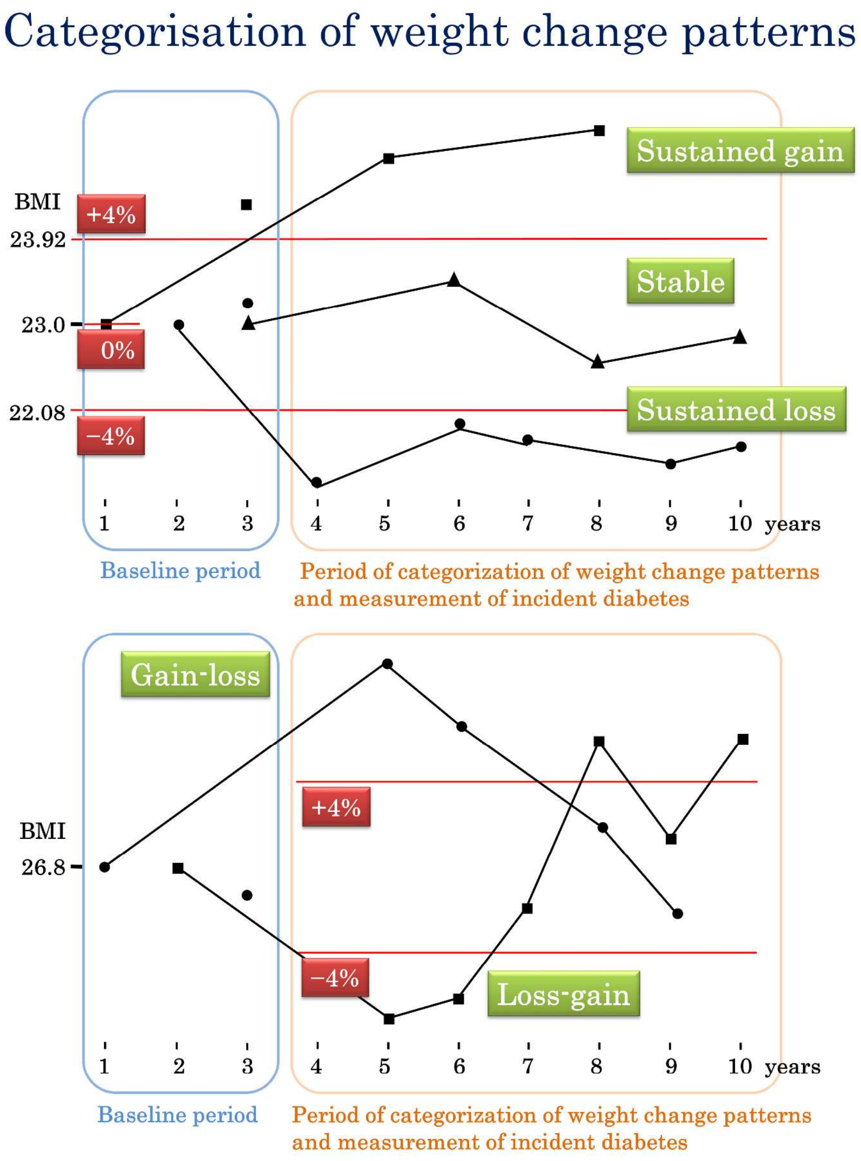

Figure 1
Scheme of how participants wer
339x454mm (300 x 300 DPI)

Supplementary. Baseline characteristics among groups of weight change patterns over 10 years

**Men in urban Japan (n = 10,094)**	Sustained loss	Loss-gain	Stable	Gain-loss	Sustained gain
Age, years	52.3 (12.2)	51.2 (12.0)	50.3 (11.3)	46.0 (11.4)	44.9 (11.3)
Weight, kg	70.8 (10.4)	69.8 (9.5)	68.3 (9.0)	67.3 (9.5)	67.8 (10.8)
Height, cm	170.1 (6.2)	170.3 (6.1)	170.2 (6.0)	170.7 (6.1)	171.2 (6.1)
Body mass index, kg/m2	24.4 (3.0)	24.0 (2.7)	23.5 (2.6)	23.1 (2.8)	23.1 (3.2)
HbA1c, %	5.4 (0.4)	5.4 (0.3)	5.4 (0.3)	5.3 (0.3)	5.3 (0.3)
HbA1c, mmol/mol	35.9 (3.9)	35.7 (3.7)	35.3 (3.7)	34.6 (3.7)	34.6 (3.4)
Fasting plasma glucose, mg/dL	102.3 (9.2)	101.5 (8.8)	101.0 (8.5)	99.0 (8.0)	99.8 (8.4)
Fasting plasma glucose, mmol/L	5.7 (0.5)	5.6 (0.5)	5.6 (0.5)	5.5 (0.4)	5.5 (0.5)
Weight change from 20 years of age, kg	+9.7 (9.1)	+9.0 (8.1)	+8.2 (7.9)	+7.0 (8.4)	+7.0 (20.5)
Current smoking, %	20.0	22.8	23.6	31.3	33.7
Usually drinking alcohol, %	61.7	62.3	63.8	57.3	55.2
Duration of walking per day, min	41.0 (38.3)	41.3 (41.7)	43.3 (41.5)	40.4 (35.2)	38.6 (36.0)
Physical activity of 6–7/week, %	11.2	12.0	11.9	10.4	11.6

**Women in urban Japan (n = 10,614)**	Sustained loss	Loss-gain	Stable	Gain-loss	Sustained gain
Age, years	52.4 (10.7)	51.1 (11.5)	48.9 (11.3)	45.7 (10.6)	43.5 (9.8)
Weight, kg	53.8 (8.0)	53.4 (7.8)	51.7 (7.0)	51.8 (7.1)	51.6 (7.4)
Height, cm	156.9 (5.7)	157.2 (5.9)	157.8 (5.6)	158.1 (5.5)	158.4 (5.3)
Body mass index, kg/m2	21.8 (3.0)	21.6 (3.0)	20.8 (2.7)	20.7 (2.7)	20.6 (2.8)
HbA1c, %	5.5 (0.4)	5.4 (0.4)	5.4 (0.3)	5.3 (0.3)	5.3 (0.3)
HbA1c, mmol/mol	36.1 (4.1)	35.4 (3.8)	35.0 (3.8)	34.4 (3.6)	34.0 (3.7)
Fasting plasma glucose, mg/dL	96.5 (8.9)	95.7 (8.1)	94.6 (8.0)	93.2 (7.3)	92.8 (7.3)

For peer review only

Fasting plasma glucose, mmol/L	5.4 (0.5)	5.3 (0.4)	5.3 (0.4)	5.2 (0.4)	5.2 (1.4)
Weight change from 20 years of age, kg	+4.1 (7.4)	+3.8 (7.3)	+2.4 (7.1)	+2.6 (7.1)	+2.2 (7.5)
Current smoking, %	6.6	6.7	5.7	9.9	11.8
Usually drinking alcohol, %	23.7	26.7	27.3	29.4	28.3
Duration of walking per day, min	42.9 (49.4)	41.2 (49.8)	42.3 (40.9)	41.5 (40.5)	43.8 (51.9)
Physical activity of 6–7/week, %	12.1	10.6	11.3	8.6	8.8
**Men in rural Japan (n = 4,818)**	Sustained loss	Loss-gain	Stable	Gain-loss	Sustained gain
Age, years	53.5 (10.3)	51.7 (9.8)	52.3 (10.1)	49.2 (10.1)	48.3 (10.5)
Weight, kg	67.2 (9.0)	66.2 (8.7)	66.0 (9.1)	64.7 (8.6)	64.3 (9.9)
Height, cm	167.5 (6.2)	168.0 (6.0)	167.8 (6.2)	168.5 (6.2)	168.8 (6.5)
Body mass index, kg/m2	23.9 (2.6)	23.4 (2.6)	23.4 (2.7)	22.8 (2.5)	22.5 (2.9)
HbA1c, %	5.3 (0.4)	5.3 (0.4)	5.3 (0.3)	5.2 (0.3)	5.2 (0.3)
HbA1c, mmol/mol	34.7 (3.9)	34.6 (3.9)	34.3 (3.7)	33.9 (3.8)	33.8 (3.5)
Fasting plasma glucose, mg/dL	97.5 (9.2)	96.4 (9.0)	96.9 (9.1)	95.1 (8.9)	95.1 (8.5)
Fasting plasma glucose, mmol/L	5.4 (0.5)	5.4 (0.5)	5.4 (0.5)	5.3 (0.5)	5.3 (0.5)
Current smoker, %	38.5	42.7	37.7	49.3	58.5
Current drinker, %	79.5	77.5	78.4	75.7	77.5
**Women in rural Japan (n = 4,852)**	Sustained loss	Loss-gain	Stable	Gain-loss	Sustained gain
Age, years	54.9 (9.0)	52.8 (8.6)	53.1 (9.2)	50.7 (9.2)	48.2 (9.9)
Weight, kg	54.0 (8.1)	53.6 (7.5)	52.9 (6.9)	52.6 (7.6)	52.6 (7.8)
Height, cm	154.1 (5.6)	154.8 (5.8)	154.6 (5.4)	155.3 (5.5)	155.7 (5.6)
Body mass index, kg/m2	22.7 (3.1)	22.4 (2.8)	22.1 (2.8)	21.8 (2.9)	21.7 (3.0)
HbA1c, %	5.3 (0.3)	5.3 (0.3)	5.3 (0.3)	5.3 (0.3)	5.2 (0.3)
HbA1c, mmol/mol	34.9 (3.7)	34.6 (3.4)	34.6 (3.6)	34.0 (3.4)	33.6 (3.3)

For peer review only

Yokomichi H et al.

Fasting plasma glucose, mg/dL	94.1 (9.1)	93.0 (8.3)	93.7 (8.8)	93.0 (8.0)	91.9 (7.9)
Fasting plasma glucose, mmol/L	5.2 (0.5)	5.2 (0.5)	5.2 (0.5)	5.2 (0.4)	5.1 (0.4)
Current smoker, %	5.5	8.1	6.6	8.5	11.5
Current drinker, %	24.2	27.2	26.5	27.1	30.9

The data are presented as the mean (SD) or %.

For peer review only

STROBE Statement—checklist of items that should be included in reports of observational studies

	Item No.	Recommendation	Page No.	Relevant text from manuscript
**Title and abstract**	1	(*a*) Indicate the study's design with a commonly used term in the title or the abstract	1	Line 1–2
		(*b*) Provide in the abstract an informative and balanced summary of what was done and what was found	2	Line 23–42
**Introduction**				
Background/rationale	2	Explain the scientific background and rationale for the investigation being reported	5	Line 55–62
Objectives	3	State specific objectives, including any prespecified hypotheses	5	Line 71–73
**Methods**				
Study design	4	Present key elements of study design early in the paper	6	Line 77–78
Setting	5	Describe the setting, locations, and relevant dates, including periods of recruitment, exposure, follow-up, and data collection	6	Line 77–87
Participants	6	(*a*) *Cohort study*—Give the eligibility criteria, and the sources and methods of selection of participants. Describe methods of follow-up<br>*Case-control study*—Give the eligibility criteria, and the sources and methods of case ascertainment and control selection. Give the rationale for the choice of cases and controls<br>*Cross-sectional study*—Give the eligibility criteria, and the sources and methods of selection of participants	6	Line 87–93
		(*b*) *Cohort study*—For matched studies, give matching criteria and number of exposed and unexposed<br>*Case-control study*—For matched studies, give matching criteria and the number of controls per case		NA
Variables	7	Clearly define all outcomes, exposures, predictors, potential confounders, and effect modifiers. Give diagnostic criteria, if applicable	6	Line 93–97
Data sources/ measurement	8*	For each variable of interest, give sources of data and details of methods of assessment (measurement). Describe comparability of assessment methods if there is more than one group	6–7	Line 100–122
Bias	9	Describe any efforts to address potential sources of bias	5	Line 83–90
Study size	10	Explain how the study size was arrived at	8	Line 141–142, 147–148

Continued on next page

For peer review only - http://bmjopen.bmj.com/site/about/guidelines.xhtml

Quantitative variables	11	Explain how quantitative variables were handled in the analyses. If applicable, describe which groupings were chosen and why	6–7	Line 100–122
Statistical methods	12	(*a*) Describe all statistical methods, including those used to control for confounding	7–8	Line 102–103, 124–137
		(*b*) Describe any methods used to examine subgroups and interactions	8	Line 131–133
		(*c*) Explain how missing data were addressed	6	Line 89–93
		(*d*) *Cohort study*—If applicable, explain how loss to follow-up was addressed<br>*Case-control study*—If applicable, explain how matching of cases and controls was addressed<br>*Cross-sectional study*—If applicable, describe analytical methods taking account of sampling strategy		NA
		(*e*) Describe any sensitivity analyses	8	Line 131–133
**Results**				
Participants	13*	(a) Report numbers of individuals at each stage of study—eg numbers potentially eligible, examined for eligibility, confirmed eligible, included in the study, completing follow-up, and analysed	8	Line 141–142, 147–148
		(b) Give reasons for non-participation at each stage	6	Line 92–93
		(c) Consider use of a flow diagram		NA
Descriptive data	14*	(a) Give characteristics of study participants (eg demographic, clinical, social) and information on exposures and potential confounders	9	Table 1
		(b) Indicate number of participants with missing data for each variable of interest		NA
		(c) *Cohort study*—Summarise follow-up time (eg, average and total amount)		NA
Outcome data	15*	*Cohort study*—Report numbers of outcome events or summary measures over time	10–13	Table 2 and 3
		Case-control study—Report numbers in each exposure category, or summary measures of exposure		NA
		Cross-sectional study—Report numbers of outcome events or summary measures		NA
Main results	16	(*a*) Give unadjusted estimates and, if applicable, confounder-adjusted estimates and their precision (eg, 95% confidence interval). Make clear which confounders were adjusted for and why they were included	10–13	Table 2 and 3
		(*b*) Report category boundaries when continuous variables were categorized	10–13 (BMI)	Table 2 and 3
		(*c*) If relevant, consider translating estimates of relative risk into absolute risk for a		

For peer review only - http://bmjopen.bmj.com/site/about/guidelines.xhtml

		meaningful time period		NA
Continued on next page Other analyses	17	Report other analyses done—eg analyses of subgroups and interactions, and sensitivity analyses	14–15	Table 4
**Discussion**				
Key results	18	Summarise key results with reference to study objectives	16	Line 192–201
Limitations	19	Discuss limitations of the study, taking into account sources of potential bias or imprecision. Discuss both direction and magnitude of any potential bias	18–19	Line 246–271
Interpretation	20	Give a cautious overall interpretation of results considering objectives, limitations, multiplicity of analyses, results from similar studies, and other relevant evidence	20	Line 285–298
Generalisability	21	Discuss the generalisability (external validity) of the study results	19–20	Line 281–284
**Other information**				
Funding	22	Give the source of funding and the role of the funders for the present study and, if applicable, for the original study on which the present article is based	22	Line 324–326

*Give information separately for cases and controls in case-control studies and, if applicable, for exposed and unexposed groups in cohort and cross-sectional studies.

Note: An Explanation and Elaboration article discusses each checklist item and gives methodological background and published examples of transparent reporting. The STROBE checklist is best used in conjunction with this article (freely available on the Web sites of PLoS Medicine at http://www.plosmedicine.org/, Annals of Internal Medicine at http://www.annals.org/, and Epidemiology at http://www.epidem.com/). Information on the STROBE Initiative is available at www.strobe-statement.org.

For peer review only - http://bmjopen.bmj.com/site/about/guidelines.xhtml

Weight cycling and the subsequent onset of type 2 diabetes mellitus: ten-year cohort studies in urban and rural Japan

Journal:	*BMJ Open*
Manuscript ID	bmjopen-2016-014684.R2
Article Type:	Research
Date Submitted by the Author:	24-Feb-2017
Complete List of Authors:	Yokomichi, Hiroshi; University of Yamanashi, Department of Health Sciences<br>Ohde, Sachiko; St. Luke's International University, Center for Clinical Epidemiology<br>Takahashi, Osamu; St. Luke's International University, Center for Clinical Epidemiology<br>Mochizuki, Mie; University of Yamanashi, Department of Pediatrics<br>Takahashi, Atsunori; University of Yamanashi, Department of Health Sciences<br>Yoda, Yoshioki; Yamanashi Koseiren Health Care Center<br>Tsuji, Masahiro; Yamanashi Koseiren Health Care Center<br>Akiyama, Yuka; University of Yamanashi, Department of Health Sciences<br>Yamagata, Zentaro; The University of Yamanashi, Interdisciplinary Graduate School of Medicine and Engineering
**Primary Subject Heading**:	Epidemiology
Secondary Subject Heading:	Diabetes and endocrinology, Health services research, Nutrition and metabolism, Sports and exercise medicine
Keywords:	body weight changes, type 2 diabetes, body mass index, Asian, sarcopenia

SCHOLARONE™
Manuscripts

Weight cycling and the subsequent onset of type 2 diabetes mellitus: ten-year cohort studies in urban and rural Japan

Hiroshi Yokomichi[1*], Sachiko Ohde[2], Osamu Takahashi[2], Mie Mochizuki[3], Atsunori Takahashi[1], Yoshioki Yoda[4], Masahiro Tsuji[4], Yuka Akiyama[1] and Zentaro Yamagata[1]

[1]Department of Health Sciences, University of Yamanashi, Yamanashi, Japan

[2]Center for Clinical Epidemiology, St. Luke's International University, Tokyo, Japan

[3]Department of Pediatrics, University of Yamanashi, Yamanashi, Japan

[4]Yamanashi Koseiren Health Care Center, Yamanashi, Japan

[*]Corresponding author:

Hiroshi Yokomichi

1110 Shimokato, Chuo City, Yamanashi, 4093898, Japan

E-mail: hyokomichi@yamanashi.ac.jp

Tel: +81-(0)80-5524-7303

Keywords: body weight changes; type 2 diabetes; body mass index; Asian; sarcopenia

Word count: 3,508

For peer review only - http://bmjopen.bmj.com/site/about/guidelines.xhtml

ABSTRACT

Objective: To investigate how weight cycling impacts on the risk of diabetes.

Design: Cohort studies.

Setting: Primary healthcare in urban and rural Japan.

Participants: 20,708 urban and 9,670 rural residents.

Primary outcome measures: Odds ratios (ORs) for diabetes in weight loss, loss-gain, stable, gain-loss and weight gain for 10 years. Weight gain and loss were defined as a greater than ±4% change from baseline weight.

Results: In the urban region, the ORs relative to the stable group for the loss-gain and gain-loss groups were 0.63 (95% CI: 0.45–0.89) and 0.51 (95% CI: 0.32–0.82) for men and 0.72 (95% CI: 0.39–1.34) and 1.05 (95% CI: 0.57–1.95) for women, respectively. In the rural region, they were 1.58 (95% CI: 0.78–3.17) and 0.44 (95% CI: 0.15–1.29) in men and 0.41 (95% CI: 0.12–1.44) and 0.77 (95% CI: 0.28–2.14) in women, respectively. The ORs for an increase in weight between 5–10 kg from the age of 20 years were 1.54 (95% CI: 1.03–2.30) in men and 0.96 (95% CI: 0.55–1.65) in women.

Conclusions: In Japan, weight cycling has been associated with a significant reduction in the risk of diabetes for men from an urban region. However, the associations were unclear for women from the urban region and for men and women from the rural region. These results differ from those in Western studies, likely due to differences in diet, insulin secretion and sensitivity and weight-consciousness.

For peer review only

Word count: 242 words (limit: 300 words)

For peer review only

For peer review only - http://bmjopen.bmj.com/site/about/guidelines.xhtml

Strengths and limitations of this study

- Of men in urban Japan, weight cyclers have a lower risk of diabetes than those who maintain a constant body weight.
- In urban and rural Japan, sustained weight gain increases the risk of diabetes.
- There is a dose–response relationship for both sexes between weight gain since the age of 20 years and the risk of diabetes.
- Odds ratios may change according to the threshold of ±4% weight change in 10 years.
- The levels of insulin secretion and sensitivity were not measured.

For peer review only

For peer review only - http://bmjopen.bmj.com/site/about/guidelines.xhtml

INTRODUCTION

Weight gain is a well-known risk factor for incidental type 2 diabetes. Research involving people of Western, Oriental and African descent has quantitatively established the risks of developing diabetes in relation to weight gain.[1-3] Researchers have also raised the question of whether repeatedly gaining and losing weight (weight cycling) is an independent risk factor for developing diabetes due to gaining weight. Studies on this topic are inconsistent in Westerners of Europe and the United States; several prospective studies point to weight cycling as a risk factor for type 2 diabetes while others do not. [4-8] In contrast, the risk of diabetes in Asian weight cyclers has not been researched.

Due to the preconception linking being slim to an aesthetic standard,[9 10] many Asian women make an effort to lose weight.[11 12] Another group likely to try to reduce weight is middle-aged Asian businesspeople who have gained weight but have few opportunities for physical activity. Recent studies have demonstrated that East Asians are much more likely than Westerners to develop type 2 diabetes at a lower body mass index (BMI).[13] As a result, it may take only a small change in weight to alter the risk of diabetes for East Asians. In addition, the literature indicates that in Japan, diet, physical activity, prevalence of overweight individuals and aesthetic consciousness of metropolitan residents are different from those of rural residents.[14 15] The aim of this study was to establish whether weight cyclers residing in Japan were at an increased risk of diabetes. We used Japanese urban and rural data to examine this clinical question in populations with varying lifestyles.

METHODS

Study participants and measurements

For peer review only

In these cohort studies, we enrolled participants from an urban area, Tokyo Metropolis and a rural area, Yamanashi Prefecture. In Tokyo, we enrolled employees of private companies who underwent medical check-ups between January 2005 and December 2014 at St. Luke's International Hospital. These annual medical check-ups were based on a legal obligation imposed by the Industrial Safety and Health Act in Japan.[16] In the Yamanashi Prefecture, we enrolled employees and residents who paid for a private comprehensive medical check-up service between April 1999 and March 2009 at the Yamanashi Koseiren Health Care Center. A subset of participants in Yamanashi used a subsidy from their employers or administrative agencies to receive the private medical check-up. Thus, the participants in the urban area received almost annual medical check-ups over 10 years, and those in the rural area received occasional voluntary check-ups. Participants were included in the analysis if they had no diagnosis of diabetes and an HbA1c less than 6.5% (48 mmol/mol) during a baseline period for the first three years of the 10-year period. If they received medical check-ups two or three times during the first three years, the data from the first visit were adopted for the baseline. Those included were also required to undergo a medical check-up at least twice during the latter seven years of the study. Hence, participants received three to eight medical check-ups to categorise weight change patterns (exposure). The onset of diabetes was identified through questionnaires for the diagnosis of diabetes, the commencement of diabetic therapies or glycated haemoglobin (HbA1c) ≥ 6.5% (48 mmol/mol).[17,18] At the institution in Tokyo, trained nurses interviewed the participants from the age of 20 years to establish their changes in weight. BMI was calculated as the participant's weight in kilograms divided by the square of their height in metres.

Weight change categories

For peer review only - http://bmjopen.bmj.com/site/about/guidelines.xhtml

The participants were categorised into five groups according to their patterns of weight change during the 3–10 years since the baseline period (figure 1). The stable group was comprised of the participants whose weight had not changed from the baseline by more than ±4%. The sustained gain group consisted of the participants who gained more than 4% of their baseline weight and did not subsequently lose this extra weight. Similarly, the sustained loss group was comprised of participants who lost more than 4% of their baseline weight and did not subsequently regain this weight. The gain-loss group included participants who gained more than 4% of their baseline weight but brought their weight back below +4%. The loss-gain group included participants who lost more than 4% of their baseline weight but brought their weight back above −4%. From the last time point of this categorisation, the outcome of incident diabetes was measured. Therefore, the duration of observing whether the participants developed diabetes was between one and six years among the categories. The incidence of diabetes was measured after the participants were categorised, and any data measured after a diagnosis of diabetes were ignored to conserve the temporality of exposure to outcome for an epidemiological causation.[19]

The ±4% change in weight used for this categorisation was determined as approximately a one-unit change for a person with a BMI of 22 kg/m^2. This was based on the 2014 reference mean BMIs for Japanese men and women of 23.6 kg/m^2 and 21.7 kg/m^2, respectively.[20] We also took into consideration the relatively short time period of ten years for the observation. The gain-loss and loss-gain group of participants were the weight cyclers of interest.

Statistical analysis

The baseline characteristics recorded for the participants included age, weight, height, BMI, HbA1c and fasting plasma glucose. We used univariate and multivariable logistic regressions

For peer review only - http://bmjopen.bmj.com/site/about/guidelines.xhtml

to compare the risk of diabetes between the categorised groups. In the urban data of Tokyo Metropolis, the covariates used for the adjustment at baseline were age, weight change from 20 years of age, BMI, smoking habits, alcohol consumption and physical activity. In the rural data of the Yamanashi Prefecture, the available covariates for the adjustment at baseline were age, BMI, smoking habit and alcohol consumption. The analyses were stratified by sex. In addition, another focus of this study was the impact of weight cycling on incidental diabetes in middle-aged individuals. Hence, for a sensitivity analysis, we restricted the analyses to a middle-aged population of 45 to 64 years. All statistical analyses were performed using SAS statistical software (version 9.3, SAS Institute, NC, USA). The descriptive statistics are reported as the means and standard deviations (SD). All reported p values were two-sided; *P* values of <0.05 were considered to be statistically significant.

RESULTS

Participants

For the multivariable analyses of primary interest, 10,094 men and 10,614 women were enrolled from Tokyo Metropolis (Table 1). The means (SDs) of the baseline characteristics for the men in the urban region were as follows: age 49.6 (11.9) years, weight 68.8 (9.7) kg, BMI 23.7 (2.8) kg/m^2 and HbA1c 5.4% (0.3%) (35.3 [3.7] mmol/mol). For the women in the urban region, these values were as follows: age 48.3 (11.3) years, weight 52.3 (7.4) kg, BMI 21.0 (2.9) kg/m^2 and HbA1c 5.3% (0.4%) (35.0 [3.8] mmol/mol).

Table 1 also shows the baseline characteristics of 4,818 men and 4,852 women enrolled from the Yamanashi Prefecture. The baseline characteristics for the men in the rural region were as follows: age 51.2 (10.3) years, weight 65.7 (9.1) kg, BMI 23.2 (2.7) kg/m^2 and HbA1c 5.3% (0.3%) (34.3 [3.8] mmol/L). For the women in the rural area, the values were as

For peer review only

follows: age 52.1 (9.4) years, weight 53.1 (7.5) kg, BMI 22.1 (2.9) kg/m^2 and HbA1c 5.3% (0.3%) (34.4 [3.5] mmol/L).

Table 1. Baseline characteristics of the participants from urban (Tokyo Metropolis) and rural (Yamanashi Prefecture) regions of Japan.

Characteristics, mean (standard deviation)	Men	Women
**Urban region**		
No.	10,094	10,614
Age, years	49.6 (11.9)	48.3 (11.3)
Weight, kg	68.8 (9.7)	52.3 (7.4)
Height, cm	170.4 (6.1)	157.7 (5.6)
Body mass index, kg/m^2	23.7 (2.8)	21.0 (2.9)
HbA1c, %	5.4 (0.3)	5.3 (0.4)
(HbA1c, mmol/mol)	35.3 (3.7)	35.0 (3.8)
Fasting plasma glucose, mg/dL	100.8 (8.6)	94.5 (8.0)
(Fasting plasma glucose, mmol/L)	5.6 (0.5)	5.2 (0.4)
**Rural region**		
No.	4,818	4,852
Age, years	51.2 (10.3)	52.1 (9.4)
Weight, kg	65.7 (9.1)	53.1 (7.5)
Height, cm	168.1 (6.2)	154.8 (5.6)
Body mass index, kg/m^2	23.2 (2.7)	22.1 (2.9)
HbA1c, %	5.3 (0.3)	5.3 (0.3)
(HbA1c, mmol/mol)	34.3 (3.8)	34.4 (3.5)
Fasting plasma glucose, mg/dL	96.3 (9.0)	93.2 (8.5)
(Fasting plasma glucose, mmol/L)	5.3 (0.5)	5.2 (0.5)

For peer review only

Risk of diabetes in urban and rural Japan

For peer review only - http://bmjopen.bmj.com/site/about/guidelines.xhtml

Tables 2 and 3 present the incidence of diabetes and the odds ratios (ORs) for each explanatory variable in the urban and rural regions, respectively. For men in the urban region, means (SDs) of follow-up duration and numbers of measurements were 7.4 (1.9) years and 5.4 (1.6) in the sustained gain group, 8.2 (1.2) years and 5.7 (1.4) in the gain-loss group, 7.9 (1.7) years and 4.9 (1.7) in the stable group, 8.2 (1.2) years and 5.8 (1.4) in the loss-gain group and 7.7 (1.9) years and 5.8 (1.6) in the sustained loss group, respectively. The corresponding values for women in the urban region were 7.5 (1.9) years and 5.6 (1.6) in the sustained gain group, 8.2 (1.1) years and 5.9 (1.4) in the gain-loss group, 7.9 (1.6) years and 5.0 (1.7) in the stable group, 8.2 (1.2) years and 5.9 (1.4) in the loss-gain group and 7.9 (1.7) years and 6.0 (1.5) in the sustained loss group, respectively. For men in the rural region, means (SDs) of follow-up duration and numbers of measurement were 7.4 (1.6) years and 5.4 (1.6) in the sustained gain group, 7.8 (1.4) years and 6.1 (1.3) in the gain-loss group, 7.1 (1.9) years and 5.2 (1.6) in the stable group, 7.9 (1.4) years and 6.2 (1.2) in the loss-gain group and 7.3 (1.7) years and 5.2 (1.6) in the sustained loss group, respectively. The corresponding values for women in the rural region were 7.3 (1.6) years and 5.1 (1.6) in the sustained gain group, 7.9 (1.4) years and 6.0 (1.3) in the gain-loss group, 7.0 (1.8) years and 5.0 (1.6) in the stable group, 7.9 (1.3) years and 6.1 (1.3) in the loss-gain group and 7.4 (1.7) years and 5.4 (1.5) in the sustained loss group, respectively. For the men in the urban region, the adjusted ORs (95% confidence intervals [95%CIs]) compared to the stable group were 3.07 (2.15–4.39) in the sustained gain group, 0.51 (0.32–0.82) in the gain-loss group, 0.63 (0.45–0.89) in the loss-gain group and 1.11 (0.77–1.59) in the sustained loss group. For the women in the urban region, the adjusted ORs compared to the stable group were 7.00 (4.11–11.94) in the sustained gain group, 1.05 (0.57–1.95) in the gain-loss group, 0.72 (0.39–1.34) in the loss-gain group and 1.48 (0.80–2.74) in the sustained loss group. For the men in the rural region, the adjusted ORs were 3.15 (1.70–5.83) in the sustained gain group, 0.44 (0.15–1.29)

For peer review only - http://bmjopen.bmj.com/site/about/guidelines.xhtml

in the gain-loss group, 1.58 (0.78–3.17) in the loss-gain group and 0.36 (0.12–1.05) in the sustained loss group. For the women in the rural region, the adjusted ORs were 1.43 (0.59–3.48) in the sustained gain group, 0.77 (0.28–2.14) in the gain-loss group, 0.41 (0.12–1.44) in the loss-gain group and 0.32 (0.09–1.10) in the sustained loss group.

Table 2. The incidence and odds ratios (95% CIs) of diabetes related to patterns of weight change over 10 years in residents of urban Japan

For peer review only

Exposure variables		Acquired DM / No. of subjects (incidence, %)	Crude	Multivariate
**Men (No. For multivariate analysis =10,094)**				
Baseline age	Per 10 years	—	1.44 (1.33–1.55)	1.44 (1.29–1.61)
Weight change from 20 years of age, kg	< −5	7/1438 (0.5)	2.18 (1.14–4.18)	1.68 (0.75–3.80)
	−5 to +5	73/10646 (0.7)	Ref	Ref
	+5 to +10	31/3200 (1.0)	1.98 (1.45–2.72)	1.54 (1.03–2.30)
	> +10	78/2166 (3.6)	3.37 (2.54–4.48)	2.08 (1.40–3.10)
Baseline BMI, kg/m^2	< 18.5	6/387 (1.6)	1.35 (0.57–3.16)	0.88 (0.29–2.70)
	18.5–22	49/4236 (1.2)	Ref	Ref
	22–25	168/7220 (2.3)	2.04 (1.48–2.89)	1.73 (1.14–2.63)
	> 25	190/4699 (4.0)	3.60 (2.62–4.94)	2.52 (1.60–3.95)
Weight change pattern over 10 years	Sustained loss	66/1903 (3.5)	1.39 (1.03–1.87)	1.11 (0.77–1.59)
	Loss–gain	87/4644 (1.9)	0.74 (0.56–0.97)	0.63 (0.45–0.89)
	Stable	142/5621 (2.5)	Ref	Ref
	Gain–loss	38/3063 (1.2)	0.49 (0.34–0.70)	0.51 (0.32–0.82)
	Sustained gain	80/1311 (6.1)	2.51 (1.89–3.32)	3.07 (2.15–4.39)
Smoking	None	123/6385 (1.9)	Ref	Ref
	Ex-smoker	150/5940 (2.5)	1.32 (1.04–1.68)	0.97 (0.71–1.31)
	Current smoker	140/4217 (3.3)	1.75 (1.37–2.23)	1.73 (1.25–2.34)
Alcohol drinking	None	69/2250 (3.1)	Ref	Ref
	Sometimes	48/1667 (2.9)	0.94 (0.65–1.36)	0.96 (0.66–1.40)
	Usually	156/6177 (2.5)	0.82 (0.61–1.09)	0.78 (0.58–1.05)

Duration of walking per day	Per 30 min	—	0.996 (0.92–1.08)	1.01 (0.92–1.11)
Physical activity	0–1/week	85/3282 (2.6)	Ref	Ref
	1–2/week	108/4105 (2.6)	1.02 (0.76–1.36)	1.02 (0.76–1.36)
	3–5/week	43/1541 (2.8)	1.08 (0.74–1.57)	0.98 (0.67–1.45)
	6–7/week	37/1166 (3.2)	1.23 (0.83–1.82)	1.06 (0.69–1.62)
**Women (No. for multivariate analysis = 10,614)**				
Baseline age	Per 10 years	—	1.66 (1.48–1.87)	1.78 (1.50–2.12)
Weight change from 20 years of age, kg	< −5	11/439 (2.5)	0.71 (0.33–1.54)	0.48 (0.18–1.25)
	−5 to +5	61/5243 (1.2)	Ref	Ref
	+5 to +10	109/4779 (2.3)	1.42 (0.93–2.16)	0.96 (0.55–1.65)
	> +10	232/6081 (3.8)	5.41 (3.92–7.47)	2.10 (1.20–3.67)
Baseline BMI, kg/m^2	< 18.5	20/2916 (0.7)	1.24 (0.74–2.08)	1.47 (0.76–2.86)
	18.5–22	52/9388 (0.6)	Ref	Ref
	22–25	60/3676 (1.6)	2.98 (2.05–4.33)	2.01 (1.21–3.34)
	> 25	57/1470 (3.9)	7.24 (4.95–10.59)	2.91 (1.53–5.52)
Weight change pattern over 10 years	Sustained loss	24/1661 (1.4)	2.11 (1.25–3.54)	1.48 (0.80–2.74)
	Loss–gain	32/4022 (0.7)	1.15 (0.72–1.86)	0.72 (0.39–1.34)
	Stable	36/5212 (1.3)	Ref	Ref
	Gain–loss	30/4630 (0.6)	0.94 (0.58–1.53)	1.05 (0.57–1.95)
	Sustained gain	67/1925 (3.5)	5.19 (3.45–7.80)	7.00 (4.11–11.94)
Smoking	None	156/14,194 (1.1)	Ref	Ref
	Ex-smoker	17/1789 (1.0)	0.86 (0.52–1.1.43)	0.85 (0.45–1.61)
	Current smoker	16/1467 (1.1)	0.99 (0.59–1.67)	1.20 (0.63–2.32)
Alcohol drinking	None	78/5669 (1.4)	Ref	Ref
	Sometimes	20/2035 (1.0)	0.71 (0.43–1.17)	0.91 (0.55–1.52)
	Usually	27/2910 (0.9)	0.67 (0.43–1.04)	0.95 (0.60–1.51)
Duration of walking per day	Per 30 min	—	1.01 (0.93–1.11)	1.01 (0.90–1.13)
Physical activity	0–1/week	37/3894 (1.0)	Ref	Ref
	1–2/week	42/3760 (1.1)	1.18 (0.76–1.84)	1.11 (0.70–1.75)
	3–5/week	27/1874 (1.4)	1.52 (0.93–2.52)	1.14 (0.67–1.94)
	6–7/week	19/1086 (1.7)	1.86 (1.06–3.24)	1.43 (0.79–2.60)

For peer review only - http://bmjopen.bmj.com/site/about/guidelines.xhtml

DM, diabetes mellitus; CI, confidence interval; Ref, reference group; BMI, body mass index

Table 3. The incidence and odds ratios (95% CIs) of diabetes related to patterns of weight change over 10 years in residents of rural Japan

Exposure variables		Acquired DM / No. of subjects (incidence, %)	Crude	Multivariate
**Men (No. for multivariate analysis = 4818)**				
Baseline age	Per 10 years	—	1.36 (1.08–1.72)	1.60 (1.24–2.05)
Baseline BMI, kg/m^2	< 18.5	0/167 (0)	—	—
	18.5–22	10/1425 (0.7)	Ref	Ref
	22–25	21/2079 (1.0)	1.44 (0.68–3.08)	1.69 (0.79–3.63)
	> 25	35/1148 (3.1)	4.45 (2.19–9.03)	5.81 (2.82–11.97)
Weight change pattern over 10 years	Sustained loss	4/725 (0.6)	0.43 (0.15–1.25)	0.36 (0.12–1.05)
	Loss–gain	13/681 (1.9)	1.50 (0.75–3.00)	1.58 (0.78–3.17)
	Stable	22/1719 (1.3)	Ref	Ref
	Gain–loss	4/916 (0.4)	0.34 (0.12–0.99)	0.44 (0.15–1.29)
	Sustained gain	23/778 (3.0)	2.35 (1.30–4.24)	3.15 (1.70–5.83)
Smoking	None	23/1695 (1.4)	Ref	Ref
	Ex-smoker	8/998 (0.8)	0.59 (0.26–1.32)	0.72 (0.32–1.64)
	Current smoker	35/2126 (1.7)	1.22 (0.72–2.07)	1.50 (0.85–2.62)
Drinking	None	11/1071 (1.0)	Ref	Ref
	Drinker	55/3748 (1.5)	1.44 (0.75–2.75)	1.52 (0.79–2.94)
**Women (No. for multivariate analysis = 4852)**				
Baseline age	Per 10 years	—	1.43 (0.99–2.06)	1.23 (0.83–1.84)
Baseline BMI, kg/m^2	< 18.5	0/411 (0)	—	—
	18.5–22	7/2135 (0.3)	Ref	Ref
	22–25	14/1563 (0.9)	2.75 (1.11–6.82)	2.69 (1.07–6.75)
	> 25	13/744 (1.8)	5.41 (2.15–13.60)	5.29 (2.07–13.51)
Weight change pattern over 10 years	Sustained loss	3/863 (0.3)	0.37 (0.11–1.29)	0.32 (0.09–1.10)
	Loss–gain	3/757 (0.4)	0.42 (0.12–1.47)	0.41 (0.12–1.44)
	Stable	15/1615 (0.9)	Ref	Ref
	Gain–loss	5/827 (0.6)	0.65 (0.24–1.79)	0.77 (0.28–2.14)

For peer review only

For peer review only - http://bmjopen.bmj.com/site/about/guidelines.xhtml

	Sustained gain	8/791 (1.0)	1.09 (0.46–2.58)	1.43 (0.59–3.48)
Smoking	None	2/646 (0.3)	Ref	Ref
	Ex-smoker	29/3822 (0.8)	2.45 (0.58–10.31)	2.19 (0.52–9.26)
	Current smoker	3/375 (0.8)	2.60 (0.43–15.60)	3.33 (0.55–20.28)
Drinking	None	31/3542 (0.9)	Ref	Ref
	Drinker	3/1311 (0.2)	0.26 (0.08–0.85)	0.29 (0.09–0.95)

DM, diabetes mellitus; CI, confidence interval; Ref, reference group; BMI, body mass index

Table 4 shows the risk of diabetes risk due to weight cycling in the middle-aged population. The ORs (95% CIs) for incidental diabetes were 0.57 (0.32–1.01) in the gain-loss group and 0.74 (0.49–1.11) in the loss-gain group among men living in the urban area. The corresponding ORs (95% CIs) were 0.80 (0.36–1.77) and 0.76 (0.37–1.57), respectively among women living in the urban area. The ORs were 0.58 (0.19–1.77) and 1.76 (0.80–3.87), respectively among men living in rural area; 0.82 (0.26–2.62) and 0.54 (0.15–1.94), respectively among the women living in the rural area.

Table 4. The incidence and odds ratios of diabetes related to patterns of weight change over 10 years in middle-aged residents (45–64 years) in Japan

Exposure variables		Acquired DM / No. of subjects (incidence, %)	Odds ratio (95% CI)	Adjusted odds ratio (95% CI)
**Urban middle-aged men (No. for multivariate analysis =4,882)**				
Weight change pattern over 10 years	Sustained loss	35/981 (3.6)	1.04 (0.65–1.67)	0.97 (0.60–1.55)
	Loss-gain	62/2203 (2.8)	0.79 (0.53–1.18)	0.74 (0.49–1.11)
	Stable	100/2749 (3.6)	Ref	Ref
	Gain-loss	26/1098 (2.4)	0.56 (0.32–0.999)	0.57 (0.32–1.01)
	Sustained gain	50/489 (10.2)	3.09 (2.00–4.76)	3.13 (2.00–4.89)
**Urban middle-aged women (No. for multivariate analysis = 5,053)**				

For peer review only - http://bmjopen.bmj.com/site/about/guidelines.xhtml

Weight change pattern over 10 years	Sustained loss	15/994 (1.5)	1.30 (0.62–2.73)	1.13 (0.53–2.41)
	Loss-gain	20/1992 (1.0)	0.87 (0.43–1.77)	0.76 (0.37–1.57)
	Stable	24/2285 (1.1)	Ref	Ref
	Gain-loss	17/1642 (1.0)	0.74 (0.34–1.62)	0.80 (0.36–1.77)
	Sustained gain	40/603 (6.6)	5.62 (3.05–10.37)	6.97 (3.67–13.25)
**Rural middle-aged men (No. for multivariate analysis =2,937)**				
Weight change pattern over 10 years	Sustained loss	3/447 (0.7)	0.48 (0.14–1.66)	0.42 (0.12–1.47)
	Loss-gain	11/449 (2.5)	1.78 (0.81–3.91)	1.76 (0.80–3.87)
	Stable	15/1078 (1.4)	Ref	Ref
	Gain-loss	4/546 (0.7)	0.52 (0.17–1.58)	0.58 (0.19–1.77)
	Sustained gain	12/417 (2.9)	2.10 (0.98–4.53)	2.48 (1.12–5.49)
**Rural middle-aged women (No. for multivariate analysis = 3,347)**				
Weight change pattern over 10 years	Sustained loss	1/638 (0.2)	0.16 (0.02–1.25)	0.14 (0.02–1.01)
	Loss-gain	3/579 (0.5)	0.54 (0.15–1.92)	0.54 (0.15–1.94)
	Stable	11/1140 (1.0)	Ref	Ref
	Gain-loss	4/558 (0.7)	0.74 (0.24–2.34)	0.82 (0.26–2.62)
	Sustained gain	5/432 (1.2)	1.20 (0.42–3.48)	1.30 (0.44–3.84)

Multivariable logistic regression calculated odds ratios with an adjustment for baseline age and BMI, weight change from 20 years of age, smoking and drinking habits, duration of walking per day and physical activity per week in the urban data. The baseline age and BMI and smoking and drinking habits in the rural data.

For peer review only

DISCUSSION

The data for the Japanese urban region suggest that the risk of diabetes in male weight cyclers was significantly lower than that of those who maintained a stable weight (table 2). The diabetes risk for female weight cyclers in the urban region was non-significantly lower than or similar to the risk for those maintaining a stable weight. The data for Japanese men and women residing in the rural region also suggest no risk and a non-significantly lower risk, respectively, of diabetes with weight cycling compared with maintaining a stable weight (table 3). These results are reinforced by a sensitivity analysis with a restriction on middle-aged individuals, yielding almost same ORs without statistical significance (table 4) as those of the entire population.

These observations are not consistent with those from previous studies of Western populations, which showed that weight cycling significantly or non-significantly increased the risk of diabetes for point estimates. In the Framingham Heart Study, approximately 1 kg/m^2 of weight cycling in middle-aged Americans carried a hazard ratio of 1.1 (95%CI: 0.8–1.5) for the risk of diabetes after adjusting for sex and BMI at 25 years of age.[4] In the American middle-aged women of the National Health and Nutrition Examination Survey, weight cycling of 4.5–9.1 kg and 9.1–22.2 kg with an intentional weight loss three or more times in four years carried ORs for the risk of diabetes of 1.11 (95%CI: 0.89–1.37) and 1.39 (95%CI: 0.90–2.13), respectively.[5] A study from a cohort of medical students at the Johns Hopkins University School of Medicine reported that the highest quartile of BMI variability for ages between 25 and 45 years had an OR of 2.1 (95%CI: 1.0–4.6) for the risk of diabetes after 50 years, compared with the other three lower quartiles.[21] In a large German cohort, weight

For peer review only - http://bmjopen.bmj.com/site/about/guidelines.xhtml

cycling of ≥1.5 kg/year was significantly associated with an adjusted hazard ratio of 1.34.[6]

The disparity between the results of this study and the Western studies may be due to ethnic differences in diet,[22] the capacity to gain weight[23] and self-consciousness about body weight.[24] Further research is required to explore why the relationship between weight cycling and risk of diabetes is inversed between Western and East Asian populations. The reason may be attributable to different motivations to lose weight in the context of different diet cultures and body self-consciousness.[25] The East Asians who try to lose weight may be particularly those who are relatively concerned about the poor health outcomes of being overweight. Westerners described in the study cohorts who tried to lose weight may, in an extreme expression, have been those who lost and regained a great deal of weight and potentially ran the risk of poor health outcomes.

From the data from the urban region, a weight increase of more than +5 kg above the participant's weight at the age of 20 years increased the risk of developing diabetes with a dose–response relationship in both men and women (table 2). Furthermore, in both sexes, an increase in weight of more than +10 kg above that when aged 20 years more than doubles the risk of diabetes, with statistical significance, compared to the risk for those who maintained their weight within ±5 kg of their weight when aged 20 years. These results agree with the study involving a US cohort, which reported a relative risk of 3.2 (95%CI: 1.4–7.4) for the highest quartile of an increase in BMI from 25 to 45 years of age in comparison with the other three lower quartiles.[21] A dose–response relationship of weight change from that aged 20 years to the risk of diabetes occurred in the Japanese women residing in the urban region, with ORs of 0.48 (95%CI: 0.18–1.25)

For peer review only - http://bmjopen.bmj.com/site/about/guidelines.xhtml

for a change less than 5 kg, 0.96 (95%CI: 0.55–1.65) for an increase of between 5 and 10 kg and 2.10 (95%CI: 1.20–3.67) for an increase greater than 10 kg (table 2). In contrast, the Japanese men who had lost 5 kg or more of body weight between the age of 20 years and early middle age had an OR of 1.68 (95%CI: 0.75–3.80). This paradoxically increased OR was most likely due to the small number of participants (seven) who developed diabetes among a weight loss group of 1438 people.

This study had several limitations. The first of these was the threshold of ±4% weight change in 10 years. This threshold referred to a study from United Kingdom on the association between weight change and the risk of diabetes.[26] The threshold of a ±4% weight change was calculated as approximately 1 BMI unit in Japanese people with a mean BMI of 23 kg/m^2. However, the threshold for categorisation of weight cycling should vary according to mean BMIs in different ethnicities, in which people have different insulin sensitivities.[13 27] Second, we did not evaluate insulin sensitivity. Measuring fasting plasma glucose and insulin concentration to calculate HOMA-IR,[28] an index of insulin resistance, would have allowed us to assess the association between weight cycling and the physiological hazard of diabetes. Third, the weight changes recorded in the rural region may be misclassified due to missing data for the years when participants did not undergo the health examination. However, such misclassification would bias the ORs to the null hypothesis, and we believe that such a bias would not change the conclusions for the rural region. Fourth, we could not examine whether the weight cycling was intentional; however, we consider that a subset of unintentional weight loss could be attributed to metabolic diseases, and patients with such diseases could not usually regain the weight within a short duration. Fifth, the follow-up duration

For peer review only

For peer review only - http://bmjopen.bmj.com/site/about/guidelines.xhtml

and the numbers of weight measurements vary among the groups of weight change patterns. However, because perfect categorisation of changing weight over time would be impossible, we think that this study design could answer the study question. Sixth, the urban data for the weight at 20 years of age were derived from the participants' memory. Thus, recall bias could have existed in the results. Seventh, a subset of the diagnoses in this study were not made by physicians but via an epidemiological criteria.[17] However, most observational studies by nature rely on epidemiological criteria for detecting diabetes, and the use of a consistent diagnostic criterion can allow researchers to compare the risk of diabetes onset between the reference group and that of interest. Last, this study lacks the information pertaining to lifestyle, including diet, marriage status, job type and owning a car, that may have partly explain the association between weight cycling and incident diabetes.

The present study also has several strengths. First, to the best of our knowledge, this is the first study to explore the relationship between weight cycling and the risk of diabetes in Asians. Since the relationship in this study was almost opposite to that of Americans, further research in East Asians is necessary to confirm this relationship. Next, this study was conducted in two differently characterised populations (urban and rural residents). The ORs of the risk of diabetes were 1.05 (95%CI: 0.57–1.95) in the weight gain-loss pattern of the urban resident women (table 2) and 1.58 (95%CI: 0.78–3.17) in weight loss-gain pattern of the rural resident men (table 3). However, all other weight cycling patterns for both sexes in the urban and rural regions were negatively correlated with the risk of diabetes with and without statistical significance (tables 2 and 3). Third, the number of participants in both sexes were approximately 10,000 in the

For peer review only - http://bmjopen.bmj.com/site/about/guidelines.xhtml

urban region and approximately 5,000 in the rural region. Since the present study included a large number of participants from both urban and rural Japan, Japanese and East Asian weight cyclers could refer to its results.

This study could contribute to aiding public health practitioners and on-site clinical professionals prevent diabetes in the general population. A sustained weight gain greater than 4% over ten years in middle age (table 4) and more than 5 kg of additional weight gain since the age of 20 years (table 2) both may carry an increased risk of diabetes for both sexes. Since middle-aged people can easily undergo such a small weight gain over the short or long term, non-diabetic people within the normal BMI range should be cautious about the risks resulting from even such a slight weight gain through their lifetime. An interventional study indicated that weight loss (−1.8 kg/m^2 of BMI in a diet intervention group and −3.3 kg/m^2 of BMI in a diet-and-exercise intervention group) improved insulin sensitivity in Japanese patients with obesity and type 2 diabetes.[29] Improved insulin sensitivity was also observed in Americans who maintained their weight with treadmill-based exercise and no alteration in their diet.[30] In addition, studies have demonstrated that building muscle through exercising without changing weight improves insulin sensitivity.[31]

While not statistically significant, two profiles of weight cycling pattern in the present study resulted in an increased risk of diabetes whereas the risk decreased for the other six profiles (tables 2 and 3). Since better insulin sensitivity directly leads to a decreased risk of diabetes, the results may be due to the differences in how the participants lost or gained weight (i.e. whether they lost or gained fat or muscle mass). Recent studies have indicated that sarcopenia, the loss of skeletal muscle alone or with

For peer review only - http://bmjopen.bmj.com/site/about/guidelines.xhtml

increased fat mass in ageing, is a leading cause of death in old age.[32] The results of the present study, in the context of previous studies, suggest that over both the short- and long-term, people might reduce their risk of diabetes by losing fat and maintaining muscle mass.

CONCLUSIONS

In men in an urban region of Japan, weight cycling was associated with a significant reduction in the risk of diabetes; however, a clear association was not observed in either women of the urban region or in men and women of a rural region. The results were different than those recorded in Western countries and may be attributed to differences in diet, endocrinological capacity to gain weight and weight-consciousness. In addition, the risk of diabetes increases linearly with weight gain from the age of 20 years in Japanese urban men and women. A study that includes the measurement of insulin sensitivity is necessary to confirm the present results and to improve the understanding of the risks for East Asian weight cyclers.

(3,508 words; limit of 4,000 words)

For peer review only

For peer review only - http://bmjopen.bmj.com/site/about/guidelines.xhtml

Acknowledgments We thank all participants in this study and staff at the St. Luke's International Hospital and the Yamanashi Koseiren Health Care Center for their support, which made this study possible.

Competing interests None declared.

Funding This work was supported by the Ministry of Education, Culture, Sports, Science and Technology of Japan (MEXT) (KAKENHI grant number: JP15K08730 and JP15K15221). The funder had no role in study design, analysis, decision to publish or preparation of the manuscript.

Contributors ZY, YY, MT and OT: setting up the study and data collection. HY, ZY, MM and AT: designing the study. HY and AT: data analysis. HY: writing and revising the draft. ZY, SO, MM, YA and HY: development of the discussion section. All authors read and approved the final manuscript.

Ethics approval The ethics committee of the School of Medicine, University of Yamanashi (approval number: H27-1417).

Data sharing statement No additional data are available.

For peer review only

REFERENCES

1. Nanri A, Mizoue T, Takahashi Y, et al. Association of weight change in different periods of adulthood with risk of type 2 diabetes in Japanese men and women: the Japan Public Health Center-Based Prospective Study. *J Epidemiol Commun H* 2011;**65**:1104–10.
2. Wannamethee SG, Shaper AG. Weight change and duration of overweight and obesity in the incidence of type 2 diabetes. *Diabetes Care* 1999;**22**:1266–72.
3. Ford ES, Williamson DF, Liu SM. Weight change and diabetes incidence: findings from a national cohort of US adults. *Am J Epidemiol* 1997;**146**:214–22.
4. Waring ME, Eaton CB, Lasater TM, et al. Incident diabetes in relation to weight patterns during middle age. *Am J Epidemiol* 2010;**171**:550–6.
5. Field AE, Manson JE, Laird N, et al. Weight cycling and the risk of developing type 2 diabetes among adult women in the United States. *Obes Res* 2004;**12**:267–74.
6. Neamat-Allah J, Barrdahl M, Hüsing A, et al. Weight cycling and the risk of type 2 diabetes in the EPIC-Germany cohort. *Diabetologia* 2015;**58**:2718–25.
7. French S, Folsom A, Jeffery R, Zheng W, Mink P, Baxter J. Weight variability and incident disease in older women: the Iowa Women's Health Study. *Int J Obes Relat Metab Disord* 1997;**21**:217–23.
8. Morris RD, Rimm AA. Long-term weight fluctuation and non-insulin-dependent diabetes mellitus in white women. *Ann Epidemiol* 1992;**2**:657–64.
9. Mukai T, Crago M, Shisslak CM. Eating attitudes and weight preoccupation among female high school students in Japan. *J Child Psychol Psychiatry* 1994;**35**:677–88.

For peer review only

For peer review only - http://bmjopen.bmj.com/site/about/guidelines.xhtml

10. Swami V, Frederick DA, Aavik T, et al. The attractive female body weight and female body dissatisfaction in 26 countries across 10 world regions: results of the International Body Project I. *Personal Soc Psychol Bull* 2010;**36**:309–25.

11. Chisuwa N, O'Dea JA. Body image and eating disorders amongst Japanese adolescents. a review of the literature. *Appetite* 2010;**54**:5–15.

12. Xu X, Mellor D, Kiehne M, et al. Body dissatisfaction, engagement in body change behaviours and sociocultural influences on body image among Chinese adolescents. *Body Image* 2010;**7**:156–64.

13. Kodama K, Tojjar D, Yamada S, et al. Ethnic differences in the relationship between insulin sensitivity and insulin response: a systematic review and meta-analysis. *Diabetes Care* 2013;**36**:1789–96.

14. Matsushita Y, Yoshiike N, Kaneda F, Yoshita K, Takimoto H. Trends in childhood obesity in Japan over the last 25 years from the national nutrition survey. *Obes Res* 2004;**12**:205–14.

15. Yoshiike N, Seino F, Tajima S, et al. Twenty□year changes in the prevalence of overweight in Japanese adults: The National Nutrition Survey 1976–95. *Obes Rev* 2002;**3**:183–90.

16. Ministry of Health, Labour and Welfare of Japan. Industrial Safety and Health Act (English version). 1972. http://www.japaneselawtranslation.go.jp/law/detail/?printID=&id=1926&re=&vm=02 (accessed 24 February 2017).

17. American Diabetes Association. Diagnosis and classification of diabetes mellitus. *Diabetes Care* 2010;**33**:S62–S69.

For peer review only

For peer review only - http://bmjopen.bmj.com/site/about/guidelines.xhtml

18. The International Expert Committee. International Expert Committee report on the role of the A1C assay in the diagnosis of diabetes. *Diabetes Care* 2009;**32**:1327–34.

19. Kaufman J, Poole C. Looking back on "causal thinking in the health sciences". *Annu Rev Publ Health* 2000;**21**:101-19.

20. NCD Risk Factor Collaboration. Worldwide trends in diabetes since 1980: a pooled analysis of 751 population-based studies with 4· 4 million participants. *Lancet* 2016;**387**:1513–30.

21. Brancati FL, Wang N, Mead LA, et al. Body weight patterns from 20 to 49 years of age and subsequent risk for diabetes mellitus: the Johns Hopkins Precursors Study. *Arch Int Med* 1999;**159**:957–63.

22. Slocum R. Race in the study of food. *Prog Hum Geogr* 2011;**35**:303–27.

23. The DECODE–DECODA Study Group. Age, body mass index and type 2 diabetes—associations modified by ethnicity. *Diabetologia* 2003;**46**:1063–70.

24. Mintz LB, Kashubeck S. Body image and disordered eating among Asian American and Caucasian college students an examination of race and gender differences. *Psychol Women Q* 1999;**23**:781–96.

25. Frederick DA, Forbes GB, Grigorian KE, et al. The UCLA Body Project I: gender and ethnic differences in self-objectification and body satisfaction among 2,206 undergraduates. *Sex Roles* 2007;**57**:317–27.

26. Wannamethee SG, Shaper AG, Walker M. Overweight and obesity and weight change in middle aged men: impact on cardiovascular disease and diabetes. *J Epidemiol Commun H* 2005;**59**:134–9.

For peer review only

For peer review only - http://bmjopen.bmj.com/site/about/guidelines.xhtml

27. Ntuk UE, Gill JMR, Mackay DF, et al. Ethnic-specific obesity cutoffs for diabetes risk: cross-sectional study of 490,288 UK Biobank participants. *Diabetes Care* 2014;**37**:2500–7.

28. Matthews D, Hosker J, Rudenski A, et al. Homeostasis model assessment: insulin resistance and β-cell function from fasting plasma glucose and insulin concentrations in man. *Diabetologia* 1985;**28**:412–9.

29. Yamanouchi K, Shinozaki T, Chikada K, et al. Daily walking combined with diet therapy is a useful means for obese NIDDM patients not only to reduce body weight but also to improve insulin sensitivity. *Diabetes Care* 1995;**18**:775–8.

30. Duncan GE, Perri MG, Theriaque DW, et al. Exercise training, without weight loss, increases insulin sensitivity and postheparin plasma lipase activity in previously sedentary adults. *Diabetes Care* 2003;**26**:557–62.

31. Nassis GP, Papantakou K, Skenderi K, et al. Aerobic exercise training improves insulin sensitivity without changes in body weight, body fat, adiponectin, and inflammatory markers in overweight and obese girls. *Metabolism* 2005;**54**:1472–9.

32. Fielding RA, Vellas B, Evans WJ, et al. Sarcopenia: an undiagnosed condition in older adults. Current consensus definition: prevalence, etiology, and consequences. International Working Group on Sarcopenia. *J Am Med Dir Assoc* 2011;**12**:249–56.

For peer review only - http://bmjopen.bmj.com/site/about/guidelines.xhtml

Figure 1. Scheme of how participants were categorised into the five weight change patterns

For peer review only

For peer review only - http://bmjopen.bmj.com/site/about/guidelines.xhtml

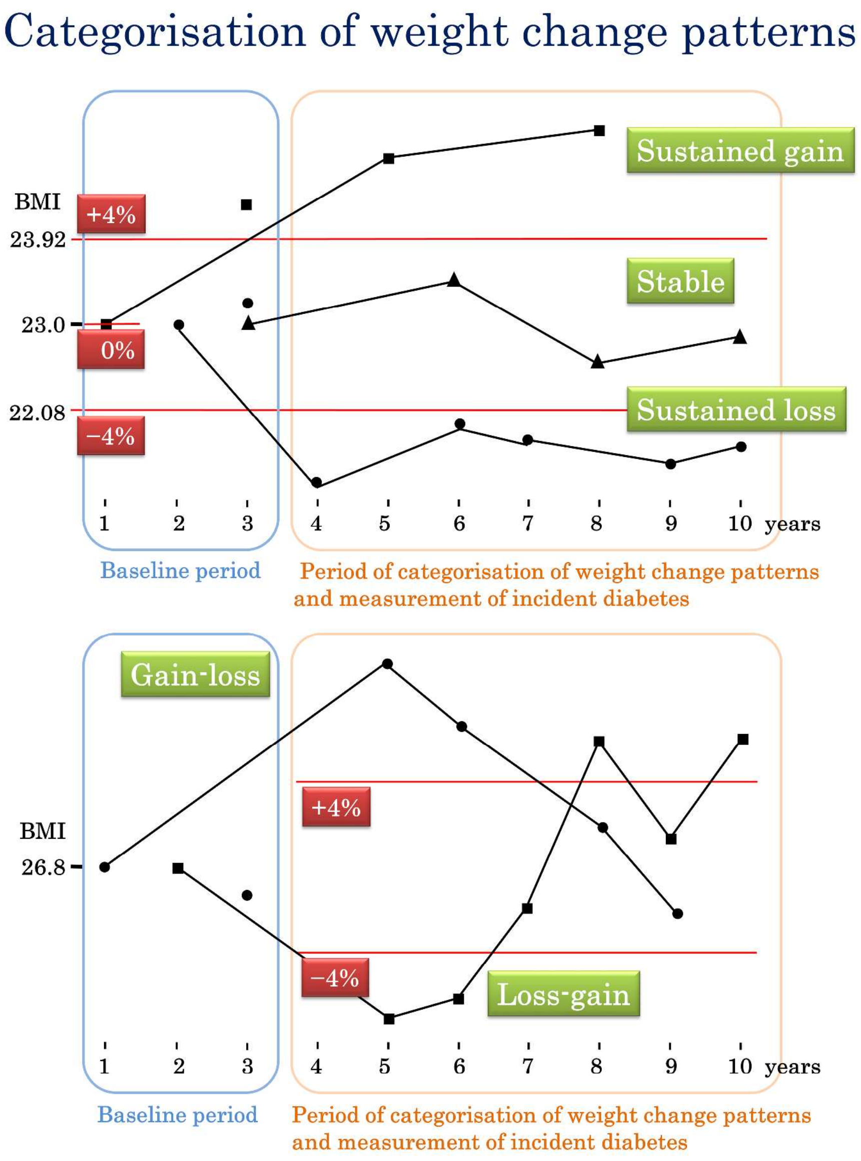

Scheme of how participants were categorised into the five weight change patterns
figure 1
339x454mm (300 x 300 DPI)

Supplementary. Baseline characteristics among groups of weight change patterns over 10 years

**Men in urban Japan (n = 10,094)**	Sustained loss	Loss-gain	Stable	Gain-loss	Sustained gain
Age, years	52.3 (12.2)	51.2 (12.0)	50.3 (11.3)	46.0 (11.4)	44.9 (11.3)
Weight, kg	70.8 (10.4)	69.8 (9.5)	68.3 (9.0)	67.3 (9.5)	67.8 (10.8)
Height, cm	170.1 (6.2)	170.3 (6.1)	170.2 (6.0)	170.7 (6.1)	171.2 (6.1)
Body mass index, kg/m2	24.4 (3.0)	24.0 (2.7)	23.5 (2.6)	23.1 (2.8)	23.1 (3.2)
HbA1c, %	5.4 (0.4)	5.4 (0.3)	5.4 (0.3)	5.3 (0.3)	5.3 (0.3)
HbA1c, mmol/mol	35.9 (3.9)	35.7 (3.7)	35.3 (3.7)	34.6 (3.7)	34.6 (3.4)
Fasting plasma glucose, mg/dL	102.3 (9.2)	101.5 (8.8)	101.0 (8.5)	99.0 (8.0)	99.8 (8.4)
Fasting plasma glucose, mmol/L	5.7 (0.5)	5.6 (0.5)	5.6 (0.5)	5.5 (0.4)	5.5 (0.5)
Weight change from 20 years of age, kg	+9.7 (9.1)	+9.0 (8.1)	+8.2 (7.9)	+7.0 (8.4)	+7.0 (20.5)
Current smoking, %	20.0	22.8	23.6	31.3	33.7
Usually drinking alcohol, %	61.7	62.3	63.8	57.3	55.2
Duration of walking per day, min	41.0 (38.3)	41.3 (41.7)	43.3 (41.5)	40.4 (35.2)	38.6 (36.0)
Physical activity of 6–7/week, %	11.2	12.0	11.9	10.4	11.6

**Women in urban Japan (n = 10,614)**	Sustained loss	Loss-gain	Stable	Gain-loss	Sustained gain
Age, years	52.4 (10.7)	51.1 (11.5)	48.9 (11.3)	45.7 (10.6)	43.5 (9.8)
Weight, kg	53.8 (8.0)	53.4 (7.8)	51.7 (7.0)	51.8 (7.1)	51.6 (7.4)
Height, cm	156.9 (5.7)	157.2 (5.9)	157.8 (5.6)	158.1 (5.5)	158.4 (5.3)
Body mass index, kg/m2	21.8 (3.0)	21.6 (3.0)	20.8 (2.7)	20.7 (2.7)	20.6 (2.8)
HbA1c, %	5.5 (0.4)	5.4 (0.4)	5.4 (0.3)	5.3 (0.3)	5.3 (0.3)
HbA1c, mmol/mol	36.1 (4.1)	35.4 (3.8)	35.0 (3.8)	34.4 (3.6)	34.0 (3.7)
Fasting plasma glucose, mg/dL	96.5 (8.9)	95.7 (8.1)	94.6 (8.0)	93.2 (7.3)	92.8 (7.3)

For peer review only

Yokomichi H et al.

Fasting plasma glucose, mmol/L	5.4 (0.5)	5.3 (0.4)	5.3 (0.4)	5.2 (0.4)	5.2 (1.4)
Weight change from 20 years of age, kg	+4.1 (7.4)	+3.8 (7.3)	+2.4 (7.1)	+2.6 (7.1)	+2.2 (7.5)
Current smoking, %	6.6	6.7	5.7	9.9	11.8
Usually drinking alcohol, %	23.7	26.7	27.3	29.4	28.3
Duration of walking per day, min	42.9 (49.4)	41.2 (49.8)	42.3 (40.9)	41.5 (40.5)	43.8 (51.9)
Physical activity of 6–7/week, %	12.1	10.6	11.3	8.6	8.8
**Men in rural Japan (n = 4,818)**	Sustained loss	Loss-gain	Stable	Gain-loss	Sustained gain
Age, years	53.5 (10.3)	51.7 (9.8)	52.3 (10.1)	49.2 (10.1)	48.3 (10.5)
Weight, kg	67.2 (9.0)	66.2 (8.7)	66.0 (9.1)	64.7 (8.6)	64.3 (9.9)
Height, cm	167.5 (6.2)	168.0 (6.0)	167.8 (6.2)	168.5 (6.2)	168.8 (6.5)
Body mass index, kg/m2	23.9 (2.6)	23.4 (2.6)	23.4 (2.7)	22.8 (2.5)	22.5 (2.9)
HbA1c, %	5.3 (0.4)	5.3 (0.4)	5.3 (0.3)	5.2 (0.3)	5.2 (0.3)
HbA1c, mmol/mol	34.7 (3.9)	34.6 (3.9)	34.3 (3.7)	33.9 (3.8)	33.8 (3.5)
Fasting plasma glucose, mg/dL	97.5 (9.2)	96.4 (9.0)	96.9 (9.1)	95.1 (8.9)	95.1 (8.5)
Fasting plasma glucose, mmol/L	5.4 (0.5)	5.4 (0.5)	5.4 (0.5)	5.3 (0.5)	5.3 (0.5)
Current smoker, %	38.5	42.7	37.7	49.3	58.5
Current drinker, %	79.5	77.5	78.4	75.7	77.5
**Women in rural Japan (n = 4,852)**	Sustained loss	Loss-gain	Stable	Gain-loss	Sustained gain
Age, years	54.9 (9.0)	52.8 (8.6)	53.1 (9.2)	50.7 (9.2)	48.2 (9.9)
Weight, kg	54.0 (8.1)	53.6 (7.5)	52.9 (6.9)	52.6 (7.6)	52.6 (7.8)
Height, cm	154.1 (5.6)	154.8 (5.8)	154.6 (5.4)	155.3 (5.5)	155.7 (5.6)
Body mass index, kg/m2	22.7 (3.1)	22.4 (2.8)	22.1 (2.8)	21.8 (2.9)	21.7 (3.0)
HbA1c, %	5.3 (0.3)	5.3 (0.3)	5.3 (0.3)	5.3 (0.3)	5.2 (0.3)
HbA1c, mmol/mol	34.9 (3.7)	34.6 (3.4)	34.6 (3.6)	34.0 (3.4)	33.6 (3.3)

For peer review only

Fasting plasma glucose, mg/dL	94.1 (9.1)	93.0 (8.3)	93.7 (8.8)	93.0 (8.0)	91.9 (7.9)
Fasting plasma glucose, mmol/L	5.2 (0.5)	5.2 (0.5)	5.2 (0.5)	5.2 (0.4)	5.1 (0.4)
Current smoker, %	5.5	8.1	6.6	8.5	11.5
Current drinker, %	24.2	27.2	26.5	27.1	30.9

The data are presented as the mean (SD) or %.

For peer review only

STROBE Statement—checklist of items that should be included in reports of observational studies

	Item No.	Recommendation	Page No.	Relevant text from manuscript
**Title and abstract**	1	(*a*) Indicate the study's design with a commonly used term in the title or the abstract	1	Line 1–2
		(*b*) Provide in the abstract an informative and balanced summary of what was done and what was found	2	Line 23–41
**Introduction**				
Background/rationale	2	Explain the scientific background and rationale for the investigation being reported	5	Line 54–70
Objectives	3	State specific objectives, including any prespecified hypotheses	5	Line 70–71
**Methods**				
Study design	4	Present key elements of study design early in the paper	6	Line 76–77
Setting	5	Describe the setting, locations, and relevant dates, including periods of recruitment, exposure, follow-up, and data collection	6	Line 77–92
Participants	6	(*a*) *Cohort study*—Give the eligibility criteria, and the sources and methods of selection of participants. Describe methods of follow-up<br>*Case-control study*—Give the eligibility criteria, and the sources and methods of case ascertainment and control selection. Give the rationale for the choice of cases and controls<br>*Cross-sectional study*—Give the eligibility criteria, and the sources and methods of selection of participants	6	Line 86–91
		(*b*) *Cohort study*—For matched studies, give matching criteria and number of exposed and unexposed<br>*Case-control study*—For matched studies, give matching criteria and the number of controls per case		NA
Variables	7	Clearly define all outcomes, exposures, predictors, potential confounders, and effect modifiers. Give diagnostic criteria, if applicable	6	Line 92–94
Data sources/ measurement	8*	For each variable of interest, give sources of data and details of methods of assessment (measurement). Describe comparability of assessment methods if there is more than one group	6–7	Line 99–119
Bias	9	Describe any efforts to address potential sources of bias	5	Line 79–86
Study size	10	Explain how the study size was arrived at	8	Line 138–139, 144–145

Continued on next page

For peer review only - http://bmjopen.bmj.com/site/about/guidelines.xhtml

Quantitative variables	11	Explain how quantitative variables were handled in the analyses. If applicable, describe which groupings were chosen and why	7	Line 115–119
Statistical methods	12	(*a*) Describe all statistical methods, including those used to control for confounding	7–8	Line 122–131
		(*b*) Describe any methods used to examine subgroups and interactions	8	Line 128–131
		(*c*) Explain how missing data were addressed	6	Line 88–89
		(*d*) *Cohort study*—If applicable, explain how loss to follow-up was addressed<br>*Case-control study*—If applicable, explain how matching of cases and controls was addressed<br>*Cross-sectional study*—If applicable, describe analytical methods taking account of sampling strategy		NA
		(*e*) Describe any sensitivity analyses	8	Line 128–131
**Results**				
Participants	13*	(a) Report numbers of individuals at each stage of study—eg numbers potentially eligible, examined for eligibility, confirmed eligible, included in the study, completing follow-up, and analysed	8	Line 138–139, 144–145
		(b) Give reasons for non-participation at each stage	6	Line 90–91
		(c) Consider use of a flow diagram		NA
Descriptive data	14*	(a) Give characteristics of study participants (eg demographic, clinical, social) and information on exposures and potential confounders	9	Table 1
		(b) Indicate number of participants with missing data for each variable of interest		NA
		(c) *Cohort study*—Summarise follow-up time (eg, average and total amount)	10	Line 155–171
Outcome data	15*	*Cohort study*—Report numbers of outcome events or summary measures over time	11–14	Tables 2 and 3
		Case-control study—Report numbers in each exposure category, or summary measures of exposure		NA
		Cross-sectional study—Report numbers of outcome events or summary measures		NA
Main results	16	(*a*) Give unadjusted estimates and, if applicable, confounder-adjusted estimates and their precision (eg, 95% confidence interval). Make clear which confounders were adjusted for and why they were included	11–14	Tables 2 and 3
		(*b*) Report category boundaries when continuous variables were categorized	11–15 (BMI)	Tables 2–4
		(*c*) If relevant, consider translating estimates of relative risk into absolute risk for a meaningful time period		NA

For peer review only - http://bmjopen.bmj.com/site/about/guidelines.xhtml

Continued on next page Other analyses	17	Report other analyses done—eg analyses of subgroups and interactions, and sensitivity analyses	14–15	Table 4
**Discussion**				
Key results	18	Summarise key results with reference to study objectives	16	Line 205–213
Limitations	19	Discuss limitations of the study, taking into account sources of potential bias or imprecision. Discuss both direction and magnitude of any potential bias	18–19	Line 256–284
Interpretation	20	Give a cautious overall interpretation of results considering objectives, limitations, multiplicity of analyses, results from similar studies, and other relevant evidence	20–21	Line 313–322
Generalisability	21	Discuss the generalisability (external validity) of the study results	20	Line 299–312
**Other information**				
Funding	22	Give the source of funding and the role of the funders for the present study and, if applicable, for the original study on which the present article is based	22	Line 339–342

*Give information separately for cases and controls in case-control studies and, if applicable, for exposed and unexposed groups in cohort and cross-sectional studies.

Note: An Explanation and Elaboration article discusses each checklist item and gives methodological background and published examples of transparent reporting. The STROBE checklist is best used in conjunction with this article (freely available on the Web sites of PLoS Medicine at http://www.plosmedicine.org/, Annals of Internal Medicine at http://www.annals.org/, and Epidemiology at http://www.epidem.com/). Information on the STROBE Initiative is available at www.strobe-statement.org.

For peer review only - http://bmjopen.bmj.com/site/about/guidelines.xhtml

Weight cycling and the subsequent onset of type 2 diabetes mellitus: 10-year cohort studies in urban and rural Japan

Journal:	*BMJ Open*
Manuscript ID	bmjopen-2016-014684.R3
Article Type:	Research
Date Submitted by the Author:	15-Mar-2017

Complete List of Authors:	Yokomichi, Hiroshi; University of Yamanashi, Department of Health Sciences<br>Ohde, Sachiko; St. Luke's International University, Center for Clinical Epidemiology<br>Takahashi, Osamu; St. Luke's International University, Center for Clinical Epidemiology<br>Mochizuki, Mie; University of Yamanashi, Department of Pediatrics<br>Takahashi, Atsunori; University of Yamanashi, Department of Health Sciences<br>Yoda, Yoshioki; Yamanashi Koseiren Health Care Center<br>Tsuji, Masahiro; Yamanashi Koseiren Health Care Center<br>Akiyama, Yuka; University of Yamanashi, Department of Health Sciences<br>Yamagata, Zentaro; The University of Yamanashi, Interdisciplinary Graduate School of Medicine and Engineering

**Primary Subject Heading**:	Epidemiology
Secondary Subject Heading:	Diabetes and endocrinology, Health services research, Nutrition and metabolism, Sports and exercise medicine
Keywords:	body weight changes, type 2 diabetes, body mass index, Asian, sarcopenia

SCHOLARONE™ Manuscripts

Weight cycling and the subsequent onset of type 2 diabetes mellitus: 10-year cohort studies in urban and rural Japan

Hiroshi Yokomichi[1*], Sachiko Ohde[2], Osamu Takahashi[2], Mie Mochizuki[3], Atsunori Takahashi[1], Yoshioki Yoda[4], Masahiro Tsuji[4], Yuka Akiyama[1] and Zentaro Yamagata[1]

[1]Department of Health Sciences, University of Yamanashi, Yamanashi, Japan

[2]Center for Clinical Epidemiology, St. Luke's International University, Tokyo, Japan

[3]Department of Pediatrics, University of Yamanashi, Yamanashi, Japan

[4]Yamanashi Koseiren Health Care Center, Yamanashi, Japan

[*]Corresponding author:

Hiroshi Yokomichi

1110 Shimokato, Chuo City, Yamanashi 4093898, Japan

E-mail: hyokomichi@yamanashi.ac.jp

Tel: +81-(0)80-5524-7303

Keywords: body weight changes; type 2 diabetes; body mass index; Asian; sarcopenia

Word count: 2,718

For peer review only - http://bmjopen.bmj.com/site/about/guidelines.xhtml

ABSTRACT

Objective: To investigate how weight cycling (gaining and losing weight) affects the risk of diabetes.

Design: Cohort studies.

Setting: Primary healthcare in urban and rural Japan.

Participants: 20,708 urban and 9,670 rural residents.

Primary outcome measures: Odds ratios (ORs) for diabetes in those with weight loss, weight loss-gain, stable weight, weight gain-loss and weight gain over 10 years. Weight gain and loss were defined as a change of more than ±4% from baseline weight.

Results: In the urban region, the ORs relative to the stable group for the loss-gain and gain-loss groups were 0.63 (95% CI: 0.45–0.89) and 0.51 (95% CI: 0.32–0.82) for men and 0.72 (95% CI: 0.39–1.34) and 1.05 (95% CI: 0.57–1.95) for women. In the rural region, they were 1.58 (95% CI: 0.78–3.17) and 0.44 (95% CI: 0.15–1.29) in men and 0.41 (95% CI: 0.12–1.44) and 0.77 (95% CI: 0.28–2.14) in women. The ORs for an increase in weight between 5 and 10 kg from the age of 20 years were 1.54 (95% CI: 1.03–2.30) in men and 0.96 (95% CI: 0.55–1.65) in women.

Conclusions: In Japan, weight cycling was associated with a significant reduction in the risk of diabetes for men from urban regions. The associations were unclear for women from urban regions and both men and women from rural regions. These results differ from those in Western studies, probably because of differences in diet, insulin secretion and sensitivity and weight-consciousness.

For peer review only

For peer review only - http://bmjopen.bmj.com/site/about/guidelines.xhtml

43

44 **Word count: 245 words (limit: 300 words)**

For peer review only

For peer review only - http://bmjopen.bmj.com/site/about/guidelines.xhtml

Strengths and limitations of this study

- Participants were invited from both urban and rural Japan.
- Several weight change patterns, including weight gain after loss and loss after gain, were measured.
- Odds ratios may change with weight changes of more than ±4% in 10 years.
- The levels of insulin secretion and sensitivity were not measured.
- Whether participants' weight loss was intentional was undetermined.

For peer review only - http://bmjopen.bmj.com/site/about/guidelines.xhtml

INTRODUCTION

Weight gain is a well-known risk factor for incidental type 2 diabetes. Research involving people of Western, Oriental and African descent has quantitatively established the risks of developing diabetes linked to weight gain.[1-3] Researchers have also raised the question of whether repeatedly gaining and losing weight (weight cycling) is an independent risk factor for developing diabetes. Studies on this topic have reported inconsistent results in Westerners in Europe and North America. Several prospective studies suggest that weight cycling is a risk factor for type 2 diabetes, but others do not.[4-8] To our knowledge, the risk of diabetes in Asian weight cyclers has not been researched.

There is a preconception linking being slim to an aesthetic standard,[9 10] and many Asian women therefore try to lose weight.[11 12] Another group likely to try to reduce their weight is middle-aged Asian businesspeople with few opportunities for physical activity. Recent studies have demonstrated that East Asians are much more likely than Westerners to develop type 2 diabetes at a lower body mass index (BMI).[13] It may therefore take only a small change in weight to alter the risk of diabetes for this group. The literature indicates that in Japan, diet, physical activity, prevalence of overweight individuals and aesthetic consciousness are different among urban and rural residents.[14 15] The aim of this study was to establish whether weight cyclers in Japan were at an increased risk of diabetes. We used Japanese urban and rural data to examine this question in populations with varying lifestyles.

For peer review only - http://bmjopen.bmj.com/site/about/guidelines.xhtml

METHODS

Study participants and measurements

These cohort studies involved participants from both an urban area, Tokyo and a rural area, Yamanashi Prefecture. In Tokyo, participants were employees of private companies who underwent medical check-ups between January 2005 and December 2014 at St. Luke's International Hospital. These annual check-ups were based on a legal obligation imposed by the Industrial Safety and Health Act in Japan.[16] In Yamanashi Prefecture, participants were employees and also residents who paid for a private comprehensive medical check-up between April 1999 and March 2009 at the Yamanashi Koseiren Health Care Center. A subset of participants in Yamanashi used a subsidy from their employers or administrative agencies for this check-up. Those from the urban area therefore received approximately annual medical check-ups over a 10-year period, and those in the rural area received occasional voluntary check-ups. Participants were included in the analysis if they had no diagnosis of diabetes and an HbA1c less than 6.5% (48 mmol/mol) during a baseline period of the first three years of the 10-year period. If they attended two or three medical check-ups in the first three years, the data from the first visit were used as the baseline. Participants were also required to attend at least two medical check-ups during the last 7 years of the study. They therefore received three to eight medical check-ups over the period, to enable us to categorise weight change patterns (exposure). The onset of diabetes was indicated by the results of questions about the diagnosis of diabetes, the commencement of diabetic therapies or glycated haemoglobin (HbA1c) ≥ 6.5% (48 mmol/mol).[17 18] In Tokyo, trained nurses interviewed participants over 20 years old to establish changes in weight. BMI was calculated as the participant's weight in kilograms divided by the square of their height in metres.

For peer review only - http://bmjopen.bmj.com/site/about/guidelines.xhtml

101 **Weight change categories**

102 The participants were categorised into five groups by their pattern of weight change during the 3–10 years after the baseline (Figure 1). The stable group included all participants whose weight did not change by more than ±4% from the baseline. The sustained gain group consisted of those who gained more than 4% of their baseline weight and did not subsequently lose it again. The sustained loss group included those who lost more than 4% of their baseline weight and did not subsequently regain it. The gain-loss group included all participants who gained more than 4% of their baseline weight, but later brought their weight back below +4%. The loss-gain group included participants who lost more than 4% of their baseline weight but brought their weight back above −4%. When the participants had been categorised, we measured whether they developed diabetes. The length of time over which the participants were observed to see if they developed diabetes was between 1 and 6 years. Any data measured after a diagnosis of diabetes were ignored to conserve the temporality of exposure to outcome for epidemiological causation.[19]

115 The ±4% change in weight used for this categorisation was considered to be approximately a one-unit change for a person with a BMI of 22 kg/m^2. This was based on the 2014 reference mean BMIs for Japanese men and women of 23.6 kg/m^2 and 21.7 kg/m^2.[20] The gain-loss and loss-gain groups were the weight cyclers of interest in this study.

120 **Statistical analysis**

121 The baseline characteristics recorded for the participants included age, weight, height, BMI, HbA1c and fasting plasma glucose. We used univariate and multivariable logistic regressions to compare the risk of diabetes between the groups. In the data from the urban group, the

For peer review only - http://bmjopen.bmj.com/site/about/guidelines.xhtml

covariates used for the adjustment at baseline were age, weight change from the age of 20, BMI, smoking habits, alcohol consumption and physical activity. In the data from the rural group, the available covariates for the adjustment at baseline were age, BMI, smoking habits and alcohol consumption. Means of follow-up duration and numbers of measurements for each weight change pattern group were calculated. The analyses were stratified by sex. Another focus of this study was the impact of weight cycling on incidental diabetes in middle-aged individuals. For a sensitivity analysis, we therefore restricted the analyses to a population aged 45 to 64 years old. All statistical analyses used SAS statistical software (version 9.3, SAS Institute, NC, USA). The descriptive statistics were reported as the means and standard deviations (SD). All reported p-values were two-sided; p-values of < 0.05 were considered statistically significant.

For peer review only

RESULTS

Participants

In total, 10,094 men and 10,614 women were enrolled from the Tokyo urban area, and 4,818 men and 4,852 women from Yamanashi Prefecture. Their baseline characteristics are shown in Table 1 and Supplementary.

Table 1. Baseline characteristics of the participants from urban (Tokyo) and rural (Yamanashi Prefecture) regions of Japan

Characteristics, mean (standard deviation)	Men	Women
**Urban region**		
No.	10,094	10,614
Age, years	49.6 (11.9)	48.3 (11.3)
Weight, kg	68.8 (9.7)	52.3 (7.4)

Height, cm	170.4 (6.1)	157.7 (5.6)
Body mass index, kg/m^2	23.7 (2.8)	21.0 (2.9)
HbA1c, %	5.4 (0.3)	5.3 (0.4)
(HbA1c, mmol/mol)	35.3 (3.7)	35.0 (3.8)
Fasting plasma glucose, mg/dL	100.8 (8.6)	94.5 (8.0)
(Fasting plasma glucose, mmol/L)	5.6 (0.5)	5.2 (0.4)
**Rural region**		
No.	4,818	4,852
Age, years	51.2 (10.3)	52.1 (9.4)
Weight, kg	65.7 (9.1)	53.1 (7.5)
Height, cm	168.1 (6.2)	154.8 (5.6)
Body mass index, kg/m^2	23.2 (2.7)	22.1 (2.9)
HbA1c, %	5.3 (0.3)	5.3 (0.3)
(HbA1c, mmol/mol)	34.3 (3.8)	34.4 (3.5)
Fasting plasma glucose, mg/dL	96.3 (9.0)	93.2 (8.5)
(Fasting plasma glucose, mmol/L)	5.3 (0.5)	5.2 (0.5)

For peer review only

Risk of diabetes in urban and rural Japan

Table 2 shows means (SDs) of follow-up duration and numbers of measurements in each weight change group. The numbers of measurements were smallest in the stable group in both sexes and both regions. Tables 3 and 4 show the incidence of diabetes and the odds ratios (ORs) for each explanatory variable in the urban and rural regions.

Table 2. Means (standard deviations) of follow-up duration and numbers of measurements in the groups of weight change patterns

Urban region	Weight change pattern over 10	Follow-up duration,	No. of measurements

For peer review only - http://bmjopen.bmj.com/site/about/guidelines.xhtml

	years	years	
Men	Sustained loss	7.7 (1.9)	5.8 (1.6)
	Loss-gain	8.2 (1.2)	5.8 (1.4)
	Stable	7.9 (1.7)	4.9 (1.7)
	Gain-loss	8.2 (1.2)	5.7 (1.4)
	Sustained gain	7.4 (1.9)	5.4 (1.6)
Women	Sustained loss	7.9 (1.7)	6.0 (1.5)
	Loss-gain	8.2 (1.2)	5.9 (1.4)
	Stable	7.9 (1.6)	5.0 (1.7)
	Gain-loss	8.2 (1.1)	5.9 (1.4)
	Sustained gain	7.5 (1.9)	5.6 (1.6)
Rural region	Weight change pattern over 10 years	Follow-up duration	No. of measurements
Men	Sustained loss	7.3 (1.7)	5.2 (1.6)
	Loss-gain	7.9 (1.4)	6.2 (1.2)
	Stable	7.1 (1.9)	5.2 (1.6)
	Gain-loss	7.8 (1.4)	6.1 (1.3)
	Sustained gain	7.4 (1.6)	5.4 (1.6)
Women	Sustained loss	7.4 (1.7)	5.4 (1.5)
	Loss-gain	7.9 (1.3)	6.1 (1.3)
	Stable	7.0 (1.8)	5.0 (1.6)
	Gain-loss	7.9 (1.4)	6.0 (1.3)
	Sustained gain	7.3 (1.6)	5.1 (1.6)

Table 3. The incidence and odds ratios (95% CIs) of diabetes for different patterns of weight change over 10 years among urban residents in Japan

Exposure variables		Acquired DM / No. of subjects (incidence, %)	Crude	Multivariate
**Men (no. for multivariate analysis = 10,094)**				
Baseline age	Per 10 years	—	1.44 (1.33–1.55)	1.44 (1.29–1.61)
Weight change	< −5	7/1438 (0.5)	2.18 (1.14–4.18)	1.68 (0.75–3.80)

For peer review only - http://bmjopen.bmj.com/site/about/guidelines.xhtml

from 20 years of age, kg	−5 to +5	73/10646 (0.7)	Ref	Ref
	+5 to +10	31/3200 (1.0)	1.98 (1.45–2.72)	1.54 (1.03–2.30)
	> +10	78/2166 (3.6)	3.37 (2.54–4.48)	2.08 (1.40–3.10)
Baseline BMI, kg/m^2	< 18.5	6/387 (1.6)	1.35 (0.57–3.16)	0.88 (0.29–2.70)
	18.5–22	49/4236 (1.2)	Ref	Ref
	22–25	168/7220 (2.3)	2.04 (1.48–2.89)	1.73 (1.14–2.63)
	> 25	190/4699 (4.0)	3.60 (2.62–4.94)	2.52 (1.60–3.95)
Weight change pattern over 10 years	Sustained loss	66/1903 (3.5)	1.39 (1.03–1.87)	1.11 (0.77–1.59)
	Loss–gain	87/4644 (1.9)	0.74 (0.56–0.97)	0.63 (0.45–0.89)
	Stable	142/5621 (2.5)	Ref	Ref
	Gain–loss	38/3063 (1.2)	0.49 (0.34–0.70)	0.51 (0.32–0.82)
	Sustained gain	80/1311 (6.1)	2.51 (1.89–3.32)	3.07 (2.15–4.39)
Smoking	None	123/6385 (1.9)	Ref	Ref
	Ex-smoker	150/5940 (2.5)	1.32 (1.04–1.68)	0.97 (0.71–1.31)
	Current smoker	140/4217 (3.3)	1.75 (1.37–2.23)	1.73 (1.25–2.34)
Alcohol drinking	None	69/2250 (3.1)	Ref	Ref
	Sometimes	48/1667 (2.9)	0.94 (0.65–1.36)	0.96 (0.66–1.40)
	Usually	156/6177 (2.5)	0.82 (0.61–1.09)	0.78 (0.58–1.05)
Amount of walking per day	Per 30 min	—	0.996 (0.92–1.08)	1.01 (0.92–1.11)
Physical activity (sessions/week)	0–1	85/3282 (2.6)	Ref	Ref
	1–2	108/4105 (2.6)	1.02 (0.76–1.36)	1.02 (0.76–1.36)
	3–5	43/1541 (2.8)	1.08 (0.74–1.57)	0.98 (0.67–1.45)
	6–7	37/1166 (3.2)	1.23 (0.83–1.82)	1.06 (0.69–1.62)
**Women (no. for multivariate analysis = 10,614)**				
Baseline age	Per 10 years	—	1.66 (1.48–1.87)	1.78 (1.50–2.12)
Weight change from 20 years of age, kg	< −5	11/439 (2.5)	0.71 (0.33–1.54)	0.48 (0.18–1.25)
	−5 to +5	61/5243 (1.2)	Ref	Ref
	+5 to +10	109/4779 (2.3)	1.42 (0.93–2.16)	0.96 (0.55–1.65)
	> +10	232/6081 (3.8)	5.41 (3.92–7.47)	2.10 (1.20–3.67)
Baseline BMI, kg/m^2	< 18.5	20/2916 (0.7)	1.24 (0.74–2.08)	1.47 (0.76–2.86)
	18.5–22	52/9388 (0.6)	Ref	Ref
	22–25	60/3676 (1.6)	2.98 (2.05–4.33)	2.01 (1.21–3.34)

For peer review only - http://bmjopen.bmj.com/site/about/guidelines.xhtml

	> 25	57/1470 (3.9)	7.24 (4.95–10.59)	2.91 (1.53–5.52)
Weight change pattern over 10 years	Sustained loss	24/1661 (1.4)	2.11 (1.25–3.54)	1.48 (0.80–2.74)
	Loss–gain	32/4022 (0.7)	1.15 (0.72–1.86)	0.72 (0.39–1.34)
	Stable	36/5212 (1.3)	Ref	Ref
	Gain–loss	30/4630 (0.6)	0.94 (0.58–1.53)	1.05 (0.57–1.95)
	Sustained gain	67/1925 (3.5)	5.19 (3.45–7.80)	7.00 (4.11–11.94)
Smoking	None	156/14,194 (1.1)	Ref	Ref
	Ex-smoker	17/1789 (1.0)	0.86 (0.52–1.1.43)	0.85 (0.45–1.61)
	Current smoker	16/1467 (1.1)	0.99 (0.59–1.67)	1.20 (0.63–2.32)
Alcohol drinking	None	78/5669 (1.4)	Ref	Ref
	Sometimes	20/2035 (1.0)	0.71 (0.43–1.17)	0.91 (0.55–1.52)
	Usually	27/2910 (0.9)	0.67 (0.43–1.04)	0.95 (0.60–1.51)
Amount of walking per day	Per 30 min	—	1.01 (0.93–1.11)	1.01 (0.90–1.13)
Physical activity (sessions/week)	0–1	37/3894 (1.0)	Ref	Ref
	1–2	42/3760 (1.1)	1.18 (0.76–1.84)	1.11 (0.70–1.75)
	3–5	27/1874 (1.4)	1.52 (0.93–2.52)	1.14 (0.67–1.94)
	6–7	19/1086 (1.7)	1.86 (1.06–3.24)	1.43 (0.79–2.60)

DM, diabetes mellitus; CI, confidence interval; Ref, reference group; BMI, body mass index

Table 4. The incidence and odds ratios (95% CIs) of diabetes for different patterns of weight change over 10 years among rural residents in Japan

Exposure variables		Acquired DM / No. of subjects (incidence, %)	Crude	Multivariate
**Men (no. for multivariate analysis = 4,818)**				
Baseline age	Per 10 years	—	1.36 (1.08–1.72)	1.60 (1.24–2.05)
Baseline BMI, kg/m^2	< 18.5	0/167 (0)	—	—
	18.5–22	10/1425 (0.7)	Ref	Ref
	22–25	21/2079 (1.0)	1.44 (0.68–3.08)	1.69 (0.79–3.63)
	> 25	35/1148 (3.1)	4.45 (2.19–9.03)	5.81 (2.82–11.97)

For peer review only

Weight change pattern over 10 years	Sustained loss	4/725 (0.6)	0.43 (0.15–1.25)	0.36 (0.12–1.05)
	Loss–gain	13/681 (1.9)	1.50 (0.75–3.00)	1.58 (0.78–3.17)
	Stable	22/1719 (1.3)	Ref	Ref
	Gain–loss	4/916 (0.4)	0.34 (0.12–0.99)	0.44 (0.15–1.29)
	Sustained gain	23/778 (3.0)	2.35 (1.30–4.24)	3.15 (1.70–5.83)
Smoking	None	23/1695 (1.4)	Ref	Ref
	Ex-smoker	8/998 (0.8)	0.59 (0.26–1.32)	0.72 (0.32–1.64)
	Current smoker	35/2126 (1.7)	1.22 (0.72–2.07)	1.50 (0.85–2.62)
Drinking	None	11/1071 (1.0)	Ref	Ref
	Drinker	55/3748 (1.5)	1.44 (0.75–2.75)	1.52 (0.79–2.94)
**Women (no. for multivariate analysis = 4,852)**				
Baseline age	Per 10 years	—	1.43 (0.99–2.06)	1.23 (0.83–1.84)
Baseline BMI, kg/m^2	< 18.5	0/411 (0)	—	—
	18.5–22	7/2135 (0.3)	Ref	Ref
	22–25	14/1563 (0.9)	2.75 (1.11–6.82)	2.69 (1.07–6.75)
	> 25	13/744 (1.8)	5.41 (2.15–13.60)	5.29 (2.07–13.51)
Weight change pattern over 10 years	Sustained loss	3/863 (0.3)	0.37 (0.11–1.29)	0.32 (0.09–1.10)
	Loss–gain	3/757 (0.4)	0.42 (0.12–1.47)	0.41 (0.12–1.44)
	Stable	15/1615 (0.9)	Ref	Ref
	Gain–loss	5/827 (0.6)	0.65 (0.24–1.79)	0.77 (0.28–2.14)
	Sustained gain	8/791 (1.0)	1.09 (0.46–2.58)	1.43 (0.59–3.48)
Smoking	None	2/646 (0.3)	Ref	Ref
	Ex-smoker	29/3822 (0.8)	2.45 (0.58–10.31)	2.19 (0.52–9.26)
	Current smoker	3/375 (0.8)	2.60 (0.43–15.60)	3.33 (0.55–20.28)
Drinking	None	31/3542 (0.9)	Ref	Ref
	Drinker	3/1311 (0.2)	0.26 (0.08–0.85)	0.29 (0.09–0.95)

DM, diabetes mellitus; CI, confidence interval; Ref, reference group; BMI, body mass index

Table 5 shows the risk of diabetes linked to weight cycling in the middle-aged population.

For peer review only

For peer review only - http://bmjopen.bmj.com/site/about/guidelines.xhtml

Table 5. The incidence and odds ratios of diabetes for different patterns of weight change over 10 years in middle-aged people (45–64 years) in Japan

Exposure variables		Acquired DM / No. of subjects (incidence, %)	Odds ratio (95% CI)	Adjusted odds ratio (95% CI)
**Urban middle-aged men (no. for multivariate analysis = 4,882)**				
Weight change pattern over 10 years	Sustained loss	35/981 (3.6)	1.04 (0.65–1.67)	0.97 (0.60–1.55)
	Loss-gain	62/2203 (2.8)	0.79 (0.53–1.18)	0.74 (0.49–1.11)
	Stable	100/2749 (3.6)	Ref	Ref
	Gain-loss	26/1098 (2.4)	0.56 (0.32–0.999)	0.57 (0.32–1.01)
	Sustained gain	50/489 (10.2)	3.09 (2.00–4.76)	3.13 (2.00–4.89)
**Urban middle-aged women (no. for multivariate analysis = 5,053)**				
Weight change pattern over 10 years	Sustained loss	15/994 (1.5)	1.30 (0.62–2.73)	1.13 (0.53–2.41)
	Loss-gain	20/1992 (1.0)	0.87 (0.43–1.77)	0.76 (0.37–1.57)
	Stable	24/2285 (1.1)	Ref	Ref
	Gain-loss	17/1642 (1.0)	0.74 (0.34–1.62)	0.80 (0.36–1.77)
	Sustained gain	40/603 (6.6)	5.62 (3.05–10.37)	6.97 (3.67–13.25)
**Rural middle-aged men (no. for multivariate analysis = 2,937)**				
Weight change pattern over 10 years	Sustained loss	3/447 (0.7)	0.48 (0.14–1.66)	0.42 (0.12–1.47)
	Loss-gain	11/449 (2.5)	1.78 (0.81–3.91)	1.76 (0.80–3.87)
	Stable	15/1078 (1.4)	Ref	Ref
	Gain-loss	4/546 (0.7)	0.52 (0.17–1.58)	0.58 (0.19–1.77)
	Sustained gain	12/417 (2.9)	2.10 (0.98–4.53)	2.48 (1.12–5.49)
**Rural middle-aged women (no. for multivariate analysis = 3,347)**				
Weight change pattern over 10 years	Sustained loss	1/638 (0.2)	0.16 (0.02–1.25)	0.14 (0.02–1.01)
	Loss-gain	3/579 (0.5)	0.54 (0.15–1.92)	0.54 (0.15–1.94)
	Stable	11/1140 (1.0)	Ref	Ref
	Gain-loss	4/558 (0.7)	0.74 (0.24–2.34)	0.82 (0.26–2.62)
	Sustained gain	5/432 (1.2)	1.20 (0.42–3.48)	1.30 (0.44–3.84)

Multivariable logistic regression-calculated odds ratios with an adjustment for baseline age and BMI, weight change from 20 years of age, smoking and drinking habits, amount of walking per day and sessions of physical activity per week in the urban data, and baseline age, BMI and smoking and drinking habits in the rural data.

For peer review only

For peer review only - http://bmjopen.bmj.com/site/about/guidelines.xhtml

For peer review only

For peer review only - http://bmjopen.bmj.com/site/about/guidelines.xhtml

DISCUSSION

The data for the Japanese urban region suggest that the risk of diabetes in male weight cyclers was significantly lower than in those who maintained a stable weight (Table 3). The diabetes risk for female weight cyclers in the urban region was non-significantly lower than or similar to the risk for those maintaining a stable weight. The data for the rural region suggest no difference in diabetes risk for weight-cycling men and a non-significantly lower risk for women compared with maintaining a stable weight (Table 4). These results are reinforced by a sensitivity analysis focusing on middle-aged individuals, yielding almost identical non-significant ORs (Table 5).

These observations are not consistent with those from previous studies of Western populations.[4-6 21] These studies showed that weight cycling significantly or non-significantly increased the risk of diabetes for point estimates. In the Framingham Heart Study, approximately 1 kg/m^2 of weight cycling in middle-aged Americans carried a hazard ratio of 1.1 (95% CI: 0.8–1.5) for the risk of diabetes after adjusting for sex and BMI at 25 years of age.[4] In American middle-aged women examined in the National Health and Nutrition Examination Survey, weight cycling of 4.5–9.1 kg and 9.1–22.2 kg with intentional weight loss three or more times in 4 years carried ORs for the risk of diabetes of 1.11 (95% CI: 0.89–1.37) and 1.39 (95% CI: 0.90–2.13).[5] A study from a cohort of medical students at the Johns Hopkins University School of Medicine reported that the highest quartile of BMI variability for ages between 25 and 45 years had an OR of 2.1 (95% CI: 1.0–4.6) for the risk of diabetes after 50 years, compared with the other three quartiles.[21] In a large German cohort, weight cycling of ≥ 1.5 kg/year was significantly associated with an adjusted hazard ratio of 1.34.[6]

For peer review only - http://bmjopen.bmj.com/site/about/guidelines.xhtml

For peer review only

The disparity between the results of this study and the Western studies may be because of ethnic differences in diet,[22] the capacity to gain weight[23] and self-consciousness about body weight.[24] Further research is needed to explore why the relationship between weight cycling and risk of diabetes is reversed for Western and East Asian populations. It may be attributable to different motivations to lose weight because of different cultural views on dieting and body self-consciousness.[25] Those East Asians who try to lose weight may be more concerned about the poor health outcomes of being overweight. The Westerners in the studies who tried to lose weight may have been more likely to lose and regain a great deal of weight, and therefore potentially ran the risk of poor health outcomes.

The urban data in this study showed that an increase of more than +5 kg above weight at 20 years old increased the risk of developing diabetes with a dose–response relationship in both men and women (Table 3). An increase of more than +10 kg from weight at 20 years old more than doubled the risk of diabetes compared with maintaining weight within ±5 kg. These results agree with the study involving a US cohort, which reported a relative risk of 3.2 (95% CI: 1.4–7.4) for the highest quartile of an increase in BMI from 25 to 45 years of age in comparison with the other three quartiles.[21] A dose–response relationship of weight change from that at 20 years old to the risk of diabetes was seen in urban Japanese women, with ORs of 0.48 (95% CI: 0.18–1.25) for a change of less than 5 kg, 0.96 (95% CI: 0.55–1.65) for an increase of between 5 and 10 kg and 2.10 (95% CI: 1.20–3.67) for an increase greater than 10 kg (Table 3). In contrast, Japanese men who lost 5 kg or more of body weight between the age of 20 years and early middle age had an OR of 1.68 (95% CI: 0.75–3.80). This

For peer review only - http://bmjopen.bmj.com/site/about/guidelines.xhtml

paradoxically increased OR was probably because of the small number of participants (seven) who developed diabetes among a weight loss group of 1,438 people.

This study had several limitations. The first of these was the threshold of ±4% weight change in 10 years. This was drawn from a study in the United Kingdom on the association between weight change and the risk of diabetes.[26] It was calculated as approximately one BMI unit in Japanese people with a mean BMI of 23 kg/m^2. However, the threshold for categorisation of weight cycling is likely to vary by mean BMI in different ethnicities, because of different insulin sensitivities.[13 27] Second, we did not evaluate insulin sensitivity. Measuring fasting plasma glucose and insulin concentration to calculate HOMA-IR,[28] an index of insulin resistance, would have allowed us to assess the association between weight cycling and the physiological hazard of diabetes. Third, the weight changes recorded in the rural region may have been misclassified because of missing data from the years when participants did not undergo a medical check-up. However, this misclassification would have biased the ORs towards the null hypothesis, and we believe that this would therefore not change the conclusions for the rural region. Fourth, we could not examine whether weight cycling was intentional. We consider, however, that a subset of unintentional weight loss could be attributed to metabolic diseases, and patients with such diseases would not usually be able to regain the weight within a short time. Fifth, the follow-up duration and the numbers of weight measurements varied among the weight change groups. However, because perfect categorisation of weight changes over time would be impossible, we think that our study design enables us to answer the study question. Sixth, the urban data for weight at 20 years old were self-reported by participants. They

For peer review only - http://bmjopen.bmj.com/site/about/guidelines.xhtml

may therefore have been affected by recall bias. Seventh, a subset of the diagnoses in this study were not made by physicians but via an epidemiological criteria.[17] However, most observational studies rely on epidemiological criteria for detecting diabetes, and the use of a consistent diagnostic criterion allows researchers to compare the risk of diabetes onset between the reference group and groups of interest. Last, this study lacks any information about lifestyle, including diet, marital status, job type and car ownership. All these may partly explain the association between weight cycling and diabetes.

This study also has several strengths. First, to the best of our knowledge, it is the first study to explore the relationship between weight cycling and the risk of diabetes in Asians. The relationship found in this study was almost directly opposite to that found in US studies, so further research in East Asians is necessary to confirm the findings. Next, this study was conducted in two different populations (urban and rural residents). The ORs of the risk of diabetes were 1.05 (95% CI: 0.57–1.95) in the urban women with weight gain-loss (Table 3) and 1.58 (95% CI: 0.78–3.17) in rural men with weight loss-gain (Table 4). All other weight-cycling patterns for both sexes in both urban and rural regions were negatively correlated with the risk of diabetes, although some relationships were not statistically significant (Tables 3 and 4). Third, the study included a large number of participants across both sexes (approximately 10,000 in the urban region and 5,000 in the rural region). Its findings are therefore likely to be generalisable to Japanese and East Asian weight cyclers.

This study could help public health practitioners and on-site clinical professionals prevent diabetes in the general population. A sustained weight gain of more than 4%

For peer review only - http://bmjopen.bmj.com/site/about/guidelines.xhtml

over 10 years in middle age (Table 5) and more than 5 kg of additional weight gain since the age of 20 years (Table 3) may both carry an increased risk of diabetes for both sexes. Middle-aged people can easily undergo such a small weight gain over the short- or long-term, so even non-diabetic people within the normal BMI range should be cautious about the risks resulting from small weight gains throughout their lifetime. An interventional study indicated that weight loss (−1.8 kg/m^2 of BMI in a diet intervention group and −3.3 kg/m^2 of BMI in a diet-and-exercise intervention group) improved insulin sensitivity in Japanese patients with obesity and type 2 diabetes.[29] Improved insulin sensitivity was also observed in Americans who maintained their weight with treadmill-based exercise and no alteration in their diet.[30] Studies have also demonstrated that building muscle through exercising without changing weight improves insulin sensitivity.[31]

While not statistically significant, two weight-cycling patterns were found to result in an increased risk of diabetes (Tables 3 and 4). Better insulin sensitivity leads directly to a decreased risk of diabetes, so these results may be because of differences in how the participants lost or gained weight (i.e. whether they lost or gained fat or muscle mass). Recent studies have indicated that sarcopenia, the loss of skeletal muscle alone or with increased fat mass in ageing, is a leading cause of death in old age.[32] The results of our study, in the context of previous studies, suggest that over both the short- and long-term, people might reduce their risk of diabetes by losing fat and maintaining muscle mass.

For peer review only - http://bmjopen.bmj.com/site/about/guidelines.xhtml

283 **CONCLUSIONS**

284 In urban men in Japan, weight cycling was associated with a significant reduction in the
285 risk of diabetes. No clear association was seen in either urban women or in men and
286 women living in the rural region. The results were different from those seen in Western
287 countries and may be attributed to differences in diet, endocrinological capacity to gain
288 weight and weight-consciousness. The risk of diabetes seems to increase linearly with
289 weight gain from the age of 20 years in urban Japanese men and women. A study that
290 includes the measurement of insulin sensitivity is necessary to confirm these results and
291 to improve the understanding of the risks for East Asian weight cyclers.

292 **(2,718 words; limit of 4,000 words)**

For peer review only

Acknowledgments We thank all participants in this study and staff at the St. Luke's International Hospital and the Yamanashi Koseiren Health Care Center for their support, which made this study possible.

Competing interests None declared.

Funding This work was supported by the Ministry of Education, Culture, Sports, Science and Technology of Japan (MEXT) (KAKENHI grant number: JP15K08730 and JP15K15221). The funder had no role in study design, analysis, decision to publish or preparation of the manuscript.

Contributors ZY, YY, MT and OT: setting up the study and data collection. HY, ZY, MM and AT: designing the study. HY and AT: data analysis. HY: writing and revising the draft. ZY, SO, MM, YA and HY: development of the discussion section. All authors read and approved the final manuscript.

Ethics approval The ethics committee of the School of Medicine, University of Yamanashi approved this study (approval number: H27-1417).

Data sharing statement No additional data are available.

For peer review only

REFERENCES

1. Nanri A, Mizoue T, Takahashi Y, et al. Association of weight change in different periods of adulthood with risk of type 2 diabetes in Japanese men and women: the Japan Public Health Center-Based Prospective Study. *J Epidemiol Commun H* 2011;**65**:1104–10.

2. Wannamethee SG, Shaper AG. Weight change and duration of overweight and obesity in the incidence of type 2 diabetes. *Diabetes Care* 1999;**22**:1266–72.

3. Ford ES, Williamson DF, Liu SM. Weight change and diabetes incidence: findings from a national cohort of US adults. *Am J Epidemiol* 1997;**146**:214–22.

4. Waring ME, Eaton CB, Lasater TM, et al. Incident diabetes in relation to weight patterns during middle age. *Am J Epidemiol* 2010;**171**:550–6.

5. Field AE, Manson JE, Laird N, et al. Weight cycling and the risk of developing type 2 diabetes among adult women in the United States. *Obes Res* 2004;**12**:267–74.

6. Neamat-Allah J, Barrdahl M, Hüsing A, et al. Weight cycling and the risk of type 2 diabetes in the EPIC-Germany cohort. *Diabetologia* 2015;**58**:2718–25.

7. French S, Folsom A, Jeffery R, Zheng W, Mink P, Baxter J. Weight variability and incident disease in older women: the Iowa Women's Health Study. *Int J Obes Relat Metab Disord* 1997;**21**:217–23.

8. Morris RD, Rimm AA. Long-term weight fluctuation and non-insulin-dependent diabetes mellitus in white women. *Ann Epidemiol* 1992;**2**:657–64.

9. Mukai T, Crago M, Shisslak CM. Eating attitudes and weight preoccupation among female high school students in Japan. *J Child Psychol Psychiatry* 1994;**35**:677–88.

For peer review only

For peer review only - http://bmjopen.bmj.com/site/about/guidelines.xhtml

10. Swami V, Frederick DA, Aavik T, et al. The attractive female body weight and female body dissatisfaction in 26 countries across 10 world regions: results of the International Body Project I. *Personal Soc Psychol Bull* 2010;**36**:309–25.

11. Chisuwa N, O'Dea JA. Body image and eating disorders amongst Japanese adolescents. a review of the literature. *Appetite* 2010;**54**:5–15.

12. Xu X, Mellor D, Kiehne M, et al. Body dissatisfaction, engagement in body change behaviours and sociocultural influences on body image among Chinese adolescents. *Body Image* 2010;**7**:156–64.

13. Kodama K, Tojjar D, Yamada S, et al. Ethnic differences in the relationship between insulin sensitivity and insulin response: a systematic review and meta-analysis. *Diabetes Care* 2013;**36**:1789–96.

14. Matsushita Y, Yoshiike N, Kaneda F, Yoshita K, Takimoto H. Trends in childhood obesity in Japan over the last 25 years from the national nutrition survey. *Obes Res* 2004;**12**:205–14.

15. Yoshiike N, Seino F, Tajima S, et al. Twenty□year changes in the prevalence of overweight in Japanese adults: The National Nutrition Survey 1976–95. *Obes Rev* 2002;**3**:183–90.

16. Ministry of Health, Labour and Welfare of Japan. Industrial Safety and Health Act (English version). 1972. http://www.japaneselawtranslation.go.jp/law/detail/?printID=&id=1926&re=&vm=02 (accessed 15 March 2017).

17. American Diabetes Association. Diagnosis and classification of diabetes mellitus. *Diabetes Care* 2010;**33**:S62–S69.

For peer review only

For peer review only - http://bmjopen.bmj.com/site/about/guidelines.xhtml

18. The International Expert Committee. International Expert Committee report on the role of the A1C assay in the diagnosis of diabetes. *Diabetes Care* 2009;**32**:1327–34.

19. Kaufman J, Poole C. Looking back on “causal thinking in the health sciences”. *Annu Rev Publ Health* 2000;**21**:101-19.

20. NCD Risk Factor Collaboration. Worldwide trends in diabetes since 1980: a pooled analysis of 751 population-based studies with 4· 4 million participants. *Lancet* 2016;**387**:1513–30.

21. Brancati FL, Wang N, Mead LA, et al. Body weight patterns from 20 to 49 years of age and subsequent risk for diabetes mellitus: the Johns Hopkins Precursors Study. *Arch Int Med* 1999;**159**:957–63.

22. Slocum R. Race in the study of food. *Prog Hum Geogr* 2011;**35**:303–27.

23. The DECODE–DECODA Study Group. Age, body mass index and type 2 diabetes—associations modified by ethnicity. *Diabetologia* 2003;**46**:1063–70.

24. Mintz LB, Kashubeck S. Body image and disordered eating among Asian American and Caucasian college students an examination of race and gender differences. *Psychol Women Q* 1999;**23**:781–96.

25. Frederick DA, Forbes GB, Grigorian KE, et al. The UCLA Body Project I: gender and ethnic differences in self-objectification and body satisfaction among 2,206 undergraduates. *Sex Roles* 2007;**57**:317–27.

26. Wannamethee SG, Shaper AG, Walker M. Overweight and obesity and weight change in middle aged men: impact on cardiovascular disease and diabetes. *J Epidemiol Commun H* 2005;**59**:134–9.

For peer review only

For peer review only - http://bmjopen.bmj.com/site/about/guidelines.xhtml

27. Ntuk UE, Gill JMR, Mackay DF, et al. Ethnic-specific obesity cutoffs for diabetes risk: cross-sectional study of 490,288 UK Biobank participants. *Diabetes Care* 2014;**37**:2500–7.

28. Matthews D, Hosker J, Rudenski A, et al. Homeostasis model assessment: insulin resistance and β-cell function from fasting plasma glucose and insulin concentrations in man. *Diabetologia* 1985;**28**:412–9.

29. Yamanouchi K, Shinozaki T, Chikada K, et al. Daily walking combined with diet therapy is a useful means for obese NIDDM patients not only to reduce body weight but also to improve insulin sensitivity. *Diabetes Care* 1995;**18**:775–8.

30. Duncan GE, Perri MG, Theriaque DW, et al. Exercise training, without weight loss, increases insulin sensitivity and postheparin plasma lipase activity in previously sedentary adults. *Diabetes Care* 2003;**26**:557–62.

31. Nassis GP, Papantakou K, Skenderi K, et al. Aerobic exercise training improves insulin sensitivity without changes in body weight, body fat, adiponectin, and inflammatory markers in overweight and obese girls. *Metabolism* 2005;**54**:1472–9.

32. Fielding RA, Vellas B, Evans WJ, et al. Sarcopenia: an undiagnosed condition in older adults. Current consensus definition: prevalence, etiology, and consequences. International Working Group on Sarcopenia. *J Am Med Dir Assoc* 2011;**12**:249–56.

For peer review only

Figure 1. How participants were categorised into the five weight change patterns

For peer review only

For peer review only - http://bmjopen.bmj.com/site/about/guidelines.xhtml

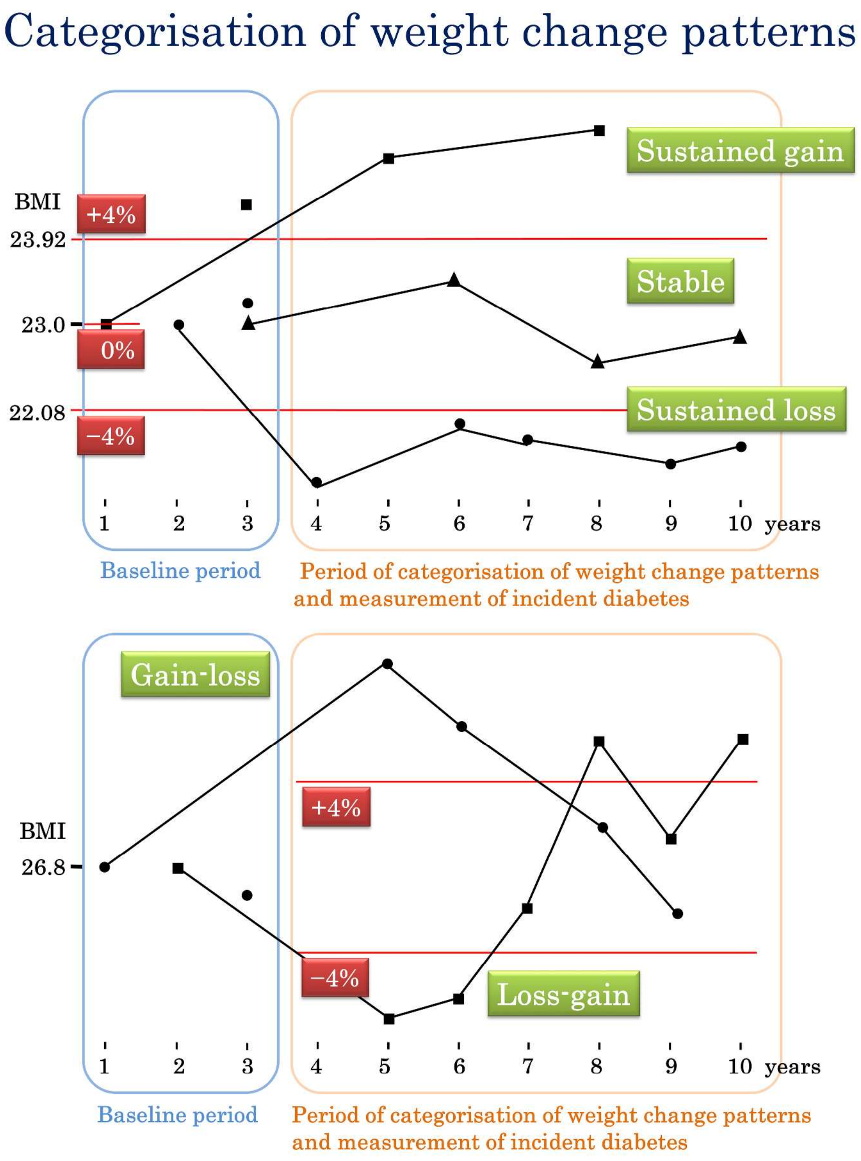

How participants were categorised into the five weight change patterns
Figure 1
339x454mm (300 x 300 DPI)

Supplementary. Baseline characteristics among groups of weight change patterns over 10 years

**Men in urban Japan (n = 10,094)**	Sustained loss	Loss-gain	Stable	Gain-loss	Sustained gain
Age, years	52.3 (12.2)	51.2 (12.0)	50.3 (11.3)	46.0 (11.4)	44.9 (11.3)
Weight, kg	70.8 (10.4)	69.8 (9.5)	68.3 (9.0)	67.3 (9.5)	67.8 (10.8)
Height, cm	170.1 (6.2)	170.3 (6.1)	170.2 (6.0)	170.7 (6.1)	171.2 (6.1)
Body mass index, kg/m2	24.4 (3.0)	24.0 (2.7)	23.5 (2.6)	23.1 (2.8)	23.1 (3.2)
HbA1c, %	5.4 (0.4)	5.4 (0.3)	5.4 (0.3)	5.3 (0.3)	5.3 (0.3)
HbA1c, mmol/mol	35.9 (3.9)	35.7 (3.7)	35.3 (3.7)	34.6 (3.7)	34.6 (3.4)
Fasting plasma glucose, mg/dL	102.3 (9.2)	101.5 (8.8)	101.0 (8.5)	99.0 (8.0)	99.8 (8.4)
Fasting plasma glucose, mmol/L	5.7 (0.5)	5.6 (0.5)	5.6 (0.5)	5.5 (0.4)	5.5 (0.5)
Weight change from 20 years of age, kg	+9.7 (9.1)	+9.0 (8.1)	+8.2 (7.9)	+7.0 (8.4)	+7.0 (20.5)
Current smoking, %	20.0	22.8	23.6	31.3	33.7
Usually drinking alcohol, %	61.7	62.3	63.8	57.3	55.2
Duration of walking per day, min	41.0 (38.3)	41.3 (41.7)	43.3 (41.5)	40.4 (35.2)	38.6 (36.0)
Physical activity of 6–7/week, %	11.2	12.0	11.9	10.4	11.6

**Women in urban Japan (n = 10,614)**	Sustained loss	Loss-gain	Stable	Gain-loss	Sustained gain
Age, years	52.4 (10.7)	51.1 (11.5)	48.9 (11.3)	45.7 (10.6)	43.5 (9.8)
Weight, kg	53.8 (8.0)	53.4 (7.8)	51.7 (7.0)	51.8 (7.1)	51.6 (7.4)
Height, cm	156.9 (5.7)	157.2 (5.9)	157.8 (5.6)	158.1 (5.5)	158.4 (5.3)
Body mass index, kg/m2	21.8 (3.0)	21.6 (3.0)	20.8 (2.7)	20.7 (2.7)	20.6 (2.8)
HbA1c, %	5.5 (0.4)	5.4 (0.4)	5.4 (0.3)	5.3 (0.3)	5.3 (0.3)
HbA1c, mmol/mol	36.1 (4.1)	35.4 (3.8)	35.0 (3.8)	34.4 (3.6)	34.0 (3.7)
Fasting plasma glucose, mg/dL	96.5 (8.9)	95.7 (8.1)	94.6 (8.0)	93.2 (7.3)	92.8 (7.3)

For peer review only

For peer review only - http://bmjopen.bmj.com/site/about/guidelines.xhtml

Fasting plasma glucose, mmol/L	5.4 (0.5)	5.3 (0.4)	5.3 (0.4)	5.2 (0.4)	5.2 (1.4)
Weight change from 20 years of age, kg	+4.1 (7.4)	+3.8 (7.3)	+2.4 (7.1)	+2.6 (7.1)	+2.2 (7.5)
Current smoking, %	6.6	6.7	5.7	9.9	11.8
Usually drinking alcohol, %	23.7	26.7	27.3	29.4	28.3
Duration of walking per day, min	42.9 (49.4)	41.2 (49.8)	42.3 (40.9)	41.5 (40.5)	43.8 (51.9)
Physical activity of 6–7/week, %	12.1	10.6	11.3	8.6	8.8
**Men in rural Japan (n = 4,818)**	Sustained loss	Loss-gain	Stable	Gain-loss	Sustained gain
Age, years	53.5 (10.3)	51.7 (9.8)	52.3 (10.1)	49.2 (10.1)	48.3 (10.5)
Weight, kg	67.2 (9.0)	66.2 (8.7)	66.0 (9.1)	64.7 (8.6)	64.3 (9.9)
Height, cm	167.5 (6.2)	168.0 (6.0)	167.8 (6.2)	168.5 (6.2)	168.8 (6.5)
Body mass index, kg/m2	23.9 (2.6)	23.4 (2.6)	23.4 (2.7)	22.8 (2.5)	22.5 (2.9)
HbA1c, %	5.3 (0.4)	5.3 (0.4)	5.3 (0.3)	5.2 (0.3)	5.2 (0.3)
HbA1c, mmol/mol	34.7 (3.9)	34.6 (3.9)	34.3 (3.7)	33.9 (3.8)	33.8 (3.5)
Fasting plasma glucose, mg/dL	97.5 (9.2)	96.4 (9.0)	96.9 (9.1)	95.1 (8.9)	95.1 (8.5)
Fasting plasma glucose, mmol/L	5.4 (0.5)	5.4 (0.5)	5.4 (0.5)	5.3 (0.5)	5.3 (0.5)
Current smoker, %	38.5	42.7	37.7	49.3	58.5
Current drinker, %	79.5	77.5	78.4	75.7	77.5
**Women in rural Japan (n = 4,852)**	Sustained loss	Loss-gain	Stable	Gain-loss	Sustained gain
Age, years	54.9 (9.0)	52.8 (8.6)	53.1 (9.2)	50.7 (9.2)	48.2 (9.9)
Weight, kg	54.0 (8.1)	53.6 (7.5)	52.9 (6.9)	52.6 (7.6)	52.6 (7.8)
Height, cm	154.1 (5.6)	154.8 (5.8)	154.6 (5.4)	155.3 (5.5)	155.7 (5.6)
Body mass index, kg/m2	22.7 (3.1)	22.4 (2.8)	22.1 (2.8)	21.8 (2.9)	21.7 (3.0)
HbA1c, %	5.3 (0.3)	5.3 (0.3)	5.3 (0.3)	5.3 (0.3)	5.2 (0.3)
HbA1c, mmol/mol	34.9 (3.7)	34.6 (3.4)	34.6 (3.6)	34.0 (3.4)	33.6 (3.3)

For peer review only

Fasting plasma glucose, mg/dL	94.1 (9.1)	93.0 (8.3)	93.7 (8.8)	93.0 (8.0)	91.9 (7.9)
Fasting plasma glucose, mmol/L	5.2 (0.5)	5.2 (0.5)	5.2 (0.5)	5.2 (0.4)	5.1 (0.4)
Current smoker, %	5.5	8.1	6.6	8.5	11.5
Current drinker, %	24.2	27.2	26.5	27.1	30.9

The data are presented as the mean (SD) or %.

For peer review only

STROBE Statement—checklist of items that should be included in reports of observational studies

	Item No.	Recommendation	Page No.	Relevant text from manuscript
**Title and abstract**	1	(*a*) Indicate the study's design with a commonly used term in the title or the abstract	1	Line 1–2
		(*b*) Provide in the abstract an informative and balanced summary of what was done and what was found	2	Line 23–42
**Introduction**				
Background/rationale	2	Explain the scientific background and rationale for the investigation being reported	5	Line 54–69
Objectives	3	State specific objectives, including any prespecified hypotheses	5	Line 69–71
**Methods**				
Study design	4	Present key elements of study design early in the paper	6	Line 79–80
Setting	5	Describe the setting, locations, and relevant dates, including periods of recruitment, exposure, follow-up, and data collection	6	Line 79–89
Participants	6	(*a*) *Cohort study*—Give the eligibility criteria, and the sources and methods of selection of participants. Describe methods of follow-up<br>*Case-control study*—Give the eligibility criteria, and the sources and methods of case ascertainment and control selection. Give the rationale for the choice of cases and controls<br>*Cross-sectional study*—Give the eligibility criteria, and the sources and methods of selection of participants	6	Line 80–89
		(*b*) *Cohort study*—For matched studies, give matching criteria and number of exposed and unexposed<br>*Case-control study*—For matched studies, give matching criteria and the number of controls per case		NA
Variables	7	Clearly define all outcomes, exposures, predictors, potential confounders, and effect modifiers. Give diagnostic criteria, if applicable	6	Line 93–97
Data sources/ measurement	8*	For each variable of interest, give sources of data and details of methods of assessment (measurement). Describe comparability of assessment methods if there is more than one group	7	Line 102–110
Bias	9	Describe any efforts to address potential sources of bias	5	Line 82–89
Study size	10	Explain how the study size was arrived at	8	Line 138–139

Continued on next page

For peer review only - http://bmjopen.bmj.com/site/about/guidelines.xhtml

Quantitative variables	11	Explain how quantitative variables were handled in the analyses. If applicable, describe which groupings were chosen and why	7	Line 115–117
Statistical methods	12	(*a*) Describe all statistical methods, including those used to control for confounding	7–8	Line 121–128
		(*b*) Describe any methods used to examine subgroups and interactions	8	Line 129–131
		(*c*) Explain how missing data were addressed	6	Line 87–89, 92–95
		(*d*) *Cohort study*—If applicable, explain how loss to follow-up was addressed<br>*Case-control study*—If applicable, explain how matching of cases and controls was addressed<br>*Cross-sectional study*—If applicable, describe analytical methods taking account of sampling strategy		NA
		(*e*) Describe any sensitivity analyses	8	Line 129–131
**Results**				
Participants	13*	(a) Report numbers of individuals at each stage of study—eg numbers potentially eligible, examined for eligibility, confirmed eligible, included in the study, completing follow-up, and analysed	8	Line 138–139
		(b) Give reasons for non-participation at each stage	6	Line 89–95
		(c) Consider use of a flow diagram		NA
Descriptive data	14*	(a) Give characteristics of study participants (eg demographic, clinical, social) and information on exposures and potential confounders	8–9	Table 1
		(b) Indicate number of participants with missing data for each variable of interest		NA
		(c) *Cohort study*—Summarise follow-up time (eg, average and total amount)	9–10	Table 2
Outcome data	15*	*Cohort study*—Report numbers of outcome events or summary measures over time	10–13	Tables 3 and 4
		Case-control study—Report numbers in each exposure category, or summary measures of exposure		NA
		Cross-sectional study—Report numbers of outcome events or summary measures		NA
Main results	16	(*a*) Give unadjusted estimates and, if applicable, confounder-adjusted estimates and their precision (eg, 95% confidence interval). Make clear which confounders were adjusted for and why they were included	10–13	Tables 3 and 4
		(*b*) Report category boundaries when continuous variables were categorized	10–14 (BMI)	Tables 3–5
		(*c*) If relevant, consider translating estimates of relative risk into absolute risk for a meaningful time period		NA

For peer review only - http://bmjopen.bmj.com/site/about/guidelines.xhtml

For peer review only

Continued on next page Other analyses	17	Report other analyses done—eg analyses of subgroups and interactions, and sensitivity analyses	14–15	Table 5
**Discussion**				
Key results	18	Summarise key results with reference to study objectives	16	Line 170–177
Limitations	19	Discuss limitations of the study, taking into account sources of potential bias or imprecision. Discuss both direction and magnitude of any potential bias	18–19	Line 217–245
Interpretation	20	Give a cautious overall interpretation of results considering objectives, limitations, multiplicity of analyses, results from similar studies, and other relevant evidence	19–20	Line 259–272
Generalisability	21	Discuss the generalisability (external validity) of the study results	19	Line 255–258
**Other information**				
Funding	22	Give the source of funding and the role of the funders for the present study and, if applicable, for the original study on which the present article is based	22	Line 297–300

*Give information separately for cases and controls in case-control studies and, if applicable, for exposed and unexposed groups in cohort and cross-sectional studies.

Note: An Explanation and Elaboration article discusses each checklist item and gives methodological background and published examples of transparent reporting. The STROBE checklist is best used in conjunction with this article (freely available on the Web sites of PLoS Medicine at http://www.plosmedicine.org/, Annals of Internal Medicine at http://www.annals.org/, and Epidemiology at http://www.epidem.com/). Information on the STROBE Initiative is available at www.strobe-statement.org.

For peer review only - http://bmjopen.bmj.com/site/about/guidelines.xhtml
